# Effect of Chinese herbal medicine (CHM) as an adjunctive therapy in distinct stages of patients with COVID-19: A systematic review and meta-analysis

**DOI:** 10.1371/journal.pone.0318892

**Published:** 2025-02-13

**Authors:** Jin-Min Gu, Shu-Nan Zhang, Si-Yao Xiao, Ming-Yue Jia, Jian-Feng Tu, Gui-Ling Han

**Affiliations:** 1 Institute of Clinical Medicine, Beijing University of Chinese Medicine, Beijing, China; 2 Respiratory Department, China-Japan Friendship Hospital, Beijing, China; 3 School of Acupuncture-Moxibustion and Tuina, Beijing University of Chinese Medicine, Beijing, China; Endeavour College of Natural Health, AUSTRALIA

## Abstract

**Background:**

The pandemic of coronavirus disease 2019 (COVID-19), caused by severe acute respiratory disease coronavirus 2 (SARS-CoV-2), has led to millions of infected cases and deaths worldwide. Clinical practice and clinical trials in China suggested that integrated Chinese herbal medicine (CHM) and conventional Western monotherapy (ICW) have achieved significant clinical effectiveness in treating COVID-19 patients.

**Objectives:**

This article aims to systematically evaluate the effects of ICW in treating patients at distinct stages of COVID-19. The most frequently used components of the CHM formulas have been summarized to define the most promising drug candidates.

**Methods:**

In this meta-analysis, seven databases up to May 20, 2024, were systematically searched to collect relevant randomized controlled trials (RCTs) and cohort studies (CSs). Difference in mean (MD) or ratio risk (RR) with 95% confidence interval (CI) was utilized for data processing analysis.

**Results:**

A total of 46 studies, consisting of 24 RCTs and 22 CSs, and 10492 patients were included. ICW group showed significant improvement over the conventional Western monotherapy (CWM) group at all stages of COVID-19 patients. ICW therapy was effective in improving recovery rate of chest CT (RR = 1.21, 95%CI [1.13,1.29]), shortening negativity time of nucleic acid (MD = -2.14,95% CI [-3.70, -0.58]), suppressing the transition of mild/moderate patients into severe conditions (RR = 0.45, 95% CI [0.33,0.62]), and reducing mortality (RR = 0.45, 95% CI [0.37,0.55]) for severe/critical COVID-19. Furthermore, compared with severe/critical patients, mild/moderate COVID-19 patients proved more effective after being treated with ICW therapy. They had a higher recovery rate of chest CT manifestations (75.4% vs. 69.1%), shorter negativity time of nucleic acid (9.21 d vs. 14.89 d), reduced time to clinical symptom reduction (3.85d vs. 11d) and shortened days of hospital stays (15.9d vs 19.1d). As for inflammatory markers analysis, ICW regimens decreased the level of lymphocytes in mild/moderate and severe/critical patients (MD = -0.15, 95% CI [-0.18, -0.13]), but no statistical difference was observed in white blood cell count and neutrophils count (MD = 0.02, 95% CI [-0.14, -0.18]; MD = 0.22,95% CI [-0.7, 1.15], respectively). A different tendency was found in the C-reactive protein level, which significantly decreased at the early stage of COVID-19 in the ICW group (MD = 2.56, 95%CI [1.28,3.83]).

**Conclusion:**

This meta-analysis demonstrates the significant superiority of ICW over single western monotherapy in improving clinical efficacy at distinct stages of Chinese COVID-19 patients. Subgroup analysis further showed that the earlier intervention of CHM may contribute to a better therapeutic effect.

**Trial registration:**

**PROSPERO ID**: CRD42023401200.

## Introduction

The novel severe acute respiratory syndrome coronavirus 2 (SARS-CoV-2) is the pathogen of coronavirus disease 2019 (COVID-19), a fast-spreading disease that affects millions of people around the world [1]. Mild patients presented with fever, fatigue, and dry cough, and severe patients may experience dyspnea, acute respiratory distress syndrome (ARDS), and even multiple organ dysfunction syndrome (MODS) [2].

A variety of antiviral drugs, monoclonal antibodies, and immunomodulatory drugs have been proposed as treatments for sars-cov-2 infection [3–5]. Especially Paxlovid, a new oral novel coronavirus treatment drug, with the advantages of being not easily resistant to the drug and effective against mutants, has reduced the risk of hospitalization or death of COVID-19 patients by 89% [6]. However, Paxlovid contains CYP3A4 enzyme inhibitor ritonavir, which may interact with many drugs and affect liver and kidney function, mainly limiting drug users and increasing the drug risk of older adults with underlying diseases [7].

According to the Chinese Diagnosis and Treatment Protocol for COVID-19 Patients (Tentative 8th Edition) [8], Dayuan decoction is recommended in the mild stage; for the moderate cases, Xiaochuhu decoction and Maxingshigan decoction are recommended. In the later stage, Chinese herbal formulas are considered to be administered by injection. All these prescriptions provided pharmacological efficiency on anti-inflammatory, antiviral, antipyretic, expectorant, antiasthmatic, and antitussive, with multiple targets that may act by modulating the patient’s immune response, directly fighting the pathogen, and improving the patient’s symptoms [9]. All these factors indicate that Chinese herbal medicine (CHM) has superior advantages in the current COVID-19 pandemic as adjunctive therapy combined with conventional Western monotherapy (CWM) [10].

At present, a considerable meta-analysis has indicated that CHM plus Western medicine treatment of COVID-19 can reduce the exacerbation rate of pneumonia and shorten the antipyretic time [11–13]. However, these studies did not restrict enrollment, probably because most early trials were not specifically divided subjects according to clinical syndrome types. There were specific differences in the treatment and prognosis of COVID-19 patients at different stages. This meta-analysis, rigorous and objective, discusses a series of existing randomized controlled trials (RCTs), prospective cohort studies (PCSs), and retrospective cohort studies (RCSs) comparing ICW therapy with CWM in distinct stages of COVID-19 patients, evaluating the effectiveness of ICW therapy, and summarizing commonly used ingredients from prescriptions included, figuring out the most promising candidates, which may aid in the development of facilitating conventional treatment.

## Materials and methods

This study was performed according to the Preferred Reporting Items for Systematic Review, Meta-Analysis (PRISMA) statement and the statement by the Meta-analysis of Observational Studies in Epidemiology (MOOSE) group [14, 15].

### Search strategy

A structured and comprehensive search was performed in PubMed, Embase, Cochrane Library, Wan Fang Database, VIP Information Database, SinoMed, and China National Knowledge Infrastructure (CNKI) until May 20, 2024. The literature search strategy was developed using the patient characteristics, intervention, comparison, and outcomes framework [16]. (S1 Table for detailed search strategy of English databases and S2 Table for Chinese databases).

### Eligibility criteria

#### Types of studies

Published randomized controlled trials (RCTs), prospective cohort studies (PCSs), or retrospective cohort studies (RCSs) regarding the comparison between ICW with CWM therapy on COVID-19 patients were included in this meta-analysis.

#### Stages of participants

Participants were patients with a precise diagnosis of COVID-19, aged≥18 years. According to the clinical manifestations, the patients with COVID-19 were divided into four stages [17]: 1) Mild stage, with mild clinical symptoms and no pneumonia in imaging; 2) Moderate stage, patients mainly had symptoms of fever and respiratory symptoms with imaging manifestations of pneumonia; 3) Severe stage, adults who met any of the following criteria: respiratory rate≥30 breaths/min; resting state oxygen saturation≤93%; arterial partial pressure of oxygen (PaO2)/oxygen concentration (FiO2)≤300 mmHg. Patients with more than 50% of lesions progressing within 24 to 48 hours on lung imaging should be treated as severe cases; 4) Critical stage, meeting any of the following conditions: respiratory failure requiring mechanical ventilation; the presence of shock; other organ failure requiring ICU monitoring and treatment.

In this meta-analysis, patients at distinct stages were divided into the mild/moderate group or severe/critical group according to the clinical classification of COVID-19, and enrolled patients in each study should more than 40.

#### Methods of interventions

Patients in the ICW group were treated by the combination of Chinese herbal medicine and conventional Western medicine therapy, and the conventional Western monotherapy group (CWM) was only treated by Western medicine therapy.

CWM therapy included general treatment, antiviral medicines, antibacterial medicines, oral corticosteroids, nutrition therapy, supplemental oxygen, and mechanical ventilation.

CHM treatment contains Chinese medicine such as decoction, granule, capsule, liquid, and injection.

#### Types of outcome measures

The outcome indicators of this meta-analysis are based on an evidence-based clinical practice guideline [2], consensus [8], and present meta-analysis [11–13].

**Primary endpoints**

- Improvement rate of chest CT.
- Severe conversion rate of mild/moderate patients.
- Mortality rate of severe/critical patients.
- Negativity time of nucleic acid.

**Secondary endpoints**

- Clinical improvement rate and time of main symptoms (fever, cough, breathlessness, fatigue).
- Length of hospital stays.
- Inflammatory markers included the level of white blood cells (WBC), neutrophils (NEU), lymphocytes (LYM), and C-reactive protein (CRP) on the change from baseline.

#### Exclusion criteria

- Non-CHM interventions such as natural medicines, acupuncture, or moxibustion.
- Articles with inconsistent outcome indicators and incomplete index data.
- Reviews, case reports, Master/PhD thesis, and unpublished clinical trials.

### Data extraction

Data were extracted from included studies, and the characteristics of study design, course of the treatment, characteristics of included patients (clinical type, comorbidity), treatment regimen of experiment/control, and outcome measures were documented. Study screening and data extraction were conducted independently by two reviewers (Gu and Xiao), and any inconsistencies were settled by the protocol of the Complex meta-analysis Data Extraction Recommendations [18].

### Risk of bias, evidence profile, and heterogeneity assessment

Bias risk was assessed and verified by two authors (Gu and Xiao) using the Cochrane Collaborative Bias Risk Tool for RCTs and Newcastle-Ottawa (NOS) for cohort studies (CSs) [19, 20]. The quality of the evidence for the primary endpoints was assessed according to the Grading of Recommendations Assessment, Development, and Evaluation (GRADE) system [21]. The level of evidence for each outcome was classified by risk of bias, imprecision, inconsistency, indirectness, and publication bias. Disagreements were settled through discussion. We used the *I*^2^ statistic to measure heterogeneity between the trials in each analysis; *I*^2^ <50% was an acceptable level of insignificant heterogeneity.

### Data analysis

A pairwise meta-analysis was performed using the statistical platform R 4.1.2. Difference in means (MD) with 95% confidence interval (CI) was utilized for data measurement of continuous outcomes, while ratio risk (RR) with 95%CI for dichotomous data. For data synthesis, we used random or fixed effect models with or without significant heterogeneity (*I*^2^ > 50% was apparent heterogeneity). We conducted sensitivity analyses by excluding each study and changing statistical models to assess whether the results changed significantly due to individual studies. Subgroup analysis was also performed based on various clinical and methodological factors. *P* < 0.05 was considered statistically significant.

## Results

### Study characteristics

**Fig 1:**
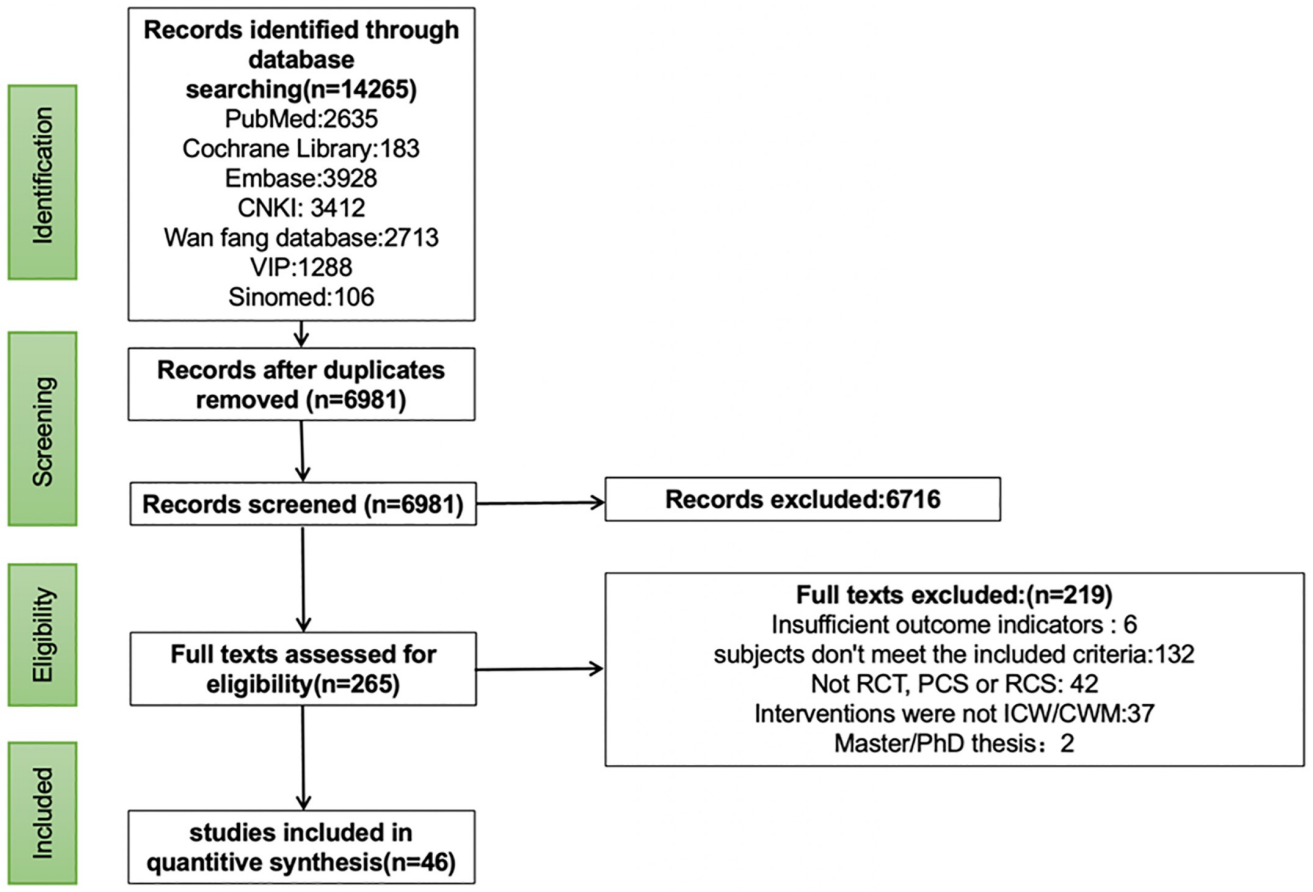
Flow diagram for the identification of studies included.

A structured and comprehensive search was performed in 7 databases up to May 20, 2024. 14265 relevant literatures were retrieved, after moving duplicates (n = 7284), 6981 literatures were screened based on title and abstract, then 265 studies were qualified for full-text review. 46 studies were included in the meta-analysis; 6 articles were excluded due to insufficient data; 132 articles enrolled patients who did not have a clear definition of the type of COVID-19 or the sample size was less than 40; 42 articles studies were not RCT, PCS or RCS; the intervention of 37 articles was not ICW and CWM; 2 articles were Master/PhD thesis. The reasons for excluded studies at the full-reading stage were described in S3 Table. 50 trials in 46 articles with 10492 patients were finally included for analysis (ICW, 63.1%; CWM, 36.9%) [22–66]. Our study contained 24 RCTs [25, 26, 28–30, 36–38, 44, 46, 48–58, 61, 65, 66], 2 PCSs [40, 47] and 20 RCSs [22–24, 27, 31–35, 39, 41–43, 45, 59, 60, 62–64, 67], 7 studies [22, 28, 29, 39, 48, 52, 56] were multicentral and the rest 38 were single-central. 2 studies [41, 53]conducted three-armed trials, and another 2 studies [39, 45] contained two double-arm trials. 8 studies [46, 48, 51, 52, 55–57, 65] were open-label. The flow diagram of the study’s screening regarding the PRISMA 2020 statement is summarized in Fig 1. Among eligible patients, the ratio of mild/moderate COVID-19 was 83%, and severe/critical COVID-19 was 17%. The relevant information is described in S4 Table.

### Risk of bias

8 studies [46, 48, 51, 52, 55–57, 65] were open-label trials in which patients and assessors were aware of the study design, and 10 studies [25, 26, 28–30, 36–38, 44, 53]didn’t describe the performance of allocation concealment (S1 Fig), both of them may confuse the subjective outcomes measurements. 14 RCSs [22, 27, 31–33, 39, 41–43, 45, 59, 60, 62, 63] did not have a detailed description of the target disease history, and all RCSs except Yuanyuan Wang et al. [64] matching patients in a 1:1 ratio using propensity score, controlled only by the main confounding factors. Of note, the experimental group and control group with no response rates were different in trials conducted by Chao Qun Huang, et al. [39], and we deduct points accordingly (S5 Table).

### Description of CHM prescription

The frequency of each Chinese herb in recipes and the recommend prescriptions at each stage were summarized (S2 Fig). Among all ingredients documented in this study, Semen Armeniacae Amarum (Kuxingren) (2.43%) combined with Herba Ephedrae (Mahuang) (2.43%) were the top-ranked Chinese herb pair and Radix Glycyrrhizae (Gancao) (6.25%) was the top-ranked single Chinese herb among COVID-19 ranging from mild to severe cases. Fructus Forsythiae Suspensae (Lianqiao) (2.39%) plus Flos Lonicerae (Jinyinhua) (2.39%) were frequently recommended for mild/moderate stage. While for severe/ critical COVID-19, the most widely used Chinese herb pairs were Radix Paeoniae Rubra (Chishao) (6.33%) plus Szechuan Lovage Rhizome (Chuanxiong) (2.53%).

Five dosage formulations of CHM were included, including decoction (43.4%), granule (30.2%), capsule (7.5%), injection (7.5%), and tablet (3.8%). The most widely used dosage formulations for the early stage were decoction and granule, followed by capsule and tablet. When it came to a later stage, injection was commonly recommended. Further, twice daily (56.7%) and thrice daily (36.7%) were the most frequently usage of CHM. The details of CHM prescriptions are summarized in S6 Table.

### Primary endpoints

#### Improvement rate of chest CT

**Fig 2:**
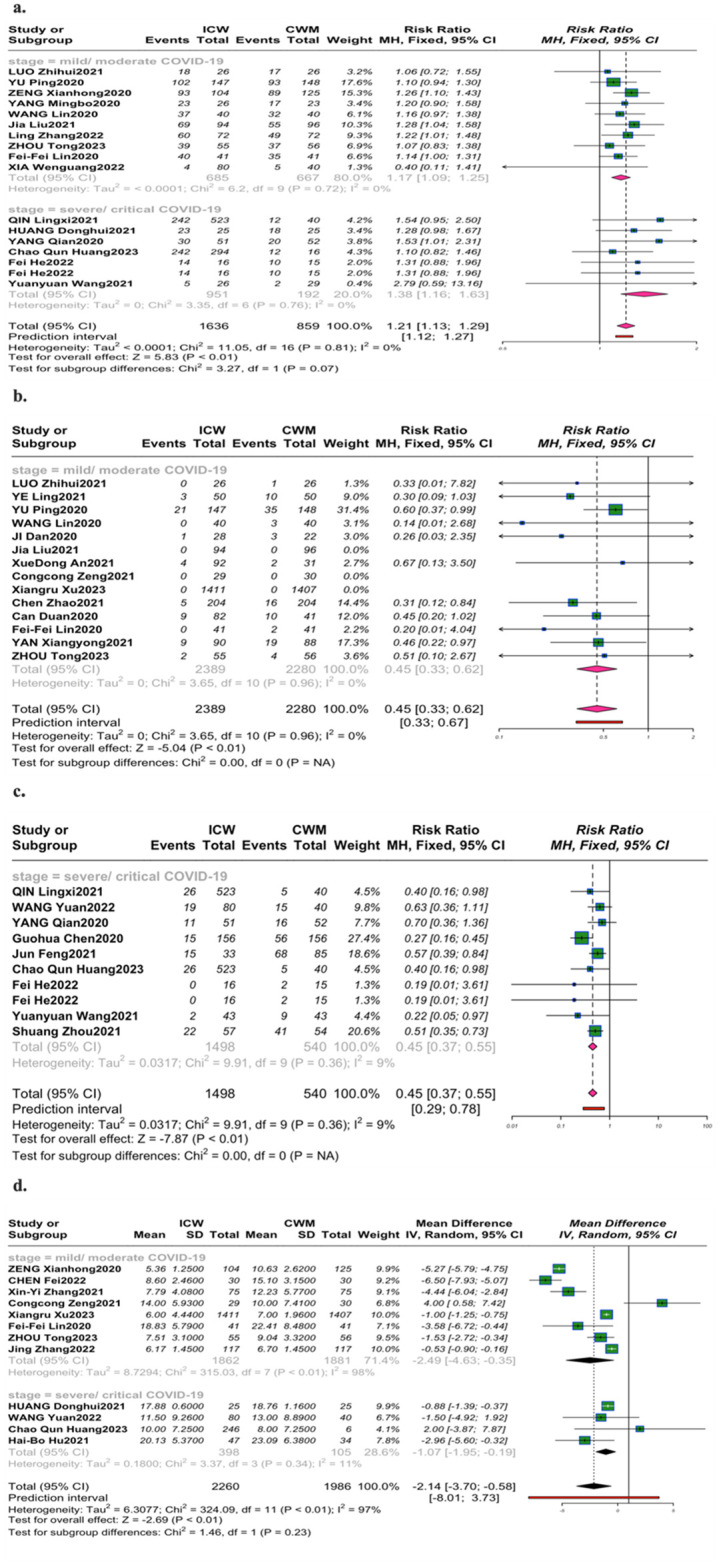
Forest plot of the improvement in primary endpoints. (a). Improvement rate of chest CT (b). Severe conversion rate of mild/moderate patients (c). Mortality rate of severe/critical patients (d). Negativity time of nucleic acid.

The recovery rate of chest CT manifestations was reported in 17 trials [22–24, 26–29, 32, 38, 39, 41, 44, 46, 56, 61, 64]. Overall, the ICW group (72.9%,95%CI [0.59; 0.85]) was associated with fewer chest radiograph abnormalities in all stages of COVID-19(RR = 1.21, 95%CI [1.13,1.29]; *P*<0.01; *I*^2^ = 0%) and proved to be remarkably superior to the CWM therapy (58.3%,95%CI [0.46; 0.7]) (Fig 2a). Additionally, the pooled recovery rate of chest CT with added CHM was 75.4% (95%CI [0.56,0.9]) in the mild/moderate patients versus 69.1% (95%CI [0.47,0.87]) in the severe/critical cases.

#### Severe conversion rate of mild/moderate patients

Severe conversion rate revealed in 14 trials [24–26, 29, 33, 37, 38, 40, 44, 46, 50, 51, 55, 57], CHM add-on effectively prevented disease progressed into severe/critical status (RR = 0.45, 95% CI [0.33,0.62]; *P*< 0.01; *I*^2^ = 0%) (Fig 2b). 2% of cases reported disease progression (95%CI [0.01,0.05]) in the ICW group, less than 7% in the CWM group (95%CI [0.03,0.13]).

#### Mortality rate of severe/critical patients

Significant reduction of mortality with severe/critical COVID-19 was revealed in 10 trials [22, 30, 32, 39, 41, 52, 62, 63, 64] (RR = 0.45, 95% CI [0.37,0.55]; *P*< 0.01; *I*^2^ = 9%) (Fig 2c). The pooled mortality was 12% (95%CI [0.04,0.23]) in the ICW group versus 33% in the CWM group (95%CI [0.18,0.5]).

#### Negativity time of nucleic acid

12 trials [23, 27, 30, 31, 38, 39, 44, 48, 51, 55, 65, 67] evaluated the negativity time of nucleic acid (Fig 2d) (MD = -2.14,95% CI [-3.70, -0.58]; *P*< 0.01; *I*^2^ = 97%). Compared to CWM therapy (11.11 days, 95%CI [8.1;14.11]), both mild/ moderate COVID-19 and severe/ critical COVID-19 treated with ICW combination (l2.95 days, 95%CI [9.73; 16.17]) demonstrated the statistical differences in shortening time to negative conversion (MD = -2.49, 95% CI [-4.63, -0.35]; *P* < 0.01; *I*^2^ = 98%), (MD = -1.07, 95% CI [-1.95, -0.19]; *P*< 0.01; *I*^2^ = 11%). Additionally, the pooled mean negativity time of nucleic acid with added CHM was 9.21 days (95%CI [6,12.42]) in the mild/ moderate patients versus 14.89 days (95%CI [10.1,19.69]) in the severe/critical cases.

### Secondary endpoints

#### Rate of fever reduction, time to reduction of fever

**Fig 3:**
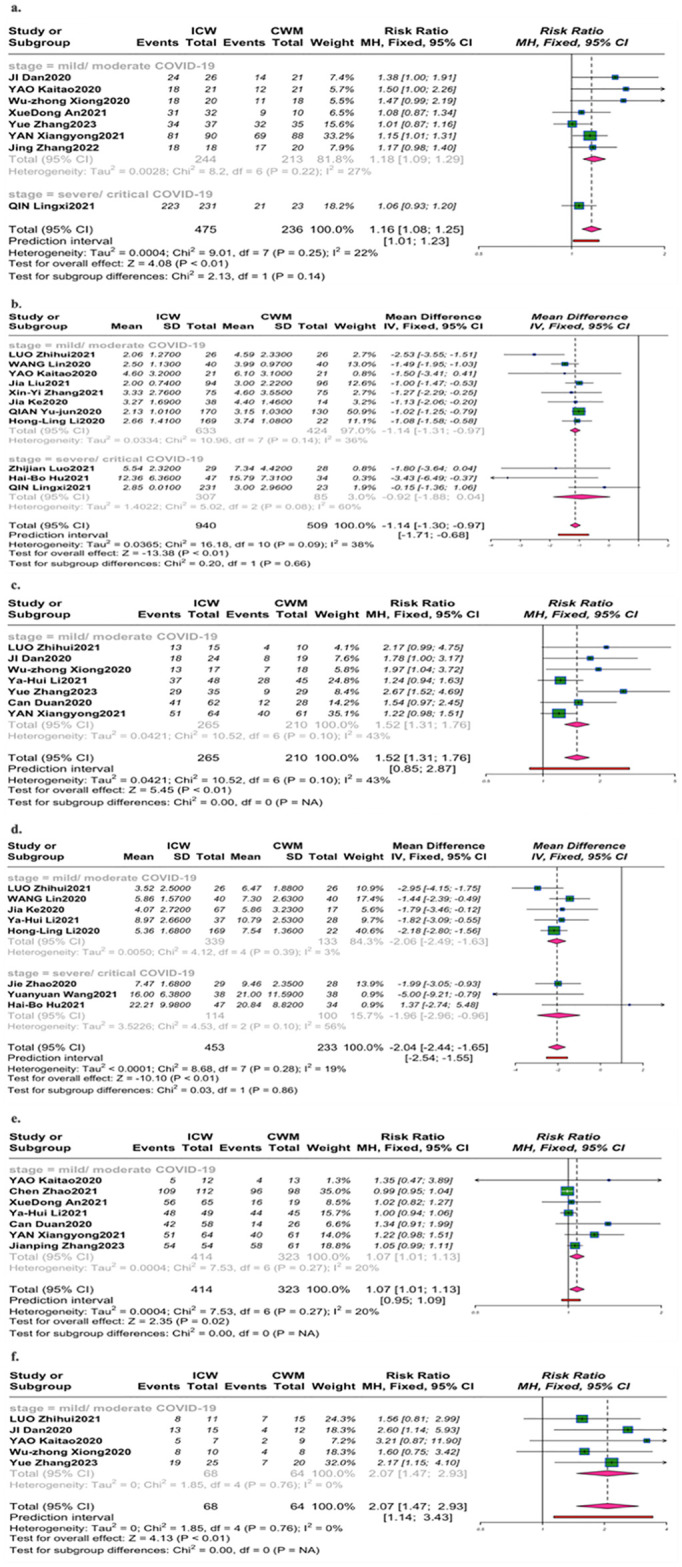
Forest plot of the second endpoint of the clinical symptom improvement. (a). Rate of fever reduction; (b). Time to the reduction of fever; (c). Improvement rate of cough; (d). Time to improvement of cough; (e). Improvement rate of breathless; (f). Improvement rate of fatigue.

No statistical differences were observed in the rate of fever reduction from 1 trial [22] (Fig 3a) for severe/critical COVID-19 (RR = 1.06, 95% CI [0.93,1.20]; *P* = 0.4), while for mild/moderate cases in 7 trials [33, 34, 36, 40, 49, 50, 65], ICW therapy illustrated significantly clinical difference, compared with CWM therapy (RR = 1.18, 95%CI [1.09, 1.29]; *P*< 0.01; *I*^2^ = 27%). A similar trend was found in time to the reduction of fever, ICW therapy led marginal differences for severe/ critical COVID-19 in 3 trials [22, 58, 67] (MD = -0.92, 95% CI [-1.88,0.04]; *P* = 0.06, *I*^2^ = 60%), while more mild/ moderate COVID-19 participants in 8 trials [24, 29, 34, 42, 43, 46, 48, 59] received added CHM had a clinically meaningful benefit (Fig 3b) (MD = -1.14, 95% CI [-1.31, -0.97]; *P*< 0.01; *I*^2^ = 36%).

#### Improvement rate of cough, time to improvement of cough

Improvement rate of cough was reported only with mild/ moderate COVID-19 (Fig 3c). Compared with the CWM group, this review showed a significant improvement in the number of cough reduction cases by added CHM in 7 trials [24, 33, 35, 36, 37, 40, 49] (RR = 1.52,95% CI [1.31,1.76]; *P* <0.01; *I*^2^ = 43%). According to the recovery time of cough (Fig 3d), different trends were observed at early stage of COVID-19 in 5 trials [24, 29, 35, 43, 59] (MD = -2.06, 95% CI [-2.49, -1.63]; *P*<0.01; *I*^2^ = 3%), and the later stage in 3 trials [54, 64, 67] (MD = -1.96, 95% CI [-2.96, -0.96]; P = 0.05; *I*^2^ = 56%).

#### Improvement rate of fatigue, improvement rate of breathlessness

Regarding the symptom of fatigue or breathless (Fig 3e and 3f), a significant improvement on number of fatigue and breathless reduction cases by ICW was documented in 7 studies [34, 35, 37, 40, 50, 57, 66] /5 studies [24, 33, 34, 36, 49], at early stage of COVID-19(RR = 1.07, 95% CI [1.01,1.13]; *P*<0.01; *I*^2^ = 20%), (RR = 2.07, 95% CI [1.47,2.93]; *P*<0.01; *I*^2^ = 0%).

#### Improvement rate of symptom, time to improvement of symptom

According to the recovery rate of clinical symptoms (fever, cough, fatigue, breathlessness), compared with later stage, mild/moderate cases proved to be more effective by adding CHM (RR = 1.06, 95%CI [0.93,1.20]; RR = 1.23, 95%CI [1.17,1.29], respectively). Likewise, the time to the reduction of clinical symptoms (fever, cough) showed the same trend (MD = -1.42,95% CI [-2.11, -0.73]; MD = -1.26,95% CI [-1.42, -1.11], respectively). Additionally, the pooled mean recovery time to symptom with adding CHM was 3.85 days (95%CI [2.78,4.92]) in the mild/moderate patients versus 11 days (95%CI [5.24,16.72]) in the severe/critical cases. For distinct stages of COVID-19, the pooled recovery rate was 65% (95%CI [0.55; 0.75]) with western medicine monotherapy versus 86.7% (95%CI [0.81; 0.91]) with added CHM. Likewise, the pooled mean time to recovery was 7.69 days (95%CI [5.31,10.08]) in CWM versus 6.06 days (95%CI [3.72,8.39]) with adding CHM.

#### White blood cell count

**Fig 4:**
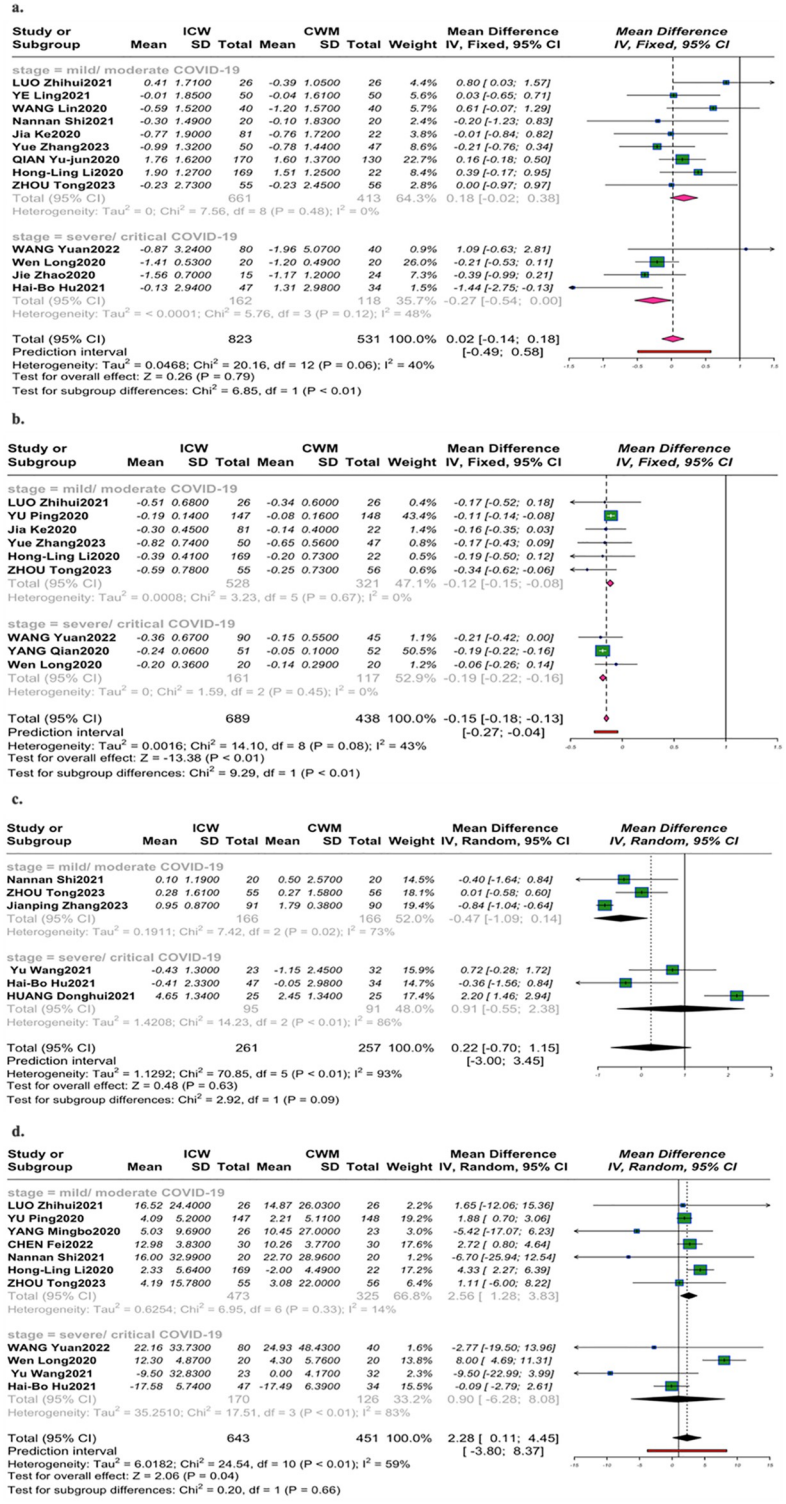
Forest plot of inflammatory markers. (a). White blood cell count; (b). Lymphocyte count; (c). Neutrophils count; (d). C-reactive protein level.

13 studies [24, 25, 29, 30, 36, 42–44, 47, 53, 54, 59, 67] (Fig 4a) evaluated level of WBC(×10^9^L), no statistical difference was observed in both groups of COVID-19 (MD = 0.18, 95%CI [-0.02, 0.38]; *P* = 0.08; *I*^2^ = 0%), (MD = -0.27, 95%CI [-0.54, 0.00]; *P* = 0.05; *I*^2^ = 48%).

#### Lymphocyte count

For the level of LYM(×10^9^L) in 9 trials [24, 26, 30, 32, 36, 59, 43, 44, 53], a similar trend was found in distinct stages of COVID-19(Fig 4b), ICW increased the level of LYM, compared with monotherapy. (MD = -0.12, 95%CI [-0.15, -0.08]; *P* <0.01; *I*^2^ = 0%), (MD = -0.19,95%CI [-0.22, -0.16]; *P* <0.01; *I*^2^ = 0%).

#### Neutrophils count

Effects of ICW on the level of NEU(×10^9^L) were measured in 6 trials [23, 44, 47, 60, 66, 67], showing no trend over influencing the level of NEU with mild/ moderate COVID-19(MD = -0.47, 95%CI [-1.09,0.14]; *P* = 0.13; *I*^2^ = 73%), and severe/critical COVID-19(MD = 0.91, 95%CI [-0.55,2.38]; *P* = 0.22, *I*^2^ = 86%) (Fig 4c).

#### C-reactive protein

The CRP (mg/L) level was documented in 11 trials [24, 26, 28, 30, 31, 43, 44, 47, 53, 60, 67]. A different trend was found in distinct stages of COVID-19(Fig 4d). The level of CRP was significantly reduced by adding CHM in the early stage of COVID-19(MD = 2.56, 95%CI [1.28,3.83]; *P* <0.01; *I*^2^ = 14%), while no statistical differences were identified at the later stage (MD = 0.90, 95%CI [-6.28, 8.08]; *P* = 0.81; *I*^2^ = 83%).

#### Length of hospital stays

**Fig 5:**
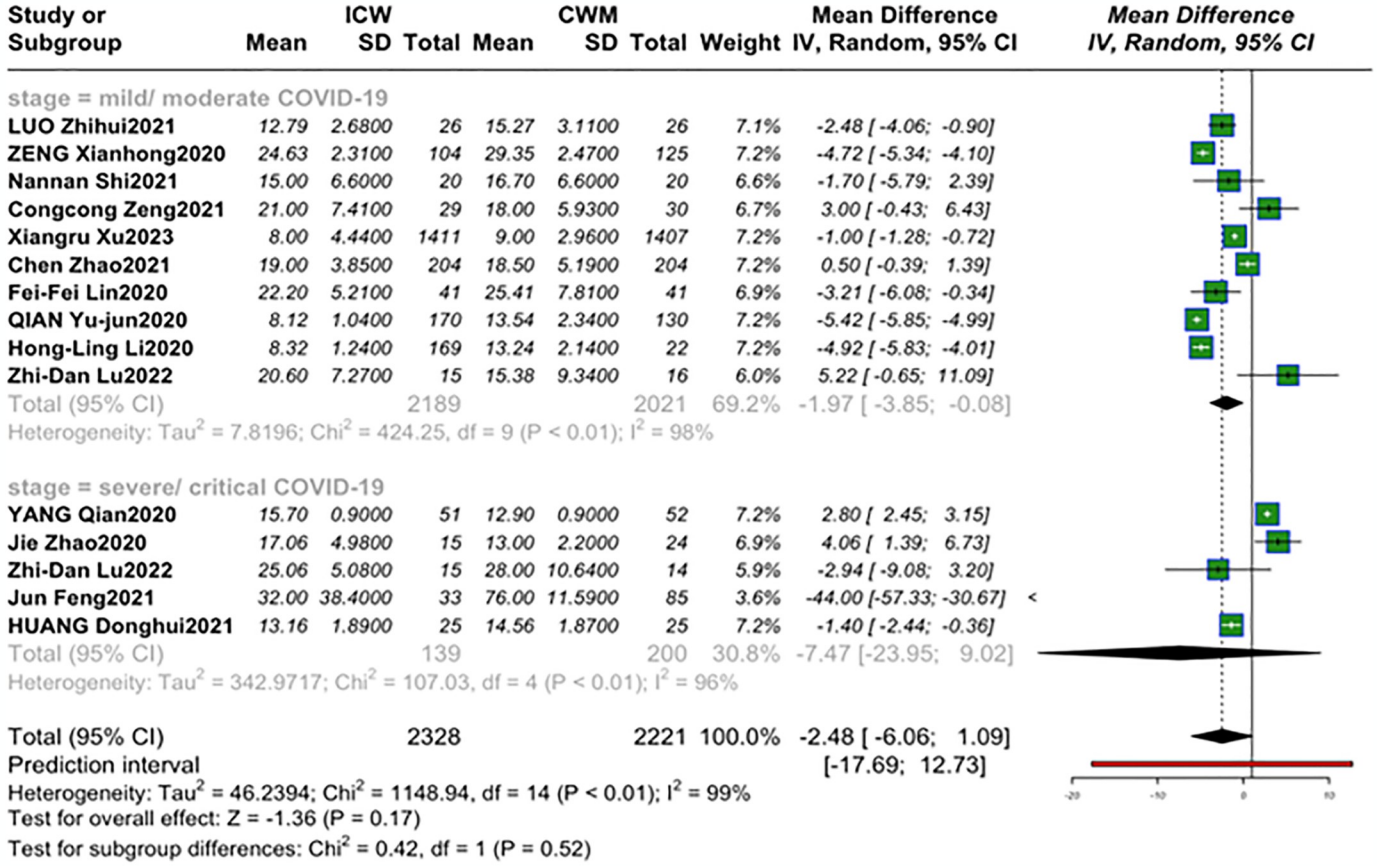
Forest plot of the hospital stay length in distinct stages of patients.

Fifteen studies evaluated hospitalizations (Fig 5), different trend was observed compared to CWM therapy at distinct stages of COVID-19(MD = -1.97, 95% CI [-3.85, -0.08]; *P* = 0.04; *I*^2^ = 98%), (MD = -7.47, 95% CI [-23.95,9.02]; *P* = 0.37; *I*^2^ = 96%) [23, 24, 27, 32, 38, 42, 43, 45, 47, 51, 54, 55, 57, 63]. Compared with the CWM group (21.23 days,95%CI [13.02; 29.45]), patients who received ICW therapy (16.9 days,95%CI [13.73;20.08]) had a shorter length of hospital stays. Further, the pooled mean length of hospital stays of mild/moderate patients treated with ICW therapy was 15.9 days (95%CI [11.92; 19.89]) days, which was shorter than 19.1 days (95%CI [13.66; 24.49]) in severe/critical patients.

### Sensitivity analysis

Sensitivity analyses were conducted to investigate sources of heterogeneity by excluding each study; there was no significant change in effects during this exercise, indicating that no individual study had undue influence on the results.

### Subgroup analysis

The negativity time of nucleic acid (*I*^2^ = 97%), neutrophils count (*I*^2^ = 93%), C-reactive protein count (*I*^2^ = 59%), and length of hospital stays (*I*^2^ = 99%) meta-analysis exhibited considerable heterogeneity. Selected subgroup analysis resulted in a reduction of heterogeneity (Table 1). The results suggested that study course and distinct stages of COVID-19 may be the source of heterogeneity for negativity time of nucleic acid; sex(male%) may cause the heterogeneity for the level of neutrophils count; dosage formulations and usage may be the source of heterogeneity for the level of C-reactive protein count; and age group may be the cause of heterogeneity for the length of hospital stays. Moreover, subgroup and sensitivity analyses for the other primary outcomes were documented in S7 Table. There was a significant trend towards an older group who exhibited less effectiveness by added CHM in negativity time of nucleic acid, the improvement rate of cough, level of NEU, and CRP (*P*_subgroup_<0.01, *P*_subgroup_ <0.01, *P*_subgroup_ = 0.02, respectively). Stratifying studies by design did not produce statistically significant differences in effect sizes (*P*_subgroup_ >0.05; each outcome).

**Table 1:** Subgroup and sensitivity analyses for the primary outcomes.

		No. of Studies	MD/RR, 95%CI	*I* ^2^	Q	*P*_subgroup_
**Negativity time of nucleic acid, mean ± SD**						
**Age Group** ^a^	≤50years	5	-4.04(-6.18,-1.9)	98.40%	7.61	0.006*
	>50years	7	0.49(-1.91,2.9)	93.10%		
**Study Design**	RCT	6	-0.86(-1.33,-0.38)	69.10%	0.54	0.46
	RCS	7	-2.05(-5.17,1.08)	98.10%		
**Dosage Formulations**	decoction/capsule/granule/injection	1	-1.5(-4.9,1.9)	NA	25.86	< 0.0001*
	decoction	7	-0.92(-4.39,2.55)	98.20%		
	injection	1	-4.44(-6.04,-2.84)	NA		
	capsule	1	-0.53(-0.9,-0.16)	NA		
	liquid	2	-1.02(-5.6,-3.23)	NA		
	granule	1	-2.96(-5.6,-0.32)	NA		
**Usage**	once daily	1	-4.44(-6.04,-2.84)	NA	18.07	0.0004*
	2 times daily	7	-0.32(-3.55,2.91)	96.10%		
	3 times daily	1	-1.53(-2.72,-0.34)	NA		
	4 times daily	1	-1(-1.25,-0.75)	NA		
**Study Course**	<14d	3	-0.86(-1.29,-0.44)	63.00%	0.04	0.84
	≥14d	3	-0.39(-4.82,4.04)	81.30%		
**Sex(male%)**	<50%	6	-1.01(-4.1,2.07)	95.30%	0.17	0.68
	≥50%	7	-1.84(-4.26,0.58)	98.00%		
**Stage of COVID-19**	mild/moderate COVID-19	8	-2.49(-4.63,-0.35)	98.00%	1.46	0.23
	severe/critical COVID-19	4	-1.07(-1.95,-0.19)	11%		
**Neutrophils, mean ± SD**						
**Age Group** ^a^	≤50years	1	-0.84(-1,-0.64)	NA	6.73	0.001*
	>50years	5	0.49(-0.5,1.47)	85.00%		
**Study Design**	RCT	2	-0.46(-1.29,0.36)	86.00%	2.7	0.26
	PCS	1	-0.40(-1.64,0.84)	NA		
	RCS	3	0.91(-0.55,2.38)	86.00%		
**Dosage Formulations**	granule/injection	1	0.72(-0.28,1.72)	NA	17.11	0.002*
	decoction/tablet	1	-0.84(-1.04,-0.64)	NA		
	decoction	2	0.95(-1.6,3.5)	91.90%		
	granule	1	-0.36(-1.56,0.84)	NA		
	liquid	1	0.01(-0.58,0.6)	NA		
**Usage**	2 times daily	2	0.95(-1.6,3.5)	91.90%	1.07	0.3
	3 times daily	2	-0.46(-1.29,0.36)	85.90%		
**Study Course**	<14d	2	-0.46(-1.28,0.36)	85.90%	0.01	0.93
	≥14d	1	-0.4(-1.64,0.84)	NA		
**Sex(male%)**	<50%	3	-0.47(-1.08,0.15)	73.40%	2.8	0.09
	≥50%	3	0.9(-0.58,2.39)	85.80%		
**Stage of COVID-19**	mild/moderate COVID-19	3	-0.47(-1.09,0.14)	73%	2.92	0.09
	severe/critical COVID-19	3	0.91(-0.55,2.38)	86%		
**C-reactive protein, mean ± SD**						
**Age Group** ^a^	≤50years	6	3.53(1.26,5.81)	68.60%	5.4	0.02*
	>50years	5	-0.42(-2.86,2.02)	0%		
**Study Design**	RCT	5	2.60(-1.70,6.91)	71%	0.86	0.65
	PCS	1	-6.70(-25.94,12.54)	NA		
	RCS	5	2.06(-0.36,4.49)	58%		
**Dosage Formulations**	decoction/capsule/granule/injection	1	-2.77(-19.5,13.96)	NA	17.74	0.003*
	granule/injection	1	-9.5(-23,4)	NA		
	decoction	4	3.39(1.86,4.92)	0.00%		
	granule	2	1.29(-0.49,3.06)	41.70%		
	liquid	2	-0.66(-6.73,5.41)	0.00%		
	injection	1	8(4.69,11.31)	NA		
**Usage**	2 times daily	6	4.26(1.68,6.84)	46.50%	4.42	0.11
	3 times daily	2	1.86(0.7,3.02)	0.00%		
	2~4 times daily	1	-5.42(-17.07,6.23)	NA		
**Study Course**	<14d	4	2.9(-1.61,7.4)	78.00%	1.16	0.28
	≥14d	2	-4.46(-17.09,8.16)	0.00%		
**Sex(male%)**	<50%	5	3.15(0.35,5.96)	78.20%	1.91	0.17
	≥50%	6	-0.99(-6.13,4.16)	16.30%		
**Stage of COVID-19**	mild/moderate COVID-19	7	2.56(1.28,3.83)	14%	0.2	0.66
	severe/critical COVID-19	4	0.90(-6.28,8.08)	83%		
**Length of hospital stays, mean ± SD**						
**Age Group** ^a^	≤50years	4	4.1(-5.27,-2.93)	64.10%	0	0.96
	>50years	9	-3.91(-12.06,4.23)	99.10%		
**Study Design**	RCT	4	-0.30(-2.26,1.66)	83.00%	1.44	0.49
	PCS	1	-1.70(-5.79,2.39)	NA		
	RCS	10	-4.37(-11.34,2.59)	99.00%		
**Dosage Formulations**	capsule/granule/injection	2	1.19(-6.81,9.19)	71.80%	1.33	0.72
	decoction	10	-1.53(-3.72,0.67)	99.20%		
	granule	2	-21.23(-65.83,22.37)	97.70%		
	liquid	1	-1(-1.28,-0.72)	NA		
**Usage**	2 times daily	10	-4.52(-11,1.98)	99.10%	1.12	0.29
	4 times daily	1	-1(-1.28,-0.72)	NA		
**Study Course**	<14d	2	-0.31(-1.78,1.15)	90.00%	0.03	0.86
	≥14d	3	-0.68(-4.45,3.1)	73.70%		
**Sex(male%)**	<50%	5	-3.98(-10.75,2.79)	99.20%	0.42	0.52
	≥50%	10	-3.98(-10.75,2.79)	99.20%		
**Stage of COVID-19**	mild/moderate COVID-19	10	-1.97(-3.85,-0.08)	90%	0.42	0.52
	severe/critical COVID-19	5	-7.47(-23.95,9.02)	96%		

*Statistically significant subgroup effect sizes, ascertained as *P*_subgroup_ (χ2 test) <0.05.

^a^ Studies categorized by age group depending on the mean.

Abbreviation: MD: difference in mean; RR: ratio risk; CI: confidence interval; SD: standard deviation

### Bias and quality of evidence

No publication bias was suggested for the improvement rate of chest CT, which was confirmed by dispersion produced by visual analysis of funnel plots (S3 Fig), and neither was the Egger regression intercept test (*P* = 0.23) statistically significant. The GRADE analysis reported moderate-to-high-quality evidence on the effect of ICW combination versus CWM monotherapy on the primary endpoints. Fifty trials obtained a total of 155 outcome indicators, of which 15 had high-grade scores, accounting for 9.68%; moderate GRADE scores in 102 items, accounting for 65.81%, and 38 (24.5%) low GRADE scores. Details are provided in S8 Table.

## Discussion

Considerable systematic reviews were performed to evaluate the clinical efficiency of adding CHM to the treatment of COVID-19 [13, 67–69]. For uninfected and suspected COVID-19 patients, Siying Hu found CHM could reduce the risk of infection [70]. For confirmed COVID-19 patients (regardless of stage), previous literature indicates that the combination of Chinese and Western medicine treatment has shown significant improvement in overall efficiency, chest CT improvement rate, disease progression rate, as well as fever, malaise, and cough [12, 71]. For COVID-19 patients in the recovery stage, adding CHM could facilitate the improvement of clinical symptoms and lung function and the achievement of a balanced body constitution in these patients recovering from COVID-19 [72]. In line with them, we also found a significantly positive effect that adding Chinese medicine strengthened the therapeutic effect of Western monotherapy in confirmed COVID-19 patients at all distinct stages. Compared with the western monotherapy, the ICW group showed a higher recovery rate of chest CT, shortened negativity time of nucleic acid, reduced number of mild/moderate patients developing into severe conditions, and decreased mortality rate. In contrast to previous meta-analyses that focused only on a certain stage of COVID-19 or did not strictly restrict the enrollment of patient (probably because most early trials did not divide the patients into distinct clinical stages specifically) [12, 70, 71]. In this study, COVID-19 patients were strictly categorized into different stages and the therapeutic effects of herbal medicine on co patients in different stages were explored. For mild/moderate COVID-19 patients, compared with Western medicine monotherapy, the ICW group showed a clinically meaningful difference in improving chest CT by 11%, relieving symptoms by 23%, shortening time to recovery and negative conversion, decreasing the rate of patients developing into severe conditions by 5%. We did not observe the statistical difference in clinical symptom improvements and time to reduction of clinical symptoms in the severe/critical stages. This phenomenon may be in the early stage of COVID-19 when patients manifest fever, cough, breathlessness, and fatigue, which belong to the category of typical external syndrome from the perspective of CHM [73]. With timely intervention of CHM at this stage, COVID-19 disease progression can be effectively blocked from mild to critical stages, thus shortening the course of the disease, improving the cure rate, and preventing deterioration. In the plaque reduction experiment, Merarchi M found that LQHW could inhibit the proliferation of influenza virus, and the remarkable decrease of virus titer occurred mainly in 0–2 hours, suggesting that the early intervention of CHM as adjuvant drugs may inhibit the early replication of influenza virus [74]. However, some symptoms, including fatigue and breathlessness in this stage, were not documented, which required more carefully performed trials to support this evidence further.

Laboratory tests are crucial for precisely predicting the prognosis and therapeutic effect in COVID-19 cases. We typically added laboratory test results as observational indicators, while previous studies only included clinical indicators (11–130). Interestingly, we found that the level of LYM was significantly elevated by ICW therapy, which might suggest that patients in the later stage suffer from severe immune imbalance. Thus, improving the level of immune cells was helpful to improve the prognosis. We also found that although a three-armed trial of 60 severe COVID-19 patients conducted by Wen Long et al. [52], compared with the low dose group of 50 mL XBJ injection, the CRP of the high dose group of 100 mL XBJ injection was significantly decreased, no remarkable superiority was observed according to the level of CRP in this study.

Additionally, due to the limited number of clinical trials, most previous research only focused on the single prescription [67, 68], so we systematically summarized and presented the potential drug candidates of CHM remedy recommended at distinct stages for COVID-19 in this meta-analysis. For mild to moderate COVID-19 cases, Lianhua Qingwen (LHQW) granules, Jinhua Qinggan (JQ) granules, and Qingfei Paidu Decoction (QPD) were most frequently used. In line with the 8thVersion of Diagnosis and Treatment Protocol for COVID-19(8) and previous studies [69, 75, 76], LHQW, a Chinese patent medicine composed of 13 herbs, has been used to treat the early stage of COVID-19. It could significantly inhibit SARS-CoV-2 replication, alter the viral morphology, reduce the cytokine release from host cells, and confer anti-inflammatory activity in vitro [77]. In our study, two studies including the RCT and RCS, provided evidence for the advantage of treating in mild/moderate patients with LHQW(26,34). Also, Fan AY found that JQ was developed for mild cases during the pandemic of COVID-19 [12]. While Qingfei Paidu Decoction (QPD) recommended by the 8th Version of Diagnosis and Treatment Protocol for COVID-19 and the previous reviews (8, 12,72), and was used to treat COVID-19 patients with mild, moderate, severe cases. QPD was also used reasonably with the consideration of the actual conditions of critically ill patients. In this meta-analysis, only one RCS was performed with moderate COVID-19 cases, indicating a better therapeutic effect of QPD than CWM(27). Jian Chen divided QPD into five functional units (FUs) and measured by drug perturbation of COVID-19 network robustness, showing that all five FUs may protect COVID-19 independently, and targeting 8 specifically expressed drug-attacked nodes which were related to the bacterial and viral responses, immune system, signaling transduction, etc [78]. When it comes to severe and critical cases, Shengfu injection, Xuebijing injection, and Tanreqing injection were most frequently used. In accordance with previous meta-analysis [75], Xuebijing injection plus routine treatment could reduce the development of ARDS and the use of mechanical ventilation. Wen-Jiang Zheng [79] used the Swiss Target Prediction database to predict XBJ’s effector targets and mapped them to the abovementioned COVID-19 disease targets, indicating the pharmacological mechanisms of XBJ in the treatment of COVID-19, which could be explained by the effector mechanism of sepsis and severe pneumonia treatment. However, the potential mechanisms of other CHM in this study are still unclear. In the future, more research on clinical evidence and molecular mechanisms by Chinese herbal formulas are warranted.

Besides, most previous studies did not perform the subgroup analysis based on methodology and clinical factors [11–13, 67, 68, 71]. Thus, extensive literature searching of relevant clinical trials published in both Chinese and English databases concerning all-cross recipes was conducted, and up to 46 studies were enrolled in this review, including RCTs and, PCSs and RCSs. The use of a comprehensive search strategy to identify and select studies for review could minimize selection bias, and with sufficient trials to support subgroup analysis on methodology and clinical factors, leading to a more persuasive and feasible conclusion, which is essential for confirming the effectiveness of CHM for COVID-19 from different angles and levels. We also evaluated the effectiveness of adding CHM by clinical heterogeneity, methodological limitations, and subgroup analysis. Our results indicated that course, sex, dosage formulations, usage, and age may cause heterogeneity. There was a significant trend towards that older patients were less effective by adding CHM in negativity time of nucleic acid, the improvement rate of cough, level of NEU, and CRP, probably due to the older age in later stage COVID-19 included in this study.

## Limitation

Firstly, the exact efficacy of long-term symptoms of COVID-19 of CHM deserves continuous attention and medical observation, especially for patients in the critical stage. However, the duration of all the studies is shorter than one month, which may cause the missed. Secondly, we included RCTs, PCSs, and RCSs with different underlying target variables, with relatively small sample size in some RCTs. Although no significant heterogeneity was detected between these three types of the study, the potential selection bias still exists due to the different research types. Besides, the quality of the included studies is not in the same accordance. Issues regarding sequence generation of randomization, concealment of allocation, reporting on blinding, dropouts, and pre-estimation of sample size should not be neglected in the further studies. Thirdly, 16 literatures did not document the exact dose size of single-flavor drugs in each recipe, and 10 literatures used Chinese medicine differentiation treatment according to the signal patient, which caused difficulty in summarizing the dose size of single-flavor drugs in this meta-analysis. Besides, a customized medication may have a better effect than fixed formulas for reaching a better therapeutic effect in COVID-19 patients. However, the dose and composition of formula ingredients may be the source of heterogeneity which may disturbs the final conclusions. Thirdly, our study population is based on the Chinese populations, which may lead to the bias from ethnicity. So, we typically limited our conclusions and recommendations on Chinese COVID-19 patients. Moreover, although we conducted subgroup analysis according to diversity in CHM syndrome differentiation treatment and standard Western medicine treatment in each study, figuring out the source of heterogeneity, more trials developed strictly according to the CONSORT statement [80] for CHM were necessary to confirm the conclusion. Finally, the number of included studies focused on severe/critical COVID-19 was still relatively small, and only one study reported a rate of fever reduction, which may lead to the possibility that the efficacy was not statistically significant. More large-scale clinical trials, which are performed at later stages, are needed in the future to obtain unbiased results.

## Conclusions

ICW had a significantly better effect than Western medicine monotherapy in all distinct stages of Chinese COVID-19 patients. Adding CHM could decrease chest radiograph abnormalities, alleviate clinical symptoms, promote rehabilitation, reduce disease progression, and prolong the therapeutic window for Chinese COVID-19 patients. Besides, earlier intervention of CHM may contribute to a better therapeutic effect in Chinese populations. However, considering the intrinsic heterogeneity of CHM and some qualities of included studies were poor, which may affect conclusions of the meta-analysis. In the future, more large-scale clinical trials, which are performed with fixed formulas, are needed for obtaining unbiased results.

## Supporting information

S1 Fig Risk of bias assessment. (DOCX)

S2 Fig The recommended Chinese herds/prescriptions for different clinical types of COVID-19. (DOCX)

S3 Fig Funnel plot of improvement rate of chest CT. (DOCX)

S1 Table Details of the search strategy of PubMed/ Embase/ Cochrane Library. (DOCX)

S2 Table Details of the search strategy of Wan fang Database / VIP Information Database/ SinoMed/ China National Knowledge Infrastructure. (DOCX)

S3 Table The reason for excluded studies at full-reading stage. (DOCX)

S4 Table General information of included studies. (DOCX)

S5 Table Bias risk assessment results of included retrospective studies. (DOCX)

S6 Table Components of TCM prescriptions in the included studies. (DOCX)

S7 Table Subgroup and sensitivity analyses for the other main outcomes. (DOCX)

S8 Table Quality evaluation of outcome indicators. (DOCX)

S1 Checklist PRISMA checklist COVID-19. (DOCX)
