# Supplementary figures and images for: Effect of Chinese herbal medicine (CHM) as an adjunctive therapy in distinct stages of patients with COVID-19: A systematic review and meta-analysis
Source: PLoS One. 2025 Feb 13;20(2):e0318892. doi: 10.1371/journal.pone.0318892 (PMC11825027; doi:10.1371/journal.pone.0318892)

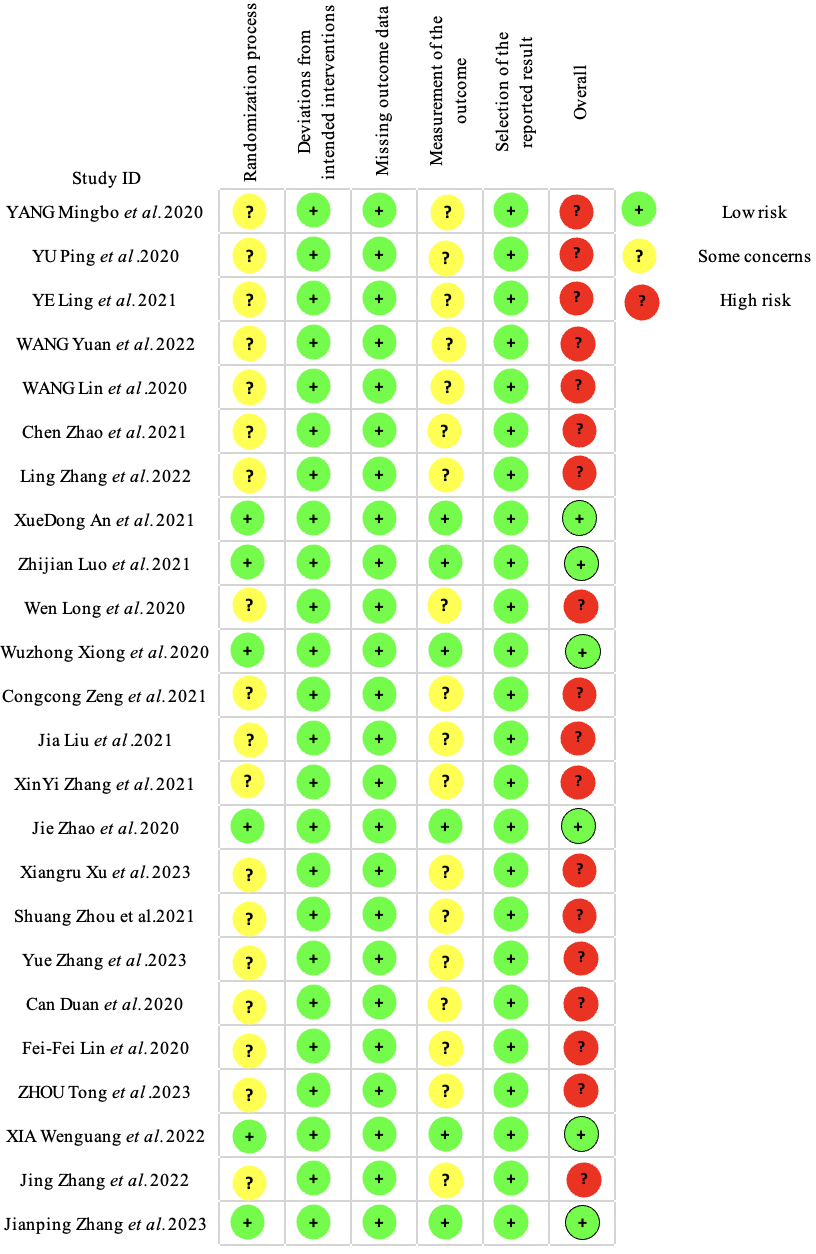


**Supplementary Fig S1. Risk of bias assessment**

Supplement: S1 Fig — (DOCX) [file pone.0318892.s001.docx]

**
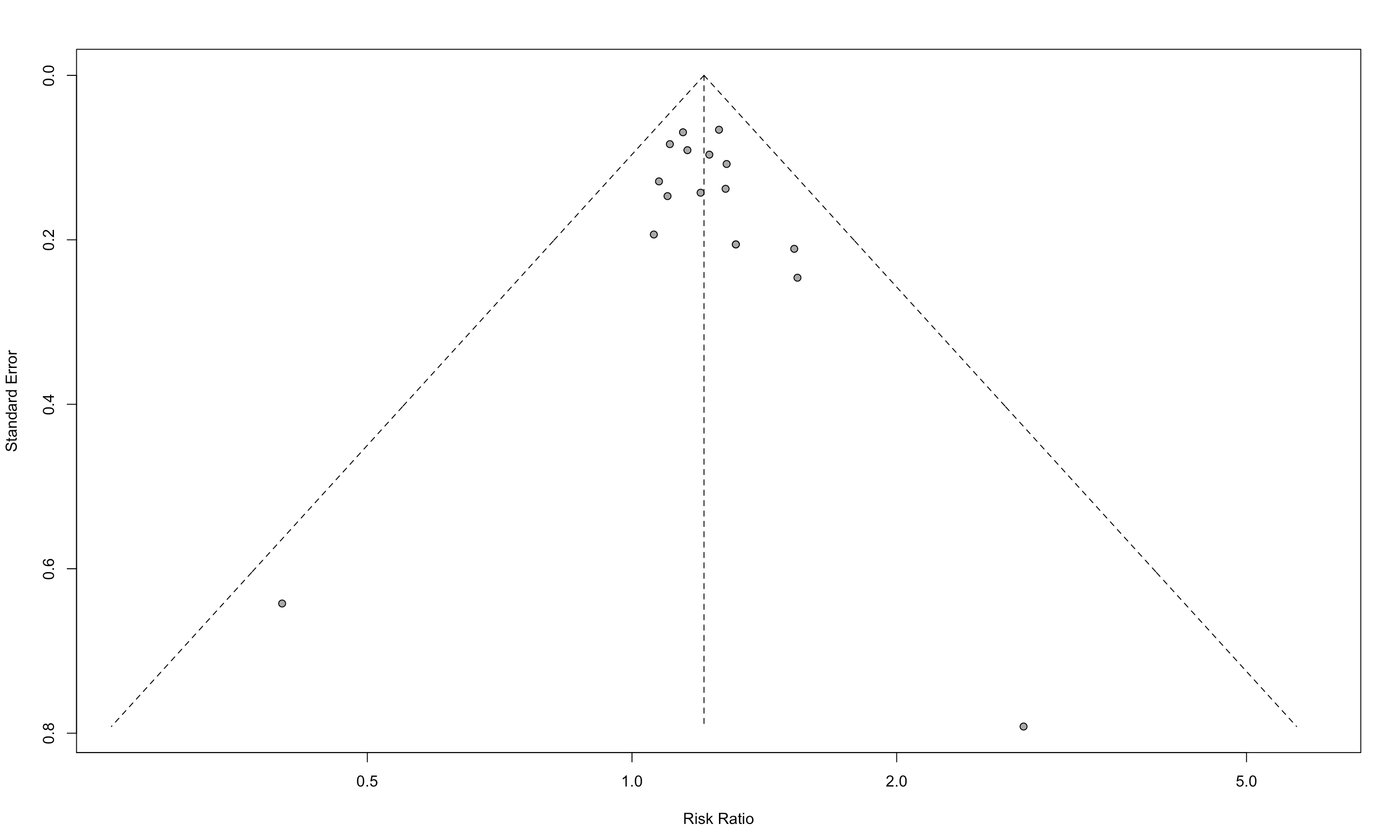
**

**Supplementary Fig S3. Funnel plot of improvement rate of chest CT**

Supplement: S3 Fig — (DOCX) [file pone.0318892.s003.docx]
