# Supplementary material for: Effect of Chinese herbal medicine (CHM) as an adjunctive therapy in distinct stages of patients with COVID-19: A systematic review and meta-analysis
Source: PLoS One. 2025 Feb 13;20(2):e0318892. doi: 10.1371/journal.pone.0318892 (PMC11825027; doi:10.1371/journal.pone.0318892)
Supplement: S2 Fig — (DOCX) [file pone.0318892.s002.docx]

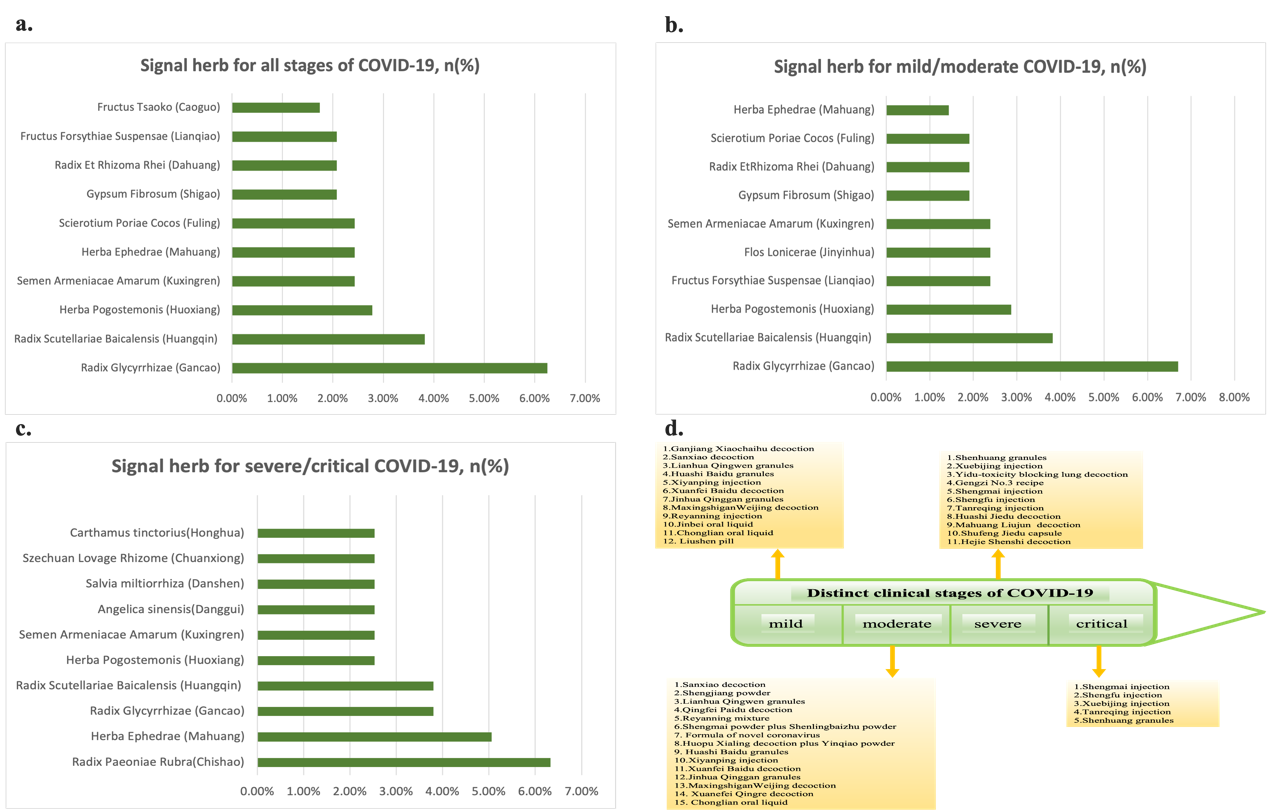


**Supplementary Fig S2. The recommended Chinese herds/prescriptions for different clinical types of COVID-19. Frequency of signal herb for mild/moderate COVID-19 (a), severe/critical COVID-19 (b), and all types of COVID-19 (c). Chinese prescriptions for distinct stages of COVID-19(d).**
