# Supplementary material for: Effect of Chinese herbal medicine (CHM) as an adjunctive therapy in distinct stages of patients with COVID-19: A systematic review and meta-analysis
Source: PLoS One. 2025 Feb 13;20(2):e0318892. doi: 10.1371/journal.pone.0318892 (PMC11825027; doi:10.1371/journal.pone.0318892)
Supplement: S1 Table — (DOCX) [file pone.0318892.s004.docx]

**Supplementary Table S1. Details of the search strategy of PubMed/ Embase/ Cochrane Library**

| **Number** | **Search terms** |
| --- | --- |
| **#1** | Corona Virus Disease 2019 [MeSH Terms] |
| **#2** | COVID-19 [Title/Abstract] |
| **#3** | 2019 novel coronavirus [Title/Abstract] |
| **#4** | SARS-CoV-2 [Title/Abstract] |
| **#5** | 2019-nCoV [Title/Abstract] |
| **#6** | coronavirus disease 2019 [Title/Abstract] |
| **#7** | #1-#7 / OR |
| **#8** | Traditional Chinese Medicine [MeSH Terms] |
| **#9** | TCM [Title/Abstract] |
| **#10** | Integrated traditional Chinese and Western medicine [Title/Abstract] |
| **#11** | Traditional Medicine, Chinese [Title/Abstract] |
| **#12** | Zhong Yi Xue [Title/Abstract] |
| **#13** | Chinese Traditional Medicine [Title/Abstract] |
| **#14** | Chinese Medicine, Traditional [Title/Abstract] |
| **#15** | traditional Chinese herbal medicine [Title/Abstract] |
| **#16** | Chinese medicine [Title/Abstract] |
| **#17** | #8-#17/ OR |
| **#18** | #7 AND #17 |
