# Supplementary material for: Effect of Chinese herbal medicine (CHM) as an adjunctive therapy in distinct stages of patients with COVID-19: A systematic review and meta-analysis
Source: PLoS One. 2025 Feb 13;20(2):e0318892. doi: 10.1371/journal.pone.0318892 (PMC11825027; doi:10.1371/journal.pone.0318892)
Supplement: S2 Table — (DOCX) [file pone.0318892.s005.docx]

**Supplementary Table S2. Details of the search strategy of Wan fang Database / VIP Information Database/ SinoMed/ China National Knowledge Infrastructure**

| **Number** | **Search terms** |
| --- | --- |
| **#1** | 新型冠状病毒 [主题] |
| **#2** | 新型冠状病毒肺炎 [主题] |
| **#3** | 新冠肺炎 [主题] |
| **#4** | #1-#3/ OR |
| **#5** | 中医 [主题] |
| **#6** | 中医药 [主题] |
| **#7** | 中草药 [主题] |
| **#8** | 中药 [主题] |
| **#9** | 中西医结合 [主题] |
| **#10** | #5-#9/ OR |
| **#11** | #4 AND #10 |
