# Supplementary material for: Effect of Chinese herbal medicine (CHM) as an adjunctive therapy in distinct stages of patients with COVID-19: A systematic review and meta-analysis
Source: PLoS One. 2025 Feb 13;20(2):e0318892. doi: 10.1371/journal.pone.0318892 (PMC11825027; doi:10.1371/journal.pone.0318892)
Supplement: S3 Table — (DOCX) [file pone.0318892.s006.docx]

**Supplementary Table S3. The reason for excluded studies at full-reading stage**

| **Title** | **Reason for excluded** |
| --- | --- |
| A Retrospective Study on the Treatment of Coronavirus Disease 2019 with "Pneumonia No.1" Combined with Conventional Western Medicine | enrolled patients don't meet the included criteria |
| Clinical efficacy study on Corona Virus Disease 2019 treated with ‘Pneumonia No.1 Formula’ | enrolled patients don't meet the included criteria |
| Multi-Center Clinical Study on 451 Cases of Corona Virus Disease Treated with “Pneumonia No. 1 Formula” | enrolled patients don't meet the included criteria |
| Study on the treatment of novel coronavirus pneumonia with "Pneumonia No. 1" | enrolled patients don't meet the included criteria |
| Treatment of 51 cases of COVID-19 nucleic acid Fuyang with traditional Chinese medicine | enrolled patients don't meet the included criteria |
| Clinical study of 61 patients with COVID-19 treated with Chinese medicine | enrolled patients don't meet the included criteria |
| Retrospective Analysis of Clinical Efficacy of Traditional Chinese Medicine in 87 Children with Corona Virus Disease 2019 | enrolled patients don't meet the included criteria |
| Effect of integrated traditional Chinese and Western medicine on T lymphocyte subsets of patients with normal type of COVID - 19 | enrolled patients don't meet the included criteria |
| Clinical Study on 36 Cases of Common COVID-19 Treated by Baihu Yinqiao Decoction Combined with Western Medicine | enrolled patients don't meet the included criteria |
| The role of syndrome differentiation in traditional Chinese-western medicine combined diagnosis and treatment of 105 cases of COVID-19 | enrolled patients don't meet the included criteria |
| Chinese Medicine Clinical Research and Thinking of 40 Cases of COVID-19 Treated by Stages | enrolled patients don't meet the included criteria |
| Clinical study on intervention of Fuzheng Hejie Recipe in children with asymptomatic infection of SARS⁃CoV⁃2 Omicron variant | enrolled patients don't meet the included criteria |
| Efficacy of Traditional Chinese Medicine on Children Infected with Novel Coronavirus Delta Variant in Fujian Province: An Analysis of 115 Cases | enrolled patients don't meet the included criteria |
| Clinical observation on integrated traditional Chinese and western medicine in the treatment of 51 cases of Corona Virus Disease 2019 in Hangzhou area | enrolled patients don't meet the included criteria |
| Clinical efficacy of the Houyanqing oral liquid plus conventional medication on immune indicators and inflammatory factors in ordinary COVID-19 | enrolled patients don't meet the included criteria |
| Clinical Observation of 31 Cases with COVID - 2019 Treated with Guizhi Erchen Decoction Based on Triple - Jiao Sequential Therapy | enrolled patients don't meet the included criteria |
| Treatment of 23Cases of Severe COVID-19with Huashi Baidu Decoction Combined with Western Medicine | enrolled patients don't meet the included criteria |
| Retrospective Analysis on 70 Cases of COVID-19 Treated with Integrated Traditional Chinese and Western Medicine | enrolled patients don't meet the included criteria |
| Clinical Observation on the Traditional Chinese Medicine Treatment for 61 Confirmed Cases of COVID-19 in Huizhou City | enrolled patients don't meet the included criteria |
| Huoxiang Zhengqi Powder combined with Western medicine to treat 11 cases of novel coronavirus pneumonia | enrolled patients don't meet the included criteria |
| Clinical study on the treatment of early novel coronavirus pneumonia based on Lianhua Qingwen Capsule combined with traditional Chinese and Western medicine | enrolled patients don't meet the included criteria |
| A real - world clinical study of ‘Pneumonia No. 1’in the treatment of COVID - 19 | enrolled patients don't meet the included criteria |
| Clinical Observation on Modified Jiegeng Xingren Decoction in Treating New Coronavirus Pneumonia Suspected Cases Type of Lung Qi Stagnation | enrolled patients don't meet the included criteria |
| Retrospective study of 170 cases of Corona Virus Disease 2019 in Jiangxia District Hospital of Traditional Chinese Medicine | enrolled patients don't meet the included criteria |
| Clinical Application Value of Jinshuibao Capsules in the Treatment of Sequelae of COVID-19 | enrolled patients don't meet the included criteria |
| Treatment of 39 cases of novel coronavirus with Jinxianlian liquid (spray) combined with Chinese medicine prescription granules | enrolled patients don't meet the included criteria |
| COVID-19 ( Omicron) Treatment with Jiuwei Qingwen Drink | enrolled patients don't meet the included criteria |
| Antiviral prescription No. 1 for the treatment of cold-dampness-depression-lung novel coronavirus pneumonia in the early and middle stages curative effect observation | enrolled patients don't meet the included criteria |
| Study on Clinical Efficacy of Lianhua Qingke Granule in Treatment of Mild and Ordinary COVID-19 | enrolled patients don't meet the included criteria |
| Analysis of Traditional Chinese Medicine Treatment of 60 Patients with Novel Coronavirus Pneumonia in Southwestern Shandong | enrolled patients don't meet the included criteria |
| Clinical study of Peiyuan Tongbii Chinese Medicine in the treatment of pulmonary fibrosis in convalescent stage of novel coronavirus pneumonia | enrolled patients don't meet the included criteria |
| Clinical study on adjuvant treatment of 35 cases of novel coronavirus pneumonia with Qianghopi Qushi Qingwen mixture | enrolled patients don't meet the included criteria |
| Clinical Study on the Preventive Effect of Qiangshen Kangyi Decoction on COVID-19 Infection | enrolled patients don't meet the included criteria |
| Clinical observation on the efficacy of Qingfeipaidu decoction on patients with common and severe corona virus disease 2019 | enrolled patients don't meet the included criteria |
| A retrospective study on the treatment of COVID-19 type common/type severe with Qinfei Paidu decoction | enrolled patients don't meet the included criteria |
| Clinical efficacy of Qingfei Detoxification Decoction in treating novel coronavirus pneumonia | enrolled patients don't meet the included criteria |
| Clinical Study on Qinghua Pihun Decoction in the Treatment of“ Long-term Positive” Patients with Novel Coronavirus Omicron Variant Infection | enrolled patients don't meet the included criteria |
| Clinical effect of modified Renshen Baidu powder in treatment of severe acute respiratory syndrome coronavirus 2 infection ( or pneumonia) with spleen-lung Qi deficiency and cold dampness stagnation in lungs: An analysis of 28 cases | enrolled patients don't meet the included criteria |
| A Retrospective Study of Mulberry Reed Stem Decoction in the Treatment of Patients Recovering from COVID-19 Infection with Phlegm-Dampness Accumulated in the Lung | enrolled patients don't meet the included criteria |
| Clinical effect analysis of Shengmai Powder on Qi-Yin deficiency syndrome in convalescent period of novel coronavirus pneumonia | enrolled patients don't meet the included criteria |
| Clinical efficacy of Liushenwan combined with conventional treatment in patients with COVID-19 | enrolled patients don't meet the included criteria |
| Real-world Clinical Study on Tanreqing Injection in the Treatment of Novel Coronavirus-infected Pneumonia | enrolled patients don't meet the included criteria |
| Analysis of TCM constitution types and clinical characteristics of 85 discharged patients with novel coronavirus pneumonia in Tianjin | enrolled patients don't meet the included criteria |
| The novel coronavirus No. 2 prescription treated 40 cases of cold dampness blocking lung syndrome of common novel coronavirus pneumonia | enrolled patients don't meet the included criteria |
| Observation on the clinical effect of Xuanfeixuedu granules in the treatment of novel coronavirus pneumonia (Omicron) | enrolled patients don't meet the included criteria |
| Clinical observation of Xue's Fuyang Zhushi Decoction for treatment of 36 patients with novel coronavirus pneumonia with difficulty in nucleic acid conversion to negative | enrolled patients don't meet the included criteria |
| Experience of Chaihu Guizhi Ganjiang Decoction in Treating Elderly Patients with Novel Coronavirus Omicron Mutant Infection | enrolled patients don't meet the included criteria |
| Yinqiao Powder combined with Sanren Decoction to treat 20 cases of common dampness-toxin pneumonitis syndrome of novel coronavirus pneumonia | enrolled patients don't meet the included criteria |
| Clinical study on the treatment of patients with post-COVID-19 syndrome by integration of traditional and western medicine rehabilitation program | enrolled patients don't meet the included criteria |
| Observation on the curative effect of integrated traditional Chinese and Western medicine in treating non-severe novel coronavirus pneumonia | enrolled patients don't meet the included criteria |
| On the treatment of one critically ill COVID-19 patient with the integrated Chinese and western medicine | enrolled patients don't meet the included criteria |
| Clinical study of 34 cases of novel coronavirus pneumonia treated by integrated Chinese and Western medicine | enrolled patients don't meet the included criteria |
| Retrospective analysis of 38 cases of novel coronavirus pneumonia treated by integrated Chinese and Western medicine | enrolled patients don't meet the included criteria |
| Clinical effect of integrated traditional Chinese and Western medicine therapy in treatment of the initial pyrexia stage of coronavirus disease 2019: An analysis of 43 cases | enrolled patients don't meet the included criteria |
| 1 case of integrated Chinese and Western medicine treating novel coronavirus pneumonia | enrolled patients don't meet the included criteria |
| Clinical Observation on Treating COVID-19 in Yueyang Area with Chinese Medicine | enrolled patients don't meet the included criteria |
| Clinical study of traditional Chinese medicine against Corona Virus Disease 2019 | enrolled patients don't meet the included criteria |
| Observation on the effect of TCM syndrome differentiation in treating novel coronavirus pneumonia | enrolled patients don't meet the included criteria |
| Two proved cases of TCM syndrome differentiation and treatment for elderly patients with COVID⁃19 infection complicated with underlying diseases | enrolled patients don't meet the included criteria |
| Effectiveness of Traditional Chinese Medicine in Reducing the Positive Rate of COVID-19 Close Contacts: A Large Population Cohort Study | enrolled patients don't meet the included criteria |
| Retrospective multi-center cohort study of traditional Chinese medicine early intervention in Corona Virus Disease 2019 | enrolled patients don't meet the included criteria |
| A Retrospective Study of 72 Cases of COVID-19 Patients with Delta Virus Strain Treated with Traditional Chinese Medicine | enrolled patients don't meet the included criteria |
| Clinical study on comprehensive rehabilitation program of traditional Chinese medicine for patients with different syndrome types in Corona Virus Disease 2019 recovery period | enrolled patients don't meet the included criteria |
| Multi-Center Clinical Study on Corona Virus Disease Treated with “ No.1 Formula” | enrolled patients don't meet the included criteria |
| Clinical research of 32 children with coronavirus disease 2019 in Hubei Province | enrolled patients don't meet the included criteria |
| Treatment of 51 cases of COVID-19 nucleic acid with traditional Chinese medicine | enrolled patients don't meet the included criteria |
| Clinical study of Baihu Yinqiao Decoction combined with Western medicine in treatment of 36 patients with common novel coronavirus pneumonia | enrolled patients don't meet the included criteria |
| The role of syndrome differentiation and treatment in the diagnosis and treatment of 105 cases of novel coronavirus pneumonia | enrolled patients don't meet the included criteria |
| Treatment of 23 cases of severe novel coronavirus pneumonia with Huashi-Du-dou recipe combined with Western medicine | enrolled patients don't meet the included criteria |
| Clinical application value of Jinshuibao capsule in treating sequelae of novel coronavirus pneumonia | enrolled patients don't meet the included criteria |
| Clinical study on adjuvant treatment of 35 cases of novel coronavirus pneumonia with Qianghopi Qushi Qingwen mixture | enrolled patients don't meet the included criteria |
| Observation on the curative effect of Qingfei Detoxification Decoction on patients with common and severe novel coronavirus pneumonia | enrolled patients don't meet the included criteria |
| Analysis of drug use and curative effect of integrated traditional Chinese and Western medicine in the treatment of novel coronavirus pneumonia in Tianjin | enrolled patients don't meet the included criteria |
| Clinical observation on prevention and treatment of novel coronavirus pneumonia by combining traditional Chinese and Western medicine as main medicine of Tujia Medicine | enrolled patients don't meet the included criteria |
| Clinical study of Xuanfei Dayu Decoction in the recovery stage of novel coronavirus pneumonia | enrolled patients don't meet the included criteria |
| Clinical study on the adjuvant treatment of common novel coronavirus pneumonia with Xuanfei Qingre prescription | enrolled patients don't meet the included criteria |
| Yinqiao Powder combined with Sanren Decoction to treat 20 cases of common dampness-toxin pneumonitis syndrome of novel coronavirus pneumonia | enrolled patients don't meet the included criteria |
| Clinical effect analysis of combined traditional Chinese and Western medicine therapy on 31 cases of severe novel coronavirus pneumonia | enrolled patients don't meet the included criteria |
| Clinical effect observation on 20 cases of mild and common cases of novel coronavirus pneumonia treated by integrated traditional Chinese and Western medicine | enrolled patients don't meet the included criteria |
| Observation of clinical efficacy of integrated traditional Chinese and Western medicine in treating non-critical novel coronavirus pneumonia | enrolled patients don't meet the included criteria |
| Evaluation of therapeutic effect of integrated traditional Chinese and Western medicine on patients infected with novel coronavirus in plateau area | enrolled patients don't meet the included criteria |
| Clinical study on the treatment of novel coronavirus pneumonia and asymptomatic infected patients with integrated traditional Chinese and Western medicine | enrolled patients don't meet the included criteria |
| Clinical review of the treatment of novel coronavirus pneumonia by integrated traditional Chinese and Western medicine | enrolled patients don't meet the included criteria |
| A retrospective study of 34 patients with novel coronavirus pneumonia treated by integrated Chinese and Western medicine | enrolled patients don't meet the included criteria |
| Clinical observation on the treatment of novel coronavirus pneumonia by combination of traditional Chinese and Western medicine | enrolled patients don't meet the included criteria |
| Evaluation of clinical effect of integrated Chinese and Western medicine in treating novel coronavirus pneumonia | enrolled patients don't meet the included criteria |
| Clinical study on the treatment of novel coronavirus pneumonia by combination of traditional Chinese and Western medicine | enrolled patients don't meet the included criteria |
| Clinical study of 40 cases of critical patients with novel coronavirus pneumonia treated by integrated Chinese and Western medicine | enrolled patients don't meet the included criteria |
| Clinical study on the treatment of novel coronavirus pneumonia by combination of traditional Chinese and Western medicine | enrolled patients don't meet the included criteria |
| Observation of clinical effect of integrated traditional Chinese and Western medicine on novel coronavirus pneumonia in Yueyang area | enrolled patients don't meet the included criteria |
| Clinical efficacy of combined traditional Chinese and Western medicine in treating 49 cases of non-critical novel coronavirus pneumonia in Shanghai | enrolled patients don't meet the included criteria |
| Observation on the curative effect of traditional Chinese medicine on 41 cases of lung Qi deficiency syndrome in the convalescent period of COVID-19 in Jiangxi | enrolled patients don't meet the included criteria |
| Clinical study on 30 cases of Qi-Yin deficiency syndrome in convalescent period of novel coronavirus pneumonia treated by TCM comprehensive therapy | enrolled patients don't meet the included criteria |
| Multi-Center Clinical Study on Corona Virus Disease Treated with “Pneumonia No.1 Formula” | enrolled patients don't meet the included criteria |
| Treatment of 40 cases of common novel coronavirus pneumonia by Maxingshigan Decoction combined with Western medicine | enrolled patients don't meet the included criteria |
| Clinical effect analysis of 50 cases of novel coronavirus pneumonia | enrolled patients don't meet the included criteria |
| Clinical observation of 50 cases of asymptomatic infection with novel coronavirus treated with integrated Chinese and Western medicine | enrolled patients don't meet the included criteria |
| Clinical study of Xuanfei Dayu Decoction in the recovery stage of novel coronavirus pneumonia | enrolled patients don't meet the included criteria |
| Clinical efficacy observation of Qiguan No. 1 prescription in treating suspected patients with novel coronavirus pneumonia | enrolled patients don't meet the included criteria |
| Clinical effect analysis of combined traditional Chinese and Western medicine therapy on 31 cases of severe novel coronavirus pneumonia | enrolled patients don't meet the included criteria |
| Clinical efficacy of combined traditional Chinese and Western medicine in treating 49 cases of non-critical novel coronavirus pneumonia in Shanghai | enrolled patients don't meet the included criteria |
| Clinical study on the treatment of novel coronavirus pneumonia and asymptomatic infected patients with integrated traditional Chinese and Western medicine | enrolled patients don't meet the included criteria |
| Clinical study of 40 cases of critical patients with novel coronavirus pneumonia treated by integrated Chinese and Western medicine | enrolled patients don't meet the included criteria |
| Observation on the effect of TCM syndrome differentiation in treating novel coronavirus pneumonia | enrolled patients don't meet the included criteria |
| Clinical features, course of disease and length of stay of patients with novel coronavirus pneumonia treated by traditional Chinese medicine | enrolled patients don't meet the included criteria |
| Exploring an Integrative Therapy for Treating COVID-19: A Randomized Controlled Trial | enrolled patients don't meet the included criteria |
| Efficacy of the combination of modern medicine and traditional Chinese medicine in pulmonary fibrosis arising as a sequela in convalescent COVID-19 patients: a randomized multicenter trial | enrolled patients don't meet the included criteria |
| Clinical characteristics and impacts of traditional Chinese medicine treatment on the convalescents of COVID-19 | enrolled patients don't meet the included criteria |
| Qingjin Yiqi granules for post-COVID-19 condition: A randomized clinical trial | enrolled patients don't meet the included criteria |
| Effectiveness of Xiaoyao capsule on sleep disorders and mood disturbance in patients in recovery from coronavirus disease 2019: a randomized controlled trial | enrolled patients don't meet the included criteria |
| Efficacy of Huoxiang Zhengqi dropping pills and Lianhua Qingwen granules in treatment of COVID-19: A randomized controlled trial | enrolled patients don't meet the included criteria |
| Effects of Shuanghuanglian oral liquids on patients with COVID-19: a randomized, open-label, parallel-controlled, multicenter clinical trial | enrolled patients don't meet the included criteria |
| Effects and safety of herbal medicines among community-dwelling residents during COVID-19 pandemic: A large prospective, randomized controlled trial (RCT) | enrolled patients don't meet the included criteria |
| The effectiveness and safety of traditional Chinese medicine for the treatment of children with COVID-19 | enrolled patients don't meet the included criteria |
| Efficacy and safety of Bufei Huoxue capsules in the management of convalescent patients with COVID-19 infection: A multicentre, double-blind, and randomised controlled trial | enrolled patients don't meet the included criteria |
| Huashi baidu granule in the treatment of pediatric patients with mild coronavirus disease 2019: A single-center, open-label, parallel-group randomized controlled clinical trial | enrolled patients don't meet the included criteria |
| The efficacy of combined therapy of qingfeiPaidu capsule and lianhuaqingwen capsule nursing interventions for hospitalized patients with COVID-19 | enrolled patients don't meet the included criteria |
| Ludangshen oral liquid for treatment of convalescent COVID-19 patients: Chinese Medicine  a randomized, double-blind, placebo-controlled multicenter trial | enrolled patients don't meet the included criteria |
| Shugan Jieyu capsule improve sleep and emotional disorder in coronavirus disease 2019 convalescence patients: a randomized, double-blind, placebo-controlled trial | enrolled patients don't meet the included criteria |
| Effects of Shengmai Yin on pulmonary and cardiac function in coronavirus disease 2019 convalescent patients with cardiopulmonary symptoms: a randomized, double blind, multicenter control trial | enrolled patients don't meet the included criteria |
| Early therapeutic interventions of traditional Chinese medicine in COVID-19 patients: A retrospective cohort study | enrolled patients don't meet the included criteria |
| Effectiveness and safety of Jinshuibao capsules in treatment of residual cardiopulmonary symptoms in convalescent patients of coronavirus disease 2019: a pilot randomized, double-blind, placebo-controlled clinical trial | enrolled patients don't meet the included criteria |
| Treatment of COVID-19 in Hemodialysis Patients Using Traditional Chinese Medicine: A Single-Center, Retrospective Study | enrolled patients don't meet the included criteria |
| Efficacy and safety of Bufei Huoxue capsules in the management of convalescent patients with COVID-19 infection: A multicentre, double-blind, and randomised controlled trial | enrolled patients don't meet the included criteria |
| Clinical retrospective study on the efficacy of Qingfei Paidu decoction combined with Western medicine for COVID-19 treatment | enrolled patients don't meet the included criteria |
| Effect of Lianhua Qingwen capsules on the positive rate of COVID-19 close contacts: A retrospective analysis of a large-scale population-based cohort study | enrolled patients don't meet the included criteria |
| Xuebijing injection in the treatment of COVID-19: a retrospective case-control study | enrolled patients don't meet the included criteria |
| Effectiveness of Xiangsha Liujun pills on decreased digestive function in convalescent patients of coronavirus disease 2019: a randomized, double blind, placebo controlled clinical trial | enrolled patients don't meet the included criteria |
| Huashi baidu granule in the treatment of pediatric patients with mild coronavirus disease 2019: A single-center, open-label, parallel-group randomized controlled clinical trial | enrolled patients don't meet the included criteria |
| Jin-Zhen oral liquid for pediatric coronavirus disease (COVID-19): A randomly controlled, open-label, and non-inferiority trial at multiple clinical centers | enrolled patients don't meet the included criteria |
| A retrospective study of Reyanning mixture in elderly patients infected with SARS-CoV-2 Omicron variant | enrolled patients don't meet the included criteria |
| Qingjin Yiqi granules for post-COVID-19 condition: A randomized clinical trial | enrolled patients don't meet the included criteria |
| Clinical analysis and discussion of tongue image in 133 patients infected with Delta variant of novel coronavirus | Not RCT, PCS or RCS |
| Analysis of dynamic changes of clinical symptoms and TCM diagnosis and treatment characteristics of 571 patients infected with mild novel coronavirus | Not RCT, PCS or RCS |
| Study on 69 cases in Wuhan area from the theory of qi and blood | Not RCT, PCS or RCS |
| Clinical characteristics of traditional Chinese medicine in Delta novel coronavirus pneumonia complicated with tuberculosis | Not RCT, PCS or RCS |
| TCM syndromes and treatment strategies of 72 cases of novel coronavirus pneumonia in Sanya City, Hainan Province | Not RCT, PCS or RCS |
| TCM clinical features and syndrome distribution of 86 children infected with Omicron variant of novel coronavirus pneumonia in Henan Province | Not RCT, PCS or RCS |
| Analysis of TCM syndromes and physical and chemical indexes of 87 children with COVID-19 in Putian City | Not RCT, PCS or RCS |
| TCM syndromes of 112 elderly patients infected with Omicron variant of novel coronavirus in Shanghai | Not RCT, PCS or RCS |
| Analysis of TCM syndromes of 99 children with COVID-19 in Shijiazhuang area | Not RCT, PCS or RCS |
| Analysis of the characteristics of "long novel coronavirus" and TCM syndromes in 746 patients infected with novel coronavirus in Tianjin | Not RCT, PCS or RCS |
| Clinical features and TCM syndromes of 57 cases of novel coronavirus pneumonia in Wuhan area | Not RCT, PCS or RCS |
| Xixi Novel Coronavirus No. 3 prescription treated 40 cases of common novel coronavirus pneumonia | Not RCT, PCS or RCS |
| Clinical effect observation of Xuanfei HuozhuoJiajia decoction in treating 40 cases of novel coronavirus pneumonia | Not RCT, PCS or RCS |
| Clinical follow-up study on intervention of severe and critical COVID-19 patients with comprehensive rehabilitation program of traditional Chinese medicine | Not RCT, PCS or RCS |
| Evaluation of clinical efficacy of Xuanfeixuedu granules in the treatment of novel coronavirus pneumonia infected by Omicron strain | Not RCT, PCS or RCS |
| Clinical observation on the treatment of severe novel coronavirus pneumonia by Xuanfei Xiedu Decoction | Not RCT, PCS or RCS |
| Traditional Chinese Medicine syndromes of 96 cases of common novel coronavirus pneumonia in northern China and observation of therapeutic effect of decoction | Not RCT, PCS or RCS |
| Clinical retrospective analysis of 608 cases of outpatient and emergency patients with novel coronavirus pneumonia | Not RCT, PCS or RCS |
| Retrospective analysis of clinical efficacy of 70 cases of mild novel coronavirus pneumonia | Not RCT, PCS or RCS |
| Retrospective study on clinical efficacy of Qingfei Detoxification Decoction combined with conventional western medicine in treating 157 cases of novel coronavirus pneumonia in the elderly | Not RCT, PCS or RCS |
| Retrospective analysis of clinical efficacy of Qingfei Detoxification Decoction combined with conventional western medicine in treating 50 cases of severe novel coronavirus pneumonia | Not RCT, PCS or RCS |
| Clinical effect analysis of Qufeidu No. 1 prescription in the treatment of severe/critical patients with novel coronavirus pneumonia | Not RCT, PCS or RCS |
| Analysis of curative effect and liver injury of 100 cases of novel coronavirus pneumonia treated by integrated traditional Chinese and Western medicine | Not RCT, PCS or RCS |
| Clinical observation on the treatment of mild and ordinary patients with novel coronavirus infection by expelling lung and clearing heat and eliminating dampness in northern Anhui | Not RCT, PCS or RCS |
| Clinical observation of Yinchaihoplan Decoction in treating mild and common novel coronavirus pneumonia | Not RCT, PCS or RCS |
| A real-world study on the clinical characteristics and TCM treatment of novel coronavirus pneumonia in Ruili, Yunnan Province | Not RCT, PCS or RCS |
| Observation on the effect of 70 cases of novel coronavirus pneumonia treated by TCM syndrome differentiation | Not RCT, PCS or RCS |
| Syndrome differentiation and treatment of 84 cases of novel coronavirus in Haozhou City | Not RCT, PCS or RCS |
| Clinical observation on the treatment of common novel coronavirus pneumonia from "cold dampness epidemic" | Not RCT, PCS or RCS |
| Retrospective analysis of clinical efficacy of 70 cases of mild novel coronavirus pneumonia | Not RCT, PCS or RCS |
| Clinical effect analysis of 50 cases of novel coronavirus pneumonia | Not RCT, PCS or RCS |
| Clinical observation of 50 cases of asymptomatic infection with novel coronavirus treated with integrated Chinese and Western medicine | Not RCT, PCS or RCS |
| Observation on the effect of Chinese and Western combined treatment of novel coronavirus pneumonia | Not RCT, PCS or RCS |
| Clinical observation of non-critical novel coronavirus pneumonia treated by integrated Chinese and Western medicine | Not RCT, PCS or RCS |
| Observation on the curative effect of integrated traditional Chinese and Western medicine in treating non-severe novel coronavirus pneumonia | Not RCT, PCS or RCS |
| The role of syndrome differentiation and treatment in the diagnosis and treatment of 105 cases of novel coronavirus pneumonia | Not RCT, PCS or RCS |
| Combined traditional Chinese and western medicine nursing 60 cases of novel coronavirus pneumonia | Not RCT, PCS or RCS |
| Clinical features and the traditional Chinese medicine therapeutic characteristics of 293 COVID-19 inpatient cases | Not RCT, PCS or RCS |
| Traditional Chinese Medicine Formulation Therapy in the Treatment of Coronavirus Disease 2019 (COVID-19) | Not RCT, PCS or RCS |
| The efficacy of traditional Chinese medicine in the treatment of the COVID-19 pandemic in Henan Province: a retrospective study | Not RCT, PCS or RCS |
| Effectiveness of Traditional Chinese Medicine on coronavirus disease 2019 in 92 patients: a retrospective study | Not RCT, PCS or RCS |
| Treatment Effects of Integrated TCM and Western Medicine Treatment Scheme on COVID-19: A Single-armed Clinical Trial | Not RCT, PCS or RCS |
| Treatment Effect of Qingfei Paidu Decoction Combined with Conventional Treatment on COVID-19 Patients and Other Respiratory Diseases: A Multi-Center Retrospective Case Series | Not RCT, PCS or RCS |
| Clinical study of 1055 patients with severe COVID-19 treated by integrated traditional Chinese and western medicine | Master/PhD thesis |
| Clinical Efficacy Study of "removing dampness and clearing the lung" on 60 Patients with COVID-19 Syndrome of Damp-heat Inclusion of Lung | Master/PhD thesis |
| Clinical study on the efficacy and safety of Xuanfei Zhishou mixture in treating cough infected with novel coronavirus | Insufficient outcome indicators |
| Retrospective study on the treatment of 72 patients with "Delta" strain novel coronavirus pneumonia with traditional Chinese medicine | Insufficient outcome indicators |
| Clinical observation of Ganjiang Xiaochaihu decoction in treating mild infection of novel coronavirus Omicron variant | Insufficient outcome indicators |
| Positive effects of Lianhuaqingwen granules in COVID-19 patients: A retrospective study of 248 cases | Insufficient outcome indicators |
| Effects of Tanreqing Capsule on the negative conversion time of nucleic acid in patients with COVID-19: A retrospective cohort study | Insufficient outcome indicators |
| The effect of Huashibaidu formula on the blood oxygen saturation status of severe COVID-19: A retrospective cohort study | Insufficient outcome indicators |
| Observation on the curative effect of "specimen combined with point" fireneedle in the treatment of 33 cases of sequelae of novel coronavirus pneumonia during convalescence | Interventions were not ICW/CWM |
| Preliminary study on clinical efficacy of "Qingfei Detoxification granule" in treating novel coronavirus pneumonia | Interventions were not ICW/CWM |
| Clinical efficacy of Anshen Granule in treating 110 cases of novel coronavirus pneumonia complicated with insomnia | Interventions were not ICW/CWM |
| A retrospective comparative study of traditional Chinese medicine treatment in 766 patients infected with novel coronavirus Omicron variant | Interventions were not ICW/CWM |
| Based on the "Novel Coronavirus Infection Diagnosis and Treatment Plan (Trial version 10)" to explore the optimal clinical efficacy of traditional Chinese medicine combined with moxibustion patch in the treatment of patients recovering from novel coronavirus infection | Interventions were not ICW/CWM |
| Effect of combined Chinese medicine therapy on kidney injury in patients with novel coronavirus pneumonia | Interventions were not ICW/CWM |
| Thunder fire moxibustion combined with lung clearing and detoxification Decoction to treat clinical symptoms is in line with the clinical effect of cold dampness blocking lung syndrome novel coronavirus pneumonia in children | Interventions were not ICW/CWM |
| Qingfei Detoxification Decoction combined with auricular point sticking pressure and acupoint sticking treatment for mild novel coronavirus pneumonia | Interventions were not ICW/CWM |
| Observation of curative effect of Sanhao Yurong Mixture on novel coronavirus pneumonia | Interventions were not ICW/CWM |
| Observation on the effect of suspension moxibustion combined with acupoint application on the prevention of novel coronavirus pneumonia infection | Interventions were not ICW/CWM |
| Clinical study on the treatment of children with novel coronavirus pneumonia by Yifei Jianpi massage during medical observation | Interventions were not ICW/CWM |
| Clinical study of Yiqi Jiedu prescription for treating novel coronavirus pneumonia | Interventions were not ICW/CWM |
| Therapeutic effect of acupuncture in 32 cases of novel coronavirus pneumonia | Interventions were not ICW/CWM |
| Clinical effect analysis of Buzhong Yiqi Decoction in treating mild novel coronavirus pneumonia | Interventions were not ICW/CWM |
| Clinical observation of Fuzheng Xuanfei Huashui prescription on novel coronavirus pneumonia | Interventions were not ICW/CWM |
| Clinical study of Gegen Qinlian pills for treating novel coronavirus pneumonia | Interventions were not ICW/CWM |
| Clinical observation of Toujie Quwen Granule in treating novel coronavirus pneumonia | Interventions were not ICW/CWM |
| Clinical observation of viral pneumonia prescription in intervention of novel coronavirus infection | Interventions were not ICW/CWM |
| A real-world study of Shenmaiing Feifang for the treatment of 223 patients infected with COVID-19 | Interventions were not ICW/CWM |
| The clinical study of Jiangxia shelter series prescription for the treatment of mild novel coronavirus pneumonia | Interventions were not ICW/CWM |
| Observation of curative effect of Maxingxuanfei Jiedu Decoction on common novel coronavirus pneumonia | Interventions were not ICW/CWM |
| Clinical treatment of 374 patients with pneumonia complicated with anxiety and depression caused by novel coronavirus infection by TCM psychological sand table | Interventions were not ICW/CWM |
| Clinical observation on the treatment of novel coronavirus pneumonia by comprehensive nursing of traditional Chinese medicine | Interventions were not ICW/CWM |
| Traditional Chinese Medicine JingYinGuBiao Formula Therapy Improves the Negative Conversion Rate of SARS-CoV2 in Patients with Mild COVID-19 | Interventions were not ICW/CWM |
| Clinical evaluation of Shufeng Jiedu Capsules combined with Arbidol in the treatment of common-type COVID-19: a retrospective study | Interventions were not ICW/CWM |
| Effectiveness and Safety of Baidu Jieduan Granules for COVID-19: A Retrospective Observational Multicenter Study | Interventions were not ICW/CWM |
| Efficacy and Safety of Huashi Baidu Granules in Treating Patients with SARS-CoV-2 Omicron Variant: A Single-Center Retrospective Cohort Study | Interventions were not ICW/CWM |
| A retrospective study of Pupingqinghua prescription versus Lianhuaqingwen in Chinese participants infected with SARS-CoV-2 Omicron variants | Interventions were not ICW/CWM |
| Acupuncture or cupping plus standard care versus standard care in moderate to severe COVID-19 patients: An assessor-blinded, randomized, controlled trial | Interventions were not ICW/CWM |
| Efficacy and safety of Chinese herbal medicine versus Lopinavir-Ritonavir in adult patients with coronavirus disease 2019: A non-randomized controlled trial | Interventions were not ICW/CWM |
| Effectiveness of Liu-zi-jue exercise on coronavirus disease 2019 in the patients: a randomized controlled trial | Interventions were not ICW/CWM |
| Qigong exercise enhances cognitive functions in the elderly via an interleukin-6-hippocampus pathway: A randomized active-controlled trial | Interventions were not ICW/CWM |
| Arbidol combined with the Chinese medicine Lianhuaqingwen capsule versus arbidol alone in the treatment of COVID-19 | Interventions were not ICW/CWM |
| Jinhua Qinggan Granules for non-hospitalized COVID-19 Patients: a Double-Blind, Placebo-Controlled, Randomized Controlled Trial | Interventions were not ICW/CWM |
| Effectiveness and Safety of Lianhua Qingwen Capsules for COVID-19: A Propensity-Score Matched Cohort Study | Interventions were not ICW/CWM |
| Jinhua Qinggan granules for non-hospitalized COVID-19 patients: A double-blind, placebo-controlled, and randomized controlled trial | Interventions were not ICW/CWM |
| A retrospective study of Pupingqinghua prescription versus Lianhuaqingwen in Chinese participants infected with SARS-CoV-2 Omicron variants | Interventions were not ICW/CWM |
| The efficacy of combined therapy of qingfeiPaidu capsule and lianhuaqingwen capsule nursing interventions for hospitalized patients with COVID-19 A retrospective study of medical records | Interventions were not ICW/CWM |

Abbreviation: RCT: randomized controlled trial; RCS: retrospective cohort study; PCS: prospective cohort study
