# Supplementary material for: Effect of Chinese herbal medicine (CHM) as an adjunctive therapy in distinct stages of patients with COVID-19: A systematic review and meta-analysis
Source: PLoS One. 2025 Feb 13;20(2):e0318892. doi: 10.1371/journal.pone.0318892 (PMC11825027; doi:10.1371/journal.pone.0318892)
Supplement: S4 Table — (DOCX) [file pone.0318892.s007.docx]

**Supplementary Table S4. General information of included studies**

| **Study** | **Sample characteristics** | **Treatment** | | **Design** | **Outcome** |
| --- | --- | --- | --- | --- | --- |
|  |  | **Experiment** | **Control** |  |  |
| **QIN Lingxi^[22]^**  ***et al.* 2021** | ICW:523/CWM:40   - 92% severe COVID-19, 8% critical COVID-19 - Hypertension (26.4%), diabetes (11.6%), coronary heart disease (9.0%) | TCM decoction and oral Chinese patent medicine on the basis of the WMT | WMT   - Antiviral therapy: arbidol (100 mg/tablet), orally, 2 tablets at a time - Lopinavir Ritonavir (0.5 g, intravenous, once daily) - Antitussive phlegm therapy (ambroxol, 10 mL orally, 3 times a day) - Antibacterial therapy: moxifloxacin hydrochloride 0.4 g, once daily - α-interferon atomized inhalation (5 million U in 2 mL sterilized water, twice daily) - Other therapeutic measures: short-term use of glucocorticoids | RCS   - multicenter | ①③⑤⑥ |
| **HUANG Donghui^[23]^**  ***et al.***  **2021** | ICW:25/ CWM:25   - severe COVID-19 | Gengzi No.3 decoction on the basis of the WMT   - Oral, 200ml, one dose a day, divided into two doses | WMT   - Antiviral therapy: arbidol (200 mg/tablet), orally, 2 tablets at a time - Antibacterial therapy: moxifloxacin hydrochloride 0.4 g, once daily - Immune modulators | RCS | ①④⑬⑮ |
| **LUO Zhihui^[24]^**  ***et al.***  **2021** | ICW:26/CWM:26   - 19.2% mild COVID-19, 80.8% moderate COVID-19 - Hypertension (26.9%), diabetes (21.2%), tuberculosis (3.8%), stroke (17.3%） | Sanxiaoyin decoction on the basis of the WMT   - Oral, 300ml, one dose a day, divided into two doses | WMT   - Lopinavir Ritonavir (0.5 g, intravenous, once daily) - Antiviral therapy: α-interferon atomized inhalation (5 million U in 2 mL sterilized water, twice daily) - Supplemental oxygen - Nutrition therapy | RCS | ①②⑥⑦⑧⑩⑪⑫⑭⑮ |
| **YE Ling^[25]^**  ***et al.***  **2021** | ICW:50/CWM:50   - moderate COVID-19 - Hypertension (1%), diabetes (3%) | Shengjiang decoction on the basis of the WMT   - Oral, one dose a day, divided into two doses | WMT   - Antiviral therapy: arbidol (200 mg/tablet), orally, 2 tablets at a time | RCT   - 6d | ②⑪ |
| **YU Ping^[26]^**  ***et al.***  **2020** | ICW:147/CWM:148   - 9.2% mild COVID-19, 90.8% moderate COVID-19 - Hypertension (13.2%), diabetes (6.3%), coronary heart disease (10.6%) | Lianhua Qingwen granule on the basis of the WMT   - Oral, 1 sachet/time, thrice daily | WMT   - Antiviral therapy: arbidol (200 mg/tablet), orally, 2 tablets at a time - Antitussive phlegm therapy (ambroxol, 10 mL orally, 3 times a day) - Antibacterial therapy(moxifloxacin hydrochloride, 0.4 g, once daily) | RCT   - 7d | ①②⑫⑭ |
| **ZENG Xianhong^[27]^**  ***et al.***  **2020** | ICW:104/CWM:125   - moderate COVID-19 | Qingfei Paidu decoction on the basis of the WMT | WMT   - Lopinavir Ritonavir (0.5 g, intravenous, once daily) - Supplemental oxygen | RCS | ①④⑮ |
| **YANG Mingbo^[28]^**  ***et al.***  **2020** | ICW:26/CWM:23   - moderate COVID-19 | Reyanning liquid on the basis of the WMT   - Oral, 10mL~20mL/time, 2~4 times daily | WMT   - Antiviral therapy: arbidol (100 mg/tablet), orally, 2 tablets at a time, α-interferon atomized inhalation (5 million U in 2 mL sterilized water, twice daily) - Lopinavir Ritonavir (0.5 g, intravenous, once daily) | RCT   - multicenter - 7d | ①⑮ |
| **WANG Lin^[29]^**  ***et al.***  **2020** | ICW:40/CWM:40   - moderate COVID-19 | Shengmai decoction combined with Shenling Baizhu decoction on the basis of the WMT   - Oral, 200 ml, one dose a day, divided into two doses | WMT   - General treatment - Supplemental oxygen - Mechanical ventilation - Antibiotic agents - Antiviral therapy | RCT   - multicenter - 14d | ①②⑥⑧⑪ |
| **WANG Yuan^[30]^**  ***et al.***  **2022** | ICW:80/CWM:40   - severe/ critical COVID-19 | TCM decoction combined Chinese medicine injection and oral Chinese patent medicine on the basis of the WMT | WMT   - General treatment - Supplemental oxygen - Mechanical ventilation - Antibiotic agents - Antiviral therapy - Antitussive phlegm therapy | RCT   - 28d | ①③④⑪⑫⑭ |
| **CHEN Fei^[31]^**  ***et al.***  **2022** | ICW:30/CWM:45   - moderate COVID-19 | TCM decoction on the basis of the WMT   - Oral, 400 ml, one dose a day, divided into two doses | WMT   - Antiviral therapy (arbidol, 100 mg/tablet, orally, 2 tablets at a time) - Lopinavir Ritonavir (0.5 g, intravenous, once daily) - Antitussive phlegm therapy (ambroxol, 10 mL orally, 3 times a day) - Antibacterial therapy (moxifloxacin hydrochloride 0.4 g, once daily) - α-interferon atomized inhalation (5 million U in 2 mL sterilized water, twice daily) | RCS | ④⑮ |
| **YANG Qian^[32]^**  ***et al.***  **2020** | ICW:51/CWM:52   - 63.1% severe COVID-19, 36.9% critical COVID-19 - Hypertension (28.2%), diabetes (10.7%), coronary heart disease (10.6%), COPD (18.4%), cerebrovascular disease (18.4%), cancer (15.5%), chronic liver disease(17.5%), chronic kidney disease (19.4%) | TCM decoction on the basis of the WMT   - Oral, 400 ml, one dose a day, divided into two doses | WMT   - General treatment - Supplemental oxygen - Mechanical ventilation - Antibiotic agents - Antiviral therapy | RCS | ①③⑫⑮ |
| **JI Dan^[33]^**  ***et al.***  **2020** | ICW:28/CWM:22   - moderate COVID-19 | COVID- 19 formula granule on the basis of the WMT   - Oral, twice daily | WMT   - Antiviral therapy (arbidol, 100 mg/tablet, orally, 2 tablets at a time) - Lopinavir Ritonavir (0.5 g, intravenous, once daily) - Antitussive phlegm therapy (ambroxol, 10 mL, orally, 3 times a day) - Antibacterial therapy (moxifloxacin hydrochloride 0.4 g, once daily) - α-interferon atomized inhalation (5 million U in 2 mL sterilized water, twice daily) | RCS | ②⑤⑦⑩ |
| **YAO Kaitao^[34]^**  ***et al.***  **2020** | ICW:21/CWM:21   - moderate COVID-19 | Lianhua Qingwen granule on the basis of the WMT   - Oral, 1 sachet/time, thrice daily | WMT   - General treatment - Supplemental oxygen - Mechanical ventilation - Antibiotic agents - Antiviral therapy | RCS | ⑤⑥⑨⑩ |
| **Ya-Hui Li^[35]^**  ***et al.***  **2021** | ICW:50/CWM:46   - mild/ moderate COVID-19 - Hypertension (12.5%), diabetes (5.2%), coronary heart disease (4.2%), lung diseases (4.2%) | Jinbei Oral Liquid on the basis of the WMT   - Oral,10 mL/time, thrice daily | WMT   - Antiviral therapy: arbidol, atomized inhalation of interferon α-2b, intravenous ribavirin - Antibacterial therapy (cefoperazone sodium, 2 g, intravenous) | RCS | ⑦⑧⑨ |
| **Yue Zhang^[36]^**  ***et al.*  2023** | ICW:50/CWM:47   - mild COVID-19 | No.1/ No.2 /No.4 Mobile Cabin Hospital granules   - Oral, twice daily | WMT   - Antitussive phlegm therapy (ambroxol, 10 mL orally, 3 times a day) - Thymalfasin (1.6 mg, subcutaneous injection, twice a week) - Antibacterial therapy (moxifloxacin hydrochloride 0.4 g, once daily) - Low molecular weight heparin sodium (2500 IU subcutaneous injection, once daily) | RCT   - 14d | ⑤⑦⑩⑪⑫ |
| **Can Duan^[37]^**  ***et al.***  **2020** | ICW:82/CWM:41   - mild COVID-19 - Hypertension (28.5%), diabetes (23.6%), coronary heart disease (4.2%), COPD (4.1%), tuberculosis (3.3%), chronic kidney disease (2.4%), stroke (8.1%) | Jinhua Qinggan granule on the basis of the WMT   - Oral, 5 g/sachet, 2 sachets at a time, thrice daily. | WMT   - General treatment - Supplemental oxygen - Mechanical ventilation - Antibiotic agents - Antiviral therapy | RCT   - 5d | ②⑦⑨ |
| **Fei-Fei Lin^[38]^**  ***et al.***  **2020** | ICW:41/CWM:41   - moderate COVID-19 | Xuanefei Qingre decoction   - Oral, 300 ml, one dose a day, divided into two doses | WMT   - Lopinavir Ritonavir (500 mg twice daily, orally) - α-interferon atomized inhalation (5 million U in 2 mL sterilized water, twice daily) | RCT   - 14d | ①②④⑮ |
| **Chao Qun Huang^[39]^**  ***et al.***  **2023** | ICW^a^:1327/CWM^a^:76 ICW^b^:523/CWM^b^:40   - 71.6% moderate COVID-19, 28.4%severe COVID-19 | TCM decoction on the basis of the WMT   - Oral, 200 ml, one dose a day, divided into two doses | WMT   - Antiviral therapy: arbidol, atomized inhalation of interferon α-2b, intravenous ribavirin - Antibacterial therapy: cefoperazone sodium (2 g, intravenous) - Antitussive phlegm therapy (ambroxol) - Immune modulators - Corticosteroids therapy | RCS   - multicenter | ①③④ |
| **YAN Xiangyong^[40]^**  ***et al.***  **2021** | ICW:90/CWM:88   - moderate COVID-19 - Lung disease (22.5%) | Wenyang Huashi decoction on the basis of the WMT   - Oral, 200 ml, one dose a day, divided into two doses | WMT   - General treatment - Supplemental oxygen - Mechanical ventilation - Antibiotic agents - Antiviral therapy | PCS   - 10d | ②⑤⑦⑨ |
| **Fei He^[41]^**  ***et al.***  **2022** | ICW^a^:16/ICW^b^:16/CWM:15   - severe COVID-19 - Hypertension (29.8%), diabetes (12.8%)，lung disease (8.5%), chronic digestive disease (10.7%) | TCM decoction on the basis of the WMT   - Oral, 200 ml, one dose a day, divided into two doses | WMT   - Highest oxygen therapy support (non-invasive mechanical ventilation/invasive mechanical ventilation/extracorporeal membrane oxygenation or mechanical ventilation) - Antiviral therapy: oseltamivir (75 mg/tablet, orally, 1 tablet at a time, twice daily), arbidol (100 mg/tablet, orally, 2 tablets at a time, thrice daily), atomized inhalation of interferon α-2b, and intravenous ribavirin - Other therapy: symptomatic treatment and/or immunoglobulin intravenous injection and/or serum albumin intravenous injection, and treatment for basic disease | RCS | ①③ |
| **QIAN Yu-jun^[42]^**  ***et al.***  **2020** | ICW:170/CWM:130   - mild COVID-19 | TCM decoction on the basis of the WMT   - Oral, 300 ml, one dose a day, divided into two doses | WMT   - Antiviral therapy: oseltamivir (75 mg/tablet, orally, 1 tablet at a time, twice daily), arbidol (100 mg/tablet, orally, 2 tablets at a time, thrice daily), atomized inhalation of interferon α-2b, and intravenous ribavirin - Antibacterial therapy (cefoperazone sodium, 2 g, intravenous) - Supplemental oxygen therapy | RCS | ⑥⑪⑮ |
| **Hong-Ling Li^[43]^**  ***et al.***  **2020** | ICW:169/CWM:22   - moderate COVID-19 | TCM decoction on the basis of the WMT   - Oral, 300 ml, one dose a day, divided into two doses | WMT   - Antiviral therapy: oseltamivir (75 mg/tablet, orally, 1 tablet at a time, twice daily), and arbidol (100 mg/tablet, orally, 2 tablets at a time, thrice daily), atomized inhalation of interferon α-2b, intravenous ribavirin - Antibacterial therapy: cefoperazone sodium (2 g, intravenous) - Supplemental oxygen therapy | RCS | ⑥⑧⑪⑫⑭⑮ |
| **ZHOU Tong^[44]^**  ***et al.***  **2023** | ICW:55/CWM:56   - mild/ moderate COVID-19 - Hypertension (29.8%), diabetes (12.8%), lung disease (8.5%), digestive disease(10.7%) | Chonglian oral liquid on the basis of the WMT   - Oral, a dose of 20 mL/time, twice daily. | WMT   - Antibacterial (cefoperazone sodium, 2 g, intravenous) - Antivirus (oseltamivir, 75 mg, oral) - Immunotherapy (methylprednisolone, 20 mg, intravenous) | RCT   - 10d | ①②④⑪⑫⑬⑭ |
| **Zhi-Dan Lu^[45]^**  ***et al.***  **2022** | ICW^a^:15/CWM^a^:16 ICW^b^:15/CWM^b^:14   - 51.7%mild COVID-19/ moderate COVID-19, 48.3% severe/ critical COVID-19 | Chinese medicine injection and oral Chinese patent medicine on the basis of the WMT | WMT   - Lopinavir Ritonavir (500 mg twice daily, orally) - Antiviral therapy: oseltamivir (75 mg/tablet, orally, 1 tablet at a time, twice daily), arbidol (100 mg/tablet, orally, 2 tablets at a time, thrice daily), atomized inhalation of interferon α-2b, and intravenous ribavirin | RCS | ⑮ |
| **Jia Liu^[46]^**  ***et al.***  **2021** | ICW:94/CWM:96   - moderate COVID-19 - Hypertension (60%), hyperlipemia (60%), coronary heart disease (5.1%), diabetes (13,8%) | Huashi Baidu granule on the basis of the WMT   - Oral, a dose of 10 g/sachet, twice daily | WMT   - General treatment - Supplemental oxygen - Mechanical ventilation - Antibiotic agents - Antiviral therapy | RCT   - 14 d - open label | ①②⑥ |
| **Nannan Shi^[47]^**  ***et al.***  **2021** | ICW:20/CWM:20   - mild/ moderate COVID-19 - Cardiovascular and cerebrovascular diseases (36.7%), digestive system disease (11.7%), endocrine system disease (15%), malignant tumor (8.3%), nervous system disease (5%), respiratory system disease (8.3%) | Huashi Baidu decoction on the basis of the WMT   - Oral, 137 g, twice daily | WMT   - Lopinavir Ritonavir (500 mg twice daily, orally) - Antibiotics: cefoperazone(2 g twice daily, intravenous injection), moxifloxacin (hydrochloride tablets, 0.4 g once daily, orally) - Corticosteroids: methylprednisolone (40 mg once daily, intravenous injection), prednisone (30 mg once daily, orally) - Antiviruses (arbidol capsule, 0.2 g three times daily, orally) - Supportive therapy: oxygen inhalation, symptomatic treatment and/or immunoglobulin intravenous injection and/or serum albumin intravenous injection, and treatment for basic disease | PCS   - 16d | ⑪⑬⑭⑮ |
| **Xin-Yi Zhang^[48]^**  ***et al.***  **2021** | ICW:75/CWM:75   - mild/moderate COVID-19 - Hypertension (35.4%), diabetes (7.7%), hyperlipidemia (1.5%) | Xiyanping injection on the basis of the WMT   - Intravenous injection, at a weight-based dose of 10 mg/kg once daily, with a maximum daily dosage not to exceed 500 mg | WMT   - Supplemental oxygen therapy - Antiviral medicines - Antibiotic agents - Immune modulators | RCT   - multicenter - open label - 14d | ②④⑥ |
| **Wu-zhong Xiong^[49]^**  ***et al.***  **2020** | ICW:22/CWM:20   - mild/ moderate COVID-19 | Xuanfei Baidu decoction on the basis of the WMT   - Oral, 200ml/time, twice daily | WMT   - Supplemental oxygen therapy - Antiviral medicines - Antibiotic agents - Immune modulators | RCT   - 7d | ⑤⑦ |
| **XueDong An^[50]^**  ***et al.***  **2021** | ICW:92/CWM:31   - mild/ moderate COVID-19 - Bronchial asthma (0.8%), coronary artery disease (0.8%), hypertension (1.6%), diabetes (18.7%), hyperlipidemia (15.4%) | Jinhua Qinggan granule on the basis of the WMT   - Oral, 5 g/sachet, 1 sachet at a time | WMT   - Antiviral therapy: oseltamivir (75 mg/tablet, orally, 1 tablet at a time, twice daily)and arbidol (100 mg/tablet, orally, 2 tablets at a time, thrice daily) - Antimicrobial therapy: penicillin, cephalosporins, floxacins, and macrolides | RCT   - 14d | ②⑤⑨ |
| **Congcong Zeng^[51]^**  ***et al.***  **2021** | ICW:30/CWM:29   - mild/ moderate COVID-19 | Maxingshigan Weijing decoction on the basis of the WMT   - Oral, 200mL, 2 times daily | WMT   - Supportive treatment - Oxygen therapy - Broad-spectrum antibiotics - Antivirals | RCT   - open label - 14d | ②④⑮ |
| **Shuang Zhou^[52]^**  ***et al.***  **2021** | ICW:57/CWM:54   - severe/ critical COVID-19 | Shenhuang granule on the basis of the WMT   - Oral, with a dosing regimen of two sachets/day | WMT   - Oxygen therapy - Circulatory support - Renal replacement therapy - Blood purification treatment - Immunotherapy - Other therapeutic measures: short-term use of glucocorticoids | RCT   - multicenter - open label - 14d | ③ |
| **Wen Long^[53]^**  ***et al.***  **2020** | ICW^a^:20/ICW^b^:20/CWM:20   - severe COVID-19 | Xuebijing injection on the basis of the WMT   - Intravenous injection, 50ml XBJ injection was diluted with 100ml normal saline to150ml, twice daily | WMT   - Oxygen therapy - Circulatory support - Renal replacement therapy - Blood purification treatment - Immunotherapy - Other therapeutic measures: short-term use of glucocorticoids | RCT   - 7d | ⑪⑫⑭ |
| **Jie Zhao^[54]^**  ***et al.***  **2020** | ICW:15/CWM:24   - severe COVID-19 | Yidu-toxicity blocking lung decoction on the basis of the WMT | WMT   - Supportive treatment - Supplemental oxygen - Mechanical ventilation - Antibiotic agents - Antiviral therapy | RCT   - 29d | ⑧⑪⑮ |
| **Xiangru Xu^[55]^**  ***et al.***  **2023** | ICW:1411/CWM:1407   - mild COVID-19 - Hypertension (12.4%), diabetes mellitus (3.7%),   cerebrovascular disease (0.6%), cardiovascular disease (2.7%),  chronic pulmonary disease (1%),  chronic liver disease (0.2%), chronic kidney disease (0.1%) | Reyanning liquid on the basis of the WMT   - Oral, 20 ml, 4 times daily | WMT   - Antivirals - Antibiotics - Oral corticosteroids - Antipyretic analgesics - Antihistamines - Antitussive - Expectorant - Nasal decongestant - Antiasthmatic | RCT   - open label - 7d | ②④⑮ |
| **Ling Zhang^[56]^**  ***et al.***  **2022** | ICW:72/CWM:72   - mild/ moderate COVID-19 | Lianhua Qinwen tablets on the basis of the WMT   - Oral, 4 tablets, thrice daily | WMT   - Supportive oxygen therapy - Antivirals - Symptom management | RCT   - multicenter - open label - 14d | ① |
| **Chen Zhao^[57]^**  ***et al.***  **2021** | ICW:204/CWM:204   - mild COVID-19 | Huashi Baidu granule on the basis of the WMT   - Oral, 20g/sachet, twice daily | WMT   - Supportive treatment - Antiviral therapy: abidor capsule (0.2 g orally, thrice daily) | RCT   - open label - 7d | ②⑨⑮ |
| **Zhijian Luo^[58]^**  ***et al.* 2021** | ICW:29/CWM:28   - severe COVID-19 - Hypertension (52.6%), diabetes (14%), heart disease (5.3%), cerebral infarction (8.8%) | Xuebijing injection on the basis of the WMT   - Intravenous injection, 50ml XBJ injection was diluted with 100ml normal saline to150ml, twice daily. | WMT   - 150ml saline - Nutritional support - oxygen therapy - Antiviral therapy: interferon α -2b inhalation - Antibiotic agents - Non-invasive and invasive ventilation if necessary | RCT   - 14 d | ⑥ |
| **Jia Ke^[59]^**  ***et al.***  **2020** | ICW:81/CWM:22   - moderate COVID-19 | TCM syndrome differentiation treatment on the basis of the WMT | WMT   - Antiviral therapy: abidor capsule (0.2 g orally, thrice daily) - Antibiotics: moxifloxacin hydrochloride (tablets, 0.4 g orally, twice daily) | RCS | ⑥⑧⑪⑫ |
| **Yu Wang^[60]^**  ***et al.***  **2021** | ICW:23/CWM:32   - severe COVID-19 - Cardiovascular disease (23.6%), digestive disease (7.3%), endocrine disease (14.5%),cancer (7.3%), respiratory disease (3.6%) | Huashi Baidu granule (137 g orally, thrice daily) combined with the injections of Xiyanping (100 mg intravenous injection, thrice daily), Xuebijing (100 ml intravenous injection, thrice daily) and Shengmai (60 ml intravenous injection, twice daily) on the basis of the WMT | WMT   - Antiviral therapy: abidor capsule (0.2 g orally, thrice daily), Lopinavir–Ritonavir tablets (500 mg orally, thrice daily), - Antibiotics: cefoperazone (2 g intravenous injection, thrice daily), moxifloxacin hydrochloride tablets (0.4 g orally, twice daily) - Corticosteroid therapy: methylprednisolone succinate sodium (40 mg intravenous injection, twice daily), prednisone (30 mg orally, twice daily) - Supportive therapy: oxygen inhalation - Other therapeutic measures: symptomatic treatment, and/or human intravenous immunoglobulin, and/or serum albumin, and treatment for underlying disease | RCS | ⑬⑭ |
| **XIA Wenguang^[61]^**  ***et al.***  **2022** | ICW:80/CWM:40   - moderate COVID-19 - Tuberculosis (1.7%), COPD(1.7%), hypertension (49.2%), diabetes (23.3%), CKD (2.5%),stroke (3.3%) | TCM decoction on the basis of the WMT   - Oral, 400 ml, one dose a day, divided into two doses | WMT   - Supportive treatment - Effective oxygen therapy - Antiviral therapy: α-interferon atomized inhalation (5 million U in 2 mL sterilized water, twice daily), Arbidol Hydrochloride (0.2 g, three times a day), and intravenously injected Ribavirin (0.6 g in 250 mL of 0.9% sodium chloride, twice daily) - Antibacterial treatment: antibacterial drugs given in a timely manner upon observation of evidence of secondary bacterial infection. | RCT   - 14d | ① |
| **Guohua Chen^[62]^**  ***et al.***  **2020** | ICW:156/CWM:156   - 84.9% severe COVID-19, 15.1% critical COVID-19 - COPD(2.9%), hypertension (31.4%), cardiovascular disease (8%), diabetes (14.2%), malignancy (1.8%), cerebrovascular disease (5.7%), chronic kidney disease (0.6%), chronic liver disease(1.2%) | Mahuang Liu Jun decoction on the basis of the WMT   - Oral, 200 mL each time/twice daily | WMT   - Supplemental oxygen - Antivirals (interferon α -2b or ribavirin) - Antibiotics (moxifloxacin, cefoperazone sodium, sulbactam sodium) - Critical patients were treated with non-invasive mechanical ventilation, invasive mechanical ventilation, and extracorporeal mem-brane oxygenation to support life. | RCS | ③ |
| **Jun Feng^[63]^ *et al.***  **2021** | ICW:33/CWM:85   - critical COVID-19 - Hypertension (44%), coronary heart disease (17.7%), chronic pulmonary disease (7.5%), diabetes (21.2%), cerebrovascular disease (5.7%), chronic kidney disease (2.4%), malignancy (0.8%) | Shenhuang granule on the basis of the WMT   - Oral, two sachets/day | WMT   - Oxygen therapy - Circulatory support - Renal replacement therapy - Blood purification treatment - Immunotherapy - Other therapeutic measures: short-term use of glucocorticoids | RCS | ③ |
| **Yuanyuan Wang^[64]^**  ***et al.***  **2021** | ICW:43/CWM:43   - severe COVID-19 - COPD (1%), hypertension (41%), coronary heart disease (19%), diabetes (16%), malignancy (2%), cerebrovascular disease (2%), immunodeficiency (1%). | CHM granules on the basis of the WMT   - Oral, twice daily | WMT   - General treatment - Supplemental oxygen - Mechanical ventilation - Antibiotic agents - Antiviral therapy | RCS | ①③⑧ |
| **Jing Zhang^[65]^**  ***et al.***  **2022** | ICW:117/CWM:117   - mild COVID-19 | Shufeng Jiedu capsule on the basis of the WMT   - Oral, 0.52 g per capsule, 4 capsules at a time, thrice daily | WMT   - General treatment - Supplemental oxygen - Mechanical ventilation - Antibiotic agents - Antiviral therapy | RCT   - open label - 7d | ④⑤ |
| **Jianping Zhang^[66]^**  ***et al.***  **2023** | ICW:91/CWM:90   - mild COVID-19 | Maizao decoction and Liushen pill on the basis of the WMT   - Liushen pill, Oral, three sublingual pills and seven oral pills at a time, three times a day; Maizao decoction, oral, 200 ml, one dose a day, divided into two doses | WMT   - General treatment - Supplemental oxygen - Mechanical ventilation - Antibiotic agents - Antiviral therapy | RCT   - 7d | ⑨⑭ |
| **Hai-Bo Hu^[67]^**  ***et al.***  **2021** | ICW:47/CWM:34   - severe COVID-19 - Diabetes (19.8%), hypertension (29.6%), coronary artery disease (23.5%), chronic kidney disease(1.2%) | He-Jie-Shen-Shi granule on the basis of the WMT | WMT   - General treatment - Supplemental oxygen - Mechanical ventilation - Antibiotic agents - Antiviral therapy | RCS | ④⑥⑧⑪⑬⑭ |

Abbreviation: COPD: Chronic obstructive pulmonary disease; ICW: integrated Chinese herbal medicine and western medicine therapy; CWM: conventional western medicine; WMT: western medicine therapy; TCM: traditional Chinese medicine; COVID-19: coronavirus disease 2019; RCT: randomized controlled trial; RCS: retrospective cohort study; PCS: prospective cohort study

Outcome indicator: ①improvement rate of chest CT ②severe conversion rate ③mortality rate ④negativity time of nucleic acid ⑤rate of fever reduction ⑥time to reduction of fever ⑦improvement rate of cough ⑧time to improvement of cough ⑨improvement rate of breathless ⑩improvement rate of fatigue ⑪white blood cell count ⑫lymphocytes count ⑬neutrophil count ⑭C-reactive protein count ⑮length of hospital stays
