# Supplementary material for: Effect of Chinese herbal medicine (CHM) as an adjunctive therapy in distinct stages of patients with COVID-19: A systematic review and meta-analysis
Source: PLoS One. 2025 Feb 13;20(2):e0318892. doi: 10.1371/journal.pone.0318892 (PMC11825027; doi:10.1371/journal.pone.0318892)
Supplement: S5 Table — (DOCX) [file pone.0318892.s008.docx]

**Supplementary Table S5. Bias risk assessment results of included retrospective studies**

| **Study** | **NOS** | | | | | | | | **Total score** |
| --- | --- | --- | --- | --- | --- | --- | --- | --- | --- |
|  | ① | ② | ③ | ④ | ⑤ | ⑥ | ⑦ | ⑧ |  |
| **QIN Lingxi, *et al.*2021** | 1 | 1 | 1 | 0 | 1 | 1 | 1 | 1 | 7 |
| **HUANG Donghui, *et al.*2021** | 1 | 1 | 1 | 1 | 1 | 1 | 1 | 1 | 8 |
| **LUO Zhihui, *et al.*2021** | 1 | 1 | 1 | 1 | 1 | 1 | 1 | 1 | 8 |
| **ZENG Xianhong, *et al.*2020** | 1 | 1 | 1 | 0 | 1 | 1 | 1 | 1 | 7 |
| **CHEN Fei, *et al.*2022** | 1 | 1 | 1 | 0 | 1 | 1 | 1 | 1 | 7 |
| **YANG Qian, *et al.*2020** | 1 | 1 | 1 | 0 | 1 | 1 | 1 | 1 | 7 |
| **JI Dan, *et al.*2020** | 1 | 1 | 1 | 0 | 1 | 1 | 1 | 1 | 7 |
| **YAO Kaitao, *et al.*2020** | 1 | 1 | 1 | 1 | 1 | 1 | 1 | 1 | 8 |
| **Ya-Hui Li, *et al.*2021** | 1 | 1 | 1 | 1 | 1 | 1 | 1 | 1 | 8 |
| **Chao Qun Huang, *et al.*2023** | 1 | 1 | 1 | 0 | 1 | 1 | 1 | 0 | 6 |
| **Nannan Shi, *et al.*2021** | 1 | 1 | 1 | 1 | 2 | 1 | 1 | 1 | 9 |
| **YAN Xiangyong, *et al.*2021** | 1 | 1 | 1 | 1 | 2 | 1 | 1 | 1 | 9 |
| **Fei He, *et al.*2022** | 1 | 1 | 1 | 0 | 1 | 1 | 1 | 1 | 7 |
| **QIAN Yu-jun, *et al.* 2020** | 1 | 1 | 1 | 0 | 1 | 1 | 1 | 1 | 7 |
| **Hong-Ling Li, *et al.*2020** | 1 | 1 | 1 | 0 | 1 | 1 | 1 | 1 | 7 |
| **Zhi-Dan Lu, *et al.*2022** | 1 | 1 | 1 | 0 | 1 | 1 | 1 | 1 | 7 |
| **Yu Wang, *et al.*2021** | 1 | 1 | 1 | 0 | 1 | 1 | 1 | 1 | 7 |
| **Guohua Chen, *et al.*2020** | 1 | 1 | 1 | 0 | 1 | 1 | 1 | 1 | 7 |
| **Jun Feng, *et al.*2021** | 1 | 1 | 1 | 0 | 1 | 1 | 1 | 1 | 7 |
| **Yuanyuan Wang, *et al.*2021** | 1 | 1 | 1 | 1 | 2 | 1 | 1 | 1 | 9 |
| **Hai-Bo Hu, *et al.*2021** | 1 | 1 | 1 | 1 | 1 | 1 | 1 | 1 | 8 |
| **Jia Ke, *et al.*2020** | 1 | 1 | 1 | 0 | 1 | 1 | 1 | 1 | 7 |

①②③④ selection of participants; ⑤ comparability between study groups; ⑥⑦⑧ measurement of exposure factors or results.
