# Supplementary material for: Effect of Chinese herbal medicine (CHM) as an adjunctive therapy in distinct stages of patients with COVID-19: A systematic review and meta-analysis
Source: PLoS One. 2025 Feb 13;20(2):e0318892. doi: 10.1371/journal.pone.0318892 (PMC11825027; doi:10.1371/journal.pone.0318892)
Supplement: S6 Table — (DOCX) [file pone.0318892.s009.docx]

**Supplementary Table S6. Components of TCM prescriptions in the included studies**

| **study** | **Chinese herbal medicine** | **Components (Chinese name and Latin name were used in the bracket)** |
| --- | --- | --- |
| **QIN Lingxi*, et al.***  **2021** | TCM decoction, Lianhua Qingwen granule, and Xuebijing injection | Fructus Forsythiae Suspensae (Lianqiao), Flos Lonicerae (Jinyinhua), Herba Ephedrae (Mahuang), Semen Armeniacae Amarum (Kuxingren), Gypsum Fibrosum (Shigao), Radix Isatidis (Banlangen), Rhizoma Dryopteris Crassirhizomae (Mianma Guanzhong), Herba Houttuyniae (Yuxingcao), Herba Pogostemonis(Huoxiang), Radix Et Rhizoma Rhei (Dahuang), Herba Rhodiolae(Hongjingtian), Radix Glycyrrhizae (Gancao), Carthamus tinctorius(Honghua), Radix Paeoniae Rubra(Chishao), Szechuan Lovage Rhizome (Chuanxiong), Salvia miltiorrhiza (Danshen), Angelica sinensis(Danggui) |
| **HUANG Donghui*, et al.***  **2021** | No. 3 formula of Gengzi | Cornus officinalis (Shanzhuyu) 45g, FossiliaOssisMastrodi (Shenglonggu) 30g, Ostreagigastnunb (Shengmuli) 30g, Radix LateralisPraeparatus Aconiti Carmichaeli (Fuzi) 20g, Radix Ginseng (Renshen)20g, Magnet powder (Cishifeng) 20g，Rhizoma Zingiberisa (Ganjiang) 15g, Radix Glycyrrhizae (Zhi Gancao) 15g, Rhizoma Atractylodis Macrocephalae (Baizhu) 15g, Scierotium Poriae Cocos(Fuling) 15 g，Acori Rhizoma (Shichangpu) 12 g, Fructus Schisandrae Chinensis (Wuweizi) 9 g |
| **LUO Zhihui*, et al.***  **2021** | Sanxiao decoction | Fructus Tsaoko (Caoguo) 10g,Semen Arecae (Binglang) 10g, Cortex Magnoliae Officinalis (Houpu) 10g, Radix Scutellariae Baicalensis (Huangqin) 10g, Radix Albus Paeoniae Lactiflorae(Baishaoyao) 10g  , Rhizoma Anemarrhenae Aspheloidis (Zhimu) 10g, Radix Glycyrrhizae (Gancao) 10g，Radix EtRhizoma Rhei (Dahuang) 6g，Bombyx Batryticatus (Jiangcan) 10g，Periostracum Cicadae (Chantui) 10g，Microcos paniculate (Buzhaye)10g，Bombax ceiba (Mumianhua) 10g, Achyranthes bidentata Blume (Niuxi) 10g, Ficus carica (Wuhuaguo) 10g |
| **YE Ling*, et al.***  **2021** | Shengjiang powder  granule | Bombyx Batryticatus (Jiangcan) 10g, Periostracum Cicadae (Chantui) 4g, Rhizoma Curcumae Longae (Jianghuang) 8g, Radix EtRhizoma Rhei (Dahuang) 4g, Radix Astragali Membranacei (Huangqi) 30g，Rhizoma Atractylodis Macrocephalae (Baizhu) 15g, Radix Ledebouriellae Divaricatae (Fangfeng) 9g，Folium Isatidis (Daqingye) 15g, Herba Houttuyniae (Yuxingcao) 15g, Herba Pogostemonis (Huoxiang) 9g |
| **YU Ping*, et al.***  **2020** | Lianhua Qingwen capsule/ granule | Fructus Forsythiae Suspensae (Lianqiao), Flos Lonicerae (Jinyinhua), Herba Ephedrae (Mahuang), Semen Armeniacae Amarum (Kuxingren), Gypsum Fibrosum (Shigao), Radix Isatidis (Banlangen), Rhizoma Dryopteris Crassirhizomae (Mianma Guanzhong), Herba Houttuyniae (Yuxingcao), Herba Pogostemonis (Huoxiang), Radix Et Rhizoma Rhei (Dahuang), Herba Rhodiolae (Hongjingtian), Radix Glycyrrhizae (Gancao) |
| **ZENG Xianhong*, et al.***  **2020** | Qingfei Paidu decoction | Herba Pogostemonis (Huoxiang) 9g, Gypsum Fibrosum (Sheng Shi Gao) 30g, Radix Bupleuri (Chaihu) 16g, Rhizoma Pinelliae (Jiangbanxia) 9g, Herba Ephedrae (Ma Huang) 9g, Asarum (Xi Xin)3g, Radix Asteris Tatarici (Ziwan) 9g, Rhizoma Alismatis (Zexie) 9g, Sclerotium Polypori Umbellati (Zhuling) 9g, Dioscoreae Rhizoma (Shanyao) 12g, Fresh Rhizoma Zingiberis (Shengjiang) 9g, Semen Armeniacae Amarum (Kuxingren) 9g, Pericarpium Citri Reticulatae (Chenpi) 6g, Rhizoma Atractylodis Macrocephalae (Baizhu) 9g, Ramulus Cinnamomi Cassiae (Guizhi) 9g, Rhizoma Belamcandae (Shegan) 9g, Scierotium Poriae Cocos (Fuling) 15g, Fructus Citri Seu Ponciri Immaturus (Zhishi) 6g, Radix Scutellariae Baicalensis (Huangqin) 6g, Flos Farfarae (Kuandonghua) 9g, Radix Glycyrrhizae (Zhi Gancao) 6g |
| **YANG Mingbo*, et al.***  **2020** | Reyanning injection | Herba Taraxaci (Pugongying), Rhizoma Polygoni Cuspidati (Huzhang), Herba Patriniae (Baijiangcao), Herba Scutellariae Barbatae (Banzhilian) |
| **WANG Lin*, et al.***  **2020** | Shengmai powder combined with Shenling Baizhu powder decoction | Pseudostellariae Radix (Taizishen) 30g, Tuber OphiopogonisJaponici (Maidong)15g, Fructus Schisandrae Chinensis (Wuweizi) 15g，Scierotium Poriae Cocos (Fuling) 15g，Rhizoma Atractylodis Macrocephalae (Baizhu) 15g, SemenDolichorisAlbum (Baibiandou) 15g，Pericarpium Citri Reticulatae (Chenpi) 10g, Dioscoreae Rhizoma (Shanyao) 20g，Platycodi Radix (Jiegeng) 10g，Cortex Magnoliae Officinalis (Houpu) 10g, Rehmannia glutinosa (Shengdihuang) 15g |
| **Wen Long*, et al.***  **2020** | Xuebijing injection | Carthamus tinctorius (Honghua), Radix Paeoniae Rubra (Chishao), Szechuan Lovage Rhizome (Chuanxiong), Salvia miltiorrhiza (Danshen), Angelica sinensis (Danggui) |
| **WANG Yuan*, et al.***  **2022** | TCM syndrome differentiation treatment | Stagnant evil of Shaoyang: Xiaochaihu decoction: Radix Bupleuri (Chaihu)，Radix Scutellariae Baicalensis (Huangqin)，Rhizoma Pinelliae (banxia), Radix Ginseng (Renshen)，Rhizoma Zingiberisa (Shengjiang)，Radix Glycyrrhizae (Zhi Gancao)，Fructus Zizyphi Jujubae (Dazao)  Pathogenic volt membrane: Dayuan decoction ；Fructus Tsaoko (Caoguo)，Semen Arecae (Binglang)，Cortex Magnoliae Officinalis (Houpu)，Radix Scutellariae Baicalensis (Huangqin), Radix Albus Paeoniae Lactiflorae (Baishaoyao), Rhizoma Anemarrhenae Aspheloidis (Zhimu), Radix Glycyrrhizae (Gancao)  Damp and heat accumulate in the lungs: Huangqin Huashi decoction；Radix Scutellariae Baicalensis (Huangqin), Scierotium Poriae Cocos(Fuling), Fructus Ammomi Rotundus (Baidoukou), Sclerotium Polypori Umbellati (Zhuling), talcum(Huashi), Pericarpium Arecae (Dafupi), Tetrapanax papyriferus (Tongcao)  Epidemic virus blocked the lung: Maxingshigan decoction combined with Xuanbai Chengqi decoction and Dayuan decoction: Herba Ephedrae (Ma Huang), Semen Armeniacae Amarum (Xing Ren), Gypsum Fibrosum (Shigao), Radix Glycyrrhizae (Gancao), Radix Et Rhizoma Rhei (Dahuang), Fructus Trichosanthis (Gualou), Fructus Tsaoko (Caoguo)，Semen Arecae (Binglang)，Cortex Magnoliae Officinalis (Houpu)，Radix Scutellariae Baicalensis (Huangqin), Radix Albus Paeoniae Lactiflorae (Baishaoyao), Rhizoma Anemarrhenae Aspheloidis (Zhimu)  Lack of qi and Yin: Shengmai powder: Radix Ginseng (Renshen), Tuber OphiopogonisJaponici (Maidong), Fructus Schisandrae Chinensis (Wuweizi)  Burning of qi ying: Qing ying decoction: Comu Buball (Shuiniujiao), Rehmannia glutinosa (Shengdihuang), scrophularia (Yuanshen), Folium Phyllostachys (Zhuye), Tuber Ophiopogonis Japonici (Maidong), Salvia Miltiorrhiza (Danshen), Rhizoma Coptidis (Huanglian), Fructus Forsythiae Suspensae (Lianqiao), Flos Lonicerae (Jinyinhua) |
| **CHEN Fei*, et al.***  **2022** | TCM syndrome differentiation treatment | Dampness toxin stagnation of lung: Herba Ephedrae (Sheng Ma Huang) 6g, Semen Armeniacae Amarum (Kuxingren) 15g, Gypsum Fibrosum (Sheng Shi Gao)30g, Semen Coicis (Yiyiren) 30g, Rhizoma Atractylodis (Cangzhu) 10g, Herba Pogostemonis (Huoxiang) 15g, Artemisiae Apiaceae seu Annuae Herba (Qinghao) 12g, Rhizoma Polygoni Cuspidati (Huzhang) 20g, Herba Verbenae (Mabiancao)30g, Rhizoma Phragmitis (Lugen) 30g, Semen Lepidii (Tinglizi) 15g, Exocarpium Citri Grandis (Huajuhong) 15g, Radix Glycyrrhizae (Sheng Gancao) 10g  Cold dampness obstructive lung: Rhizoma Atractylodis (Cangzhu) 15g, Pericarpium Citri Reticulatae (Chenpi) 10g, Cortex Magnoliae Officinalis(Houpu) 10g, Herba Pogostemonis (Huoxiang)10g, Fructus Tsaoko (Caoguo) 6g, Ephedrae Herba (Sheng Ma Huang) 6g, Notopterygium incisuma (Qianghuo)10g, Fresh Rhizoma Zingiberis (Shengjiang) 10g, Semen Arecae (Binglang) 10g |
| **YANG Qian*, et al.***  **2020** | TCM syndrome differentiation treatment | Cold dampness stagnation of lung：Rhizoma Atractylodis (Cangzhu) 15 g, Pericarpium Citri Reticulatae (Chenpi) 10 g, Cortex Magnoliae Officinalis (Houpu) 10 g, Herba Pogostemonis (Huoxiang) 10g, Fructus Tsaoko (Caoguo) 6 g, Herba Ephedrae (Sheng Ma Huang) 6g, Notopterygium incisuma (Qianghuo) 10g, Fresh Rhizoma Zingiberis(Shengjiang) 10g, Semen Arecae (Binglang) 10g  Epidemic virus blocked the lung: Semen Armeniacae Amarum (Xingren) 10g, Gypsum Fibrosum (Shigao)30g, Fructus Trichosanthis (Gualou) 30g, Radix Et Rhizoma Rhei (Dahuang) 6 g, Ephedrae Herba (Sheng Ma Huang)6g, Ephedrae Herba (Zhi Ma Huang) 6g, Lepidii seu Descurainiae Semen (Ting Li Zi) 10g, Persicae Semen (Taoren)10 g, Fructus Tsaoko (Caoguo) 6g, Semen Arecae (Binglang)10 g, Rhizoma Atractylodis (Cangzhu) 10g |
| **JI Dan*, et al.***  **2020** | formula of novel coronavirus decoction | Herba Ephedrae (Ma Huang) 6g, Semen Armeniacae Amarum (Xing Ren) 6g, Fructus Tsaoko (Caoguo) 10g, Semen Arecae(Binglang) 6 g, Periostracum Cicadae(Chantui) 9 g, Fructus Forsythiae Suspensae (Lianqiao) 10 g,Rhizoma Atractylodis (Cangzhu) 10 g，Platycodi Radix (Jiegeng) 10 g, Radix Scutellariae Baicalensis (Huangqin) 10 g, Fructus Arctii (Niubangzi) 9 g，Radix Glycyrrhizae (Sheng Gancao) 6 g |
| **YAO Kaitao*, et al.***  **2020** | Lianhua Qingwen granule | Fructus Forsythiae Suspensae (Lianqiao), Flos Lonicerae (Jinyinhua), Herba Ephedrae (Mahuang), Semen Armeniacae Amarum (Kuxingren), Gypsum Fibrosum (Shigao), Radix Isatidis (Banlangen), Rhizoma Dryopteris Crassirhizomae (Mianma Guanzhong), Herba Houttuyniae (Yuxingcao), Herba Pogostemonis(Huoxiang), Radix Et Rhizoma Rhei (Dahuang), Herba Rhodiolae(Hongjingtian), Radix Glycyrrhizae (Gancao) |
| **Chen Zhao*, et al.***  **2021** | Huashi Baidu granule | Ephedrae Herba (Ma Huang) 6 g, Semen Armeniacae Amarum (Kuxingren) 9 g, Gypsum Fibrosum (Sheng Shi Gao)15 g, Radix Glycyrrhizae (Gancao) 3 g, Herba Pogostemonis (Huoxiang) 10 g, Cortex Magnoliae Officinalis (Houpu) 10 g, Rhizoma Atractylodis (Cangzhu) 15 g, Fructus Tsaoko (Caoguo) 10 g, Rhizoma Pinelliae (Fabanxia) 9 g, Scierotium Poriae Cocos (Fuling) 15 g, Radix Et Rhizoma Rhei (Sheng Dahuang) 5 g, Astragalus membranaceus (Shenghuangqi) 10 g, Lepidii seu Descurainiae Semen (Ting Li Zi) 10 g, Radix Paeoniae Rubra(Chishao) 10 g |
| **Jia Liu*, et al.***  **2021** | Huashi Baidu granule | Ephedrae Herba (Ma Huang) 6g, Semen Armeniacae Amarum (Kuxingren) 9g, Gypsum Fibrosum (Sheng Shi Gao) 15g, Radix Glycyrrhizae (Gancao) 3g, Herba Pogostemonis (Huoxiang) 10 g, Cortex Magnoliae Officinalis (Houpu) 10g, Rhizoma Atractylodis (Cangzhu) 15g, Fructus Tsaoko (Caoguo) 10 g, Rhizoma Pinelliae (Fabanxia) 9g, Scierotium Poriae Cocos (Fuling)15g, Radix Et Rhizoma Rhei (Sheng Dahuang)5g, Astragalus membranaceus (Shenghuangqi)10g, Lepidii seu Descurainiae Semen (Ting Li Zi)10g, Radix Paeoniae Rubra(Chishao)10g |
| **Nannan Shi*, et al.***  **2021** | Huashi Baidu granule | Ephedrae Herba (Ma Huang)6g, Semen Armeniacae Amarum (Kuxingren)9g, Gypsum Fibrosum (Sheng Shi Gao)15 g, Radix Glycyrrhizae (Gancao)3g, Herba Pogostemonis (Huoxiang)10g, Cortex Magnoliae Officinalis (Houpu)10 g, Rhizoma Atractylodis (Cangzhu)15g, Fructus Tsaoko (Caoguo)10g, Rhizoma Pinelliae (Fabanxia)9 g, Scierotium Poriae Cocos (Fuling)15g, Radix Et Rhizoma Rhei (Sheng Dahuang)5g, Astragalus membranaceus (Shenghuangqi)10g, Lepidii seu Descurainiae Semen (Ting Li Zi)10g, Radix Paeoniae Rubra (Chishao)10g |
| **Ling Zhang*, et al.***  **2022** | Lianhua Qingke Tablet | Ephedrae Herba (Ma Huang), Cortex Mori Radicis (Sangbaipi), Gypsum Fibrosum (Sheng Shi Gao), Radix Scutellariae Baicalensis (Huangqin), Semen Armeniacae Amarum (Kuxingren), Fructus Forsythiae Suspensae (Lianqiao), Rhizoma Pinelliae (Jiangbanxia), Thunberg Fritillary Bulb (Zhebeimu), Radix Peucedani (Qianhu), Fructus arctii (Niubangzi), Flos Lonicerae (Jinyinhua), Radix Et Rhizoma Rhei (Sheng Dahuang), Pericarpium Citri Reticulatae (Chenpi) |
| **Xiangru Xu*, et al.***  **2023** | Reyanning injection | Herba Taraxaci (Pugongying), Rhizoma Polygoni Cuspidati (Huzhang), Herba Patriniae (Baijiangcao), Herba Scutellariae Barbatae (Banzhilian) |
| **Xin-Yi Zhang*, et al.***  **2021** | Xiyanping injection | Andrographis paniculate (Chuanxinlian) |
| **Wu-zhong Xiong*, et al.***  **2020** | Xuanfei Baidu decoction | Herba Ephedrae (Ma Huang) 8g, Semen Armeniacae Amarum (Xing Ren) 15g, Gypsum Fibrosum (Sheng Shi Gao) 30g, rhizoma atractylodis (Cang Zhu) 10g, Semen Coicis (Yiyiren) 30g, Herba Pogostemonis (Huoxiang)15g, Rhizoma Polygoni Cuspidati (Huzhang) 20g, Lepidii seu Descurainiae Semen (Ting Li Zi) 15g, Herba Verbenae (Mabiancao)30g, Phragmitis Rhizoma (Lu Gen) 30g, Artemisiae, Annuae Herba (Qing Hao) 25g, Citri Grandis Rubrum Exocarpium, (Ju Hong) 20g, Glycyrrhizae Radix et Rhizoma (Sheng Gan Cao) 10g. |
| **XueDong An*, et al.***  **2021** | Jinhua Qinggan granule | Flos Lonicerae (Jinyinhua), Gypsum Fibrosum (Shigao), Herba Ephedrae (Mahuang), Semen Armeniacae Amarum (Kuxingren), Radix Scutellariae Baicalensis (Huangqin), Fructus Forsythiae Suspensae (Lianqiao), Bulbus Fritillariae Thunbergii (Zhebeimu), Rhizoma Anemarrhenae Aspheloidis (Zhimu), Fructus Arctii (Niubangzi), Artemisiae Apiaceae seu Annuae Herba (Qinghao), Herba Menthae Haplocalycis (Bohe), Radix Glycyrrhizae (Gancao) |
| **Zhijian Luo*, et al.***  **2021** | Xuebijing injection | Carthamus tinctorius (Honghua), Radix Paeoniae Rubra (Chishao), Szechuan Lovage Rhizome (Chuanxiong), Salvia miltiorrhiza (Dansheng), Angelica sinensis (Danggui) |
| **Congcong Zeng*, et al.***  **2021** | Maxingshigan Weijing decoction | Herba Ephedrae (Ma Huang)10g, Semen Armeniacae Amarum (Xing Ren)10g, Gypsum Fibrosum (Shigao) 45g, Rhizoma phragmitis (Lugen)30g, Persicae Semen (Taoren)20g, winter melon kernel (Dongguaren) 20g, Trichosanthes Kirilowii Maxim (Gualou)30g, Pericarpium Citri Reticulatae (Chenpi)12g, Rhizoma Pinelliae (Jiangbanxia)12g, caulis bambusae in taeniis (Zhuru)12g, Semen lepidii (Tingliz) 30g, Acori Rhizoma (Shichangpu)15g, curcuma zedoary (ezhu)10g, Radix Glycyrrhizae (Gancao)5g |
| **Jun Feng*, et al.***  **2021** | Shenhuang granule | Radix Ginseng (Renshen) 50g, Radix Et Rhizoma Rhei (Dahuang)40 g, Sargentodoxa cuneata stem (Hongteng)30g, Herba Taraxaci (Pugongying)30g, Radix LateralisPraeparatus Aconiti Carmichaeli (Fuzi)50 g, Whitmania pigra Whitman (Shuizhi) 6g |
| **Jie Zhao*, et al.***  **2020** | Yidu-toxicity blocking lung decoction | Semen Armeniacae Amarum (Kuxingren), Gypsum Fibrosum (Sheng Shigao), Trichosanthes Kirilowii Maxim (Gualou), Radix Et Rhizoma Rhei (Sheng Dahuang), Herba Ephedrae (Sheng Ma Huang), Herba Ephedrae (Zhi Ma Huang), Semen lepidii (Tingliz), Persicae Semen (Taoren), Fructus Tsaoko (Caoguo), Semen Arecae (Binglang), Rhizoma Atractylodis (Cangzhu) |
| **Jia Ke*, et al.***  **2020** | TCM syndrome differentiation treatment | There were 29 cases of Xiaochaihu Decoction combined with Dayuan decoction, 6 cases of Yinqiao Powder combined with Huoxiang Zhengqi Powder, 5 cases of Sanao decoction combined with Xiaoqiangbei Decoction, 2 cases of Yinqiao powder and Sangjuyin, 4 cases of Erchen decoction combined with Sanzi Yangqin, 13 cases of Yinqiao powder combined with Zhisu Powder, 8 cases of Ma Xingshigan Decoction and Gualou Xiebai Banxia Decoction, 5 cases of Shashen Maidong Decoction, 3 cases of Ma Xingshigan Decoction combined with Xiaochaihu decoction, There were 3 cases of Yinqiao powder combined with small bupleurum, 2 cases of Maxinghuigan decoction combined with Caoguo Zhimu decoction, and 1 case of small bupleurum decoction combined with hopi Puxialing decoction. |
| **Ya-Hui Li*, et al.***  **2021** | Jinbei Oral Liquid | Radix Astragali (Huangqi), Codonopsis pilosula (Dangshen), Radix Glehniae (Beishashen), Salvia miltiorrhiza (Danshen), Angelica sinensis(Danggui), Szechuan Lovage Rhizome (Chuanxiong), Flos Lonicerae (Jinyinhua), Fructus Forsythiae Suspensae (Lianqiao), Radix Scutellariae Baicalensis (Huangqin), FaRhizoma Pinelliae (Fabanxia), Bulbus Fritillariae Thunbergii (Zhebeimu), Radix Glycyrrhizae (Gancao) |
| **Yue Zhang*, et al.***  **2023** | Mobile Cabin Hospital (No.1/ No.2 /No.4)granule | No.1 granule consisted of Radix Bupleuri (Chaihu)15g, Radix Scutellariae Baicalensis (Huangqin) 10g, JiangRhizoma Pinelliae (Fabanxia) 10g, Fresh Rhizoma Zingiberis (Shengjiang) 10g,  Fructus Zizyphi(Dazao) 5g, Codonopsis pilosula(Dangshen) 8g, Radix Glycyrrhizae (Sheng Gancao) 5g, Ramulus Cinnamomi Cassiae (Guizhi) 10 g, Radix Albus Paeoniae Lactiflorae(Baishaoyao) 10 g, Radix Stemonae(Baibu) 10g; No.2 granule consisted of Trichosanthes Kirilowii Maxim (Gualou) 10g, JiangRhizoma Pinelliae (Fabanxia) 15g, Radix Scutellariae Baicalensis (Huangqin) 10g, Radix Stemonae(Baibu)10g, Radix Peucedani(Qianhu) 10g, Cynanchum stauntoni(Baiqian) 10g, Radix Glycyrrhizae (Sheng Gancao) 10g, Platycodi Radix (Jiegeng) 10g, Fineleaf Schizonepeta Herb（Jingjie) 10g, Radix Asteris Tatarici (Ziwan) 10g, Pericarpium Citri Reticulatae (Chenpi) 10 g; No.2 granule consisted of Angelica sinensis(Danggui) 10g, Radix Albus Paeoniae Lactiflorae(Baishaoyao) 10g, Radix Bupleuri (Chaihu) 10g, Scierotium Poriae Cocos (Fuling) 15g, Rhizoma Atractylodis Macrocephalae (Baizhu) 10g, Radix Stemonae(Baibu) 12g, Cortex albiziae(Hehuanpi) 30g, Szechuan Lovage Rhizome (Chuanxiong) 10g, Semen Ziziphi Spinosae (Suanzaoren) 15g, Rhizoma Anemarrhenae Aspheloidis (Zhimu) 10 g, Radix Glycyrrhizae (Sheng Gancao) 5g |
| **Can Duan*, et al.***  **2020** | Jinhua Qinggan granule | Flos Lonicerae (Jinyinhua), Gypsum Fibrosum (Shigao), Herba Ephedrae (Mahuang), Semen Armeniacae Amarum (Kuxingren), Radix Scutellariae Baicalensis (Huangqin), Fructus Forsythiae Suspensae (Lianqiao), Bulbus Fritillariae Thunbergii (Zhebeimu), Rhizoma Anemarrhenae Aspheloidis (Zhimu), Fructus Arctii (Niubangzi), Artemisiae Apiaceae seu Annuae Herba (Qinghao), Herba Menthae Haplocalycis (Bohe), Radix Glycyrrhizae (Gancao) |
| **Fei-Fei Lin*, et al.***  **2020** | Xuanefei Qingre decoction | Herba Ephedrae (Mahuang) 9g, Semen Armeniacae Amarum (Kuxingren) 12g, Gypsum Fibrosum (Shigao) 30g, Radix Glycyrrhizae (Gancao) 6g, Persicae Semen (Taoren) 30g, Winter melon kernel (Dongguaren) 30g, Rhizoma phragmitis (Lugen) 30g, Semen Coicis (Yiyiren) 30g, Platycodi Radix (Jiegeng) 9g, FaRhizoma Pinelliae (Fabanxia) 12g, Allium macrostemon Bunge(Xiebai) 12g, Fructus Tsaoko (Caoguo) 6g, Herba Pogostemonis(Huoxiang) 10g |
| **Chao Qun Huang*, et al.***  **2023** | TCM syndrome differentiation treatment | Qufei Paidu Decoction, Hoppu Xialing Decoction, Erchen Decoction, Sangju Decoction, or "No. 1 prescription", "No. 2 (Qingfei Paidu Decoction)", "No. 3 (Xuanfei Baidu prescription)" |
| **YAN Xiangyong*, et al.***  **2021** | Xuanefei Qingre decoction | Herba Ephedrae (Mahuang) 9g, Semen Armeniacae Amarum (Kuxingren) 12g, Gypsum Fibrosum (Shigao) 30g, Radix Glycyrrhizae (Gancao) 6g, Persicae Semen (Taoren) 30g, Winter melon kernel (Dongguaren) 30g, Rhizoma phragmitis (Lugen) 30g, Semen Coicis (Yiyiren) 30g, Platycodi Radix (Jiegeng) 9g, FaRhizoma Pinelliae (Fabanxia) 12g, Allium macrostemon Bunge(Xiebai) 12g, Fructus Tsaoko (Caoguo) 6g, Herba Pogostemonis(Huoxiang) 10g |
| **Fei He*, et al.***  **2022** | Huashi Jiedu decoction | Herba Ephedrae (Mahuang) 9g, Semen Armeniacae Amarum (Kuxingren) 10g, Gypsum Fibrosum (Shigao) 20g, Radix Scutellariae Baicalensis (Huangqin) 10g, Rhizoma Dryopteris Crassirhizomae (Guanzhong) 10g, Rhizoma Belamcandae (Shegan) 10g, Rhizoma Atractylodis (Cangzhu) 10g, Herba Pogostemonis (Huoxiang) 10g, Pericarpium Citri Reticulatae (Chenpi) 10g, Officinal Magnolia Bark(Houpo) 10g, Radix Glycyrrhizae (Gancao) 6g, Rhizoma Zingiberisa (Ganjiang) 6g, Lepidii seu Descurainiae Semen (Tinglizi) 10g, lumbricus (Dilong) 15g, Trichosanthes Kirilowii Maxim (Gualou) 10g |
| **QIAN Yu-jun*, et al.***  **2020** | Self-made TCM decotion | Radix Ginseng Rubra(Hongshen) 20g, Rhizoma Zingiberisa (Ganjiang) 45g, Ramulus Cinnamomi Cassiae (Guizhi) 40g, FaRhizoma Pinelliae (Fabanxia) 40g, Scierotium Poriae Cocos (Fuling) 50g, Radix Paeoniae Rubra(Chishao) 40g, Rhizoma Alismatis (Zexie) 30g, Radix Glycyrrhizae (Gancao) 30g, Rhizoma Zingiberisa (Ganjiang) 20g, Fructus Tsaoko (Caoguo) 20g, FossiliaOssisMastrodi (Shenglonggu) 20g, Herba Ephedrae (Mahuang) 20g, Rhizoma Atractylodis (Cangzhu) 30g, Semen Armeniacae Amarum (Kuxingren) 10g, Fructus Schisandrae Chinensis (Wuweizi) 10g |
| **Hong-Ling Li*, et al.***  **2020** | Self-made TCM decoction | Radix Bupleuri (Chaihu) 18g, Radix Scutellariae Baicalensis (Huangqin) 15g, Rhizoma Zingiberisa (Ganjiang) 8g,  Fructus Zizyphi(Dazao) 10g, FaRhizoma Pinelliae (Fabanxia) 10g, Radix Glycyrrhizae (Gancao) 6g, Radix Aucklandiae(Muxiang) 10g, Radix scrophulariae(Xuanshen) 15g, Pericarpium Citri Reticulatae (Chenpi) 10g, Herba Pogostemonis(Huoxiang) 10g, Rhizoma Atractylodis Macrocephalae (Baizhu) 10g, Semen Coicis (Yiyiren) 30g, Platycodi Radix (Jiegeng) 10g, Radix Adenophorae(Nanshashen) 10g |
| **ZHOU Tong*, et al.***  **2023** | Chonglian oral liquid | Rhizoma Paridis(Chonglou), Fructus Forsythiae Suspensae (Lianqiao), Radix Scutellariae Baicalensis (Huangqin), Rhizoma Belamcandae (Shegan), Folium Isatidis (Daqingye), Radix Isatidis (Banlangen), Radix Glycyrrhizae (Gancao) |
| **Zhi-Dan Lu*, et al.***  **2022** | Chinese patent medicine or (and) Chinese medicine injection | Lianhua Qingwen capsule, Huashui Tuedu granule, Jinhua Qinggan Granule, Feilike oral Liquid, Xuebijing injection |
| **Yu Wang*, et al.***  **2021** | Chinese patent medicine or (and) Chinese medicine injection | Huashi Baidu granule combined with the injections of Xiyanping, Xuebijing and Shenmai |
| **XIA Wenguang*, et al.***  **2022** | TCM syndrome differentiation treatment | (a). Cold-damp constraint in the lung pattern , recommended prescription: Herba Ephedrae (Sheng Ma Huang)6 g, emen Armeniacae Amarum (Kuxingren)15 g, Gypsum Fibrosum (Sheng Shi Gao)30 g,Semen Coicis (Yiyiren) 30 g,Rhizoma Atractylodis (Cangzhu)10 g, Herba Pogostemonis (Huoxiang)15 g, Artemisiae Apiaceae seu Annuae Herba (Qinghao) 12 g,Rhizoma Polygoni Cuspidati (Huzhang) 20 g, Herba Verbenae (Mabiancao)30 g, Rhizoma Phragmitis (Lugen)30 g,Fructus Gardeniae(Zhizi) 15 g, Fructus et Semen Trichosanthis(Gualou) 30g, Radix Et Rhizoma Rhei Palmati (Dahuang) 6g, Exocarpium Citri Rubrum(Juhong) 15 g, Radix Glycyrrhizae (Gancao)10 g;  (b). Damp-heat accumulation in the lung pattern, recommended prescription: Semen Arecae(Binglang) 10 g, Fructus Tsaoko(Caoguo) 10 g, Cortex Magnoliae Officinalis(Houpu) 10 g, Rhizoma Anemarrhenae (Zhimu)10 g,Radix Scutellariae Baicalensis (Huangqin)10 g,Radix Bupleuri Chinensis (Chaihu)10 g,Radix Paeoniae Rubra (Chishao) 10 g, Fructus Forsythiae Suspensae (Lianqiao)15 g, Herba Artemisiae Annuae (Qinghao) 10 g, Rhizoma Atractylodis Lanceae (Cangzhu)10 g, Radix Glycyrrhizae (Gancao)5 g;  (c). Cold damp obstructing the lung pattern, recommended prescription:Rhizoma Atractylodis Lanceae(Cangzhu)15 g, Pericarpium Citri Reticulatae(Chenpi) 10 g, Cortex Magnoliae Officinalis(Houpu)10 g, Herba Agastaches Rugosa(Huoxiang) 10 g, Fructus Tsaoko (Caoguo) 6 g, Herba Ephedra Sinica(Mahuang)6 g, Rhizoma et Radix Notopterygii (Qianghuo)10 g, Rhizoma Zingiberis Recens(Shengjiang) 10 g,Semen Areca e(Binglang)10 g. |
| **Guohua Chen*, et al.***  **2020** | Mahuang Liujun decoction | Scierotium Poriae Cocos (Fuling), Radix Astragali(Huangqi), Rhizoma Anemarrhenae Aspheloidis (Zhimu), Herba Pogostemonis (Huoxiang), Rhizoma Atractylodis (Cangzhu), JiangRhizoma Pinelliae (Fabanxia), Radix Glycyrrhizae (Gancao), Herba Ephedrae (Mahuang), Officinal Magnolia Bark(Houpo), Ramulus Cinnamomi Cassiae (Guizhi), Eupatorium fortune (Peilan), Radix Scutellariae Baicalensis (Huangqin), Amomum villosum(Sharen), Platycodi Radix (Jiegeng), Codonopsis pilosula (Dangshen) |
| **Jun Feng*, et al.***  **2021** | Shenhuang granule | Radix Ginseng (Renshen) 50g, Radix Et Rhizoma Rhei (Dahuang)40 g, Sargentodoxa cuneata stem (Hongteng)30g, Herba Taraxaci (Pugongying)30g, Radix LateralisPraeparatus Aconiti Carmichaeli (Fuzi)50 g, Whitmania pigra Whitman (Shuizhi) 6g |
| **Yuanyuan Wang*, et al.***  **2021** | CHM granule | Chaihu Jiedu granules /Fuzheng Jiufei granules |
| **Jing Zhang*, et al.***  **2022** | Shufeng Jiedu capsule | Rhizoma Polygoni Cuspidati (Huzhang), Fructus Forsythiae (Lianqiao), Radix Isatidis (Banlangen), Radix Bupleuri (Chaihu), Herba Patriniae (Baijiangcao), Herba Verbenae (Mabiancao), Rhizoma Phragmitis (Lugen), and Radix Glycyrrhizae (Gancao). |
| **Jianping Zhang*, et al.***  **2023** | Liushen tablet | Calculus bovis(Niuhuang), Muscat(Shexiang), Pearl powder(Zhengzhufen), cinobufagin venom toad (Chansu), Realgar(Xionghuang), borneol(Bingpian) |
| **Hai-Bo Hu*, et al.***  **2021** | Hejie Shenshi decoction | Radix Bupleuri (Chaihu), Radix Scutellariae Baicalensis (Huangqin), JiangRhizoma Pinelliae (Fabanxia), Scierotium Poriae Cocos (Fuling), Rhizoma Atractylodis Macrocephalae (Baizhu), Rhizoma Alismatis (Zexie), Semen Coicis (Yiyiren) |
| **Shuang Zhou*, et al.***  **2021** | Shenhuang granule | Radix Ginseng (Renshen) 50g, Radix Et Rhizoma Rhei (Dahuang)40 g, Sargentodoxa cuneata stem (Hongteng)30g, Herba Taraxaci (Pugongying)30g, Radix LateralisPraeparatus Aconiti Carmichaeli (Fuzi)50 g, Whitmania pigra Whitman (Shuizhi) 6g |
