# Supplementary material for: Effect of Chinese herbal medicine (CHM) as an adjunctive therapy in distinct stages of patients with COVID-19: A systematic review and meta-analysis
Source: PLoS One. 2025 Feb 13;20(2):e0318892. doi: 10.1371/journal.pone.0318892 (PMC11825027; doi:10.1371/journal.pone.0318892)
Supplement: S7 Table — (DOCX) [file pone.0318892.s010.docx]

**Supplementary Table S7. Subgroup and sensitivity analyses for the other main outcomes**

|  | **No. of Studies** | **MD/RR, 95%CI** | ***I*^2^** | **Q** | ***P*_subgroup_** |
| --- | --- | --- | --- | --- | --- |
| **Improvement rate of chest CT, n(%)** |  |  |  |  |  |
| **Age Group** ^a^ |  |  |  |  |  |
| ≤50 | 6 | 1.17(1.09,1.26) | 0% | 0.73 | 0.39 |
| >50 | 10 | 1.23(1.22,1.36) | 0% |  |  |
| **Study Design** |  |  |  |  |  |
| RCT | 8 | 1.15(1.06,1.24) | 0% | 2.43 | 0.12 |
| RCS | 8 | 1.27(1.14,1.4) | 0% |  |  |
| **Dosage Formulations** |  |  |  |  |  |
| decoction | 10 | 1.21(1.11,1.31) | 0% | 0.62 | 0.89 |
| granule | 3 | 1.19(1.04,1.36) | 16.20% |  |  |
| liquid | 2 | 1.11(0.92,1.35) | 0% |  |  |
| tablet | 1 | 1.22(1.01,1.48) | NA |  |  |
| **Usage** |  |  |  |  |  |
| 2 times daily | 11 | 1.22(1.1,1.34) | 0% | 1.01 | 0.6 |
| 3 times daily | 3 | 1.13(1.01,1.27) | 0% |  |  |
| 2~4 times daily | 1 | 1.2(0.9,1.58) | NA |  |  |
| **Study Course** |  |  |  |  |  |
| <14d | 3 | 1.11(0.98,1.25) | 0% | 0.94 | 0.33 |
| ≥14d | 6 | 1.2(1.08,1.33) | 0.20% |  |  |
| **Sex(male%)** |  |  |  |  |  |
| <50% | 9 | 1.28(1.16,1.4) | 0% | 2.69 | 0.1 |
| ≥50% | 8 | 1.15(1.06,1.25) | 0% |  |  |
| **Severe conversion rate of mild/moderate patients, n(%)** |  |  |  |  |  |
| **Age Group** ^a^ |  |  |  |  |  |
| ≤50 | 8 | 0.44(0.3,0.67) | 0% | 0.04 | 0.84 |
| >50 | 4 | 0.42(0.26,0.66) | 0% |  |  |
| **Study Design** |  |  |  |  |  |
| RCT | 8 | 0.46(0.33,0.65) | 0% | 0.27 | 0.87 |
| PCS | 1 | 0.46(0.22,0.97) | NA |  |  |
| RCS | 2 | 0.28(0.05,1.72) | 0% |  |  |
| **Dosage Formulations** |  |  |  |  |  |
| decoction | 5 | 0.37(0.2,0.66) | 0% | 0.76 | 0.69 |
| granule | 5 | 0.5(0.34,0.72) | 0% |  |  |
| liquid | 1 | 0.51(0.1,2.67) | NA |  |  |
| **Usage** |  |  |  |  |  |
| 2 times daily | 7 | 0.34(0.21,0.56) | 0% | 2.19 | 0.139 |
| 3 times daily | 3 | 0.56(0.37,0.84) | 0% |  |  |
| **Study Course** |  |  |  |  |  |
| <14d | 6 | 0.47(0.34,0.65) | 0% | 0.27 | 0.61 |
| ≥14d | 3 | 0.36(0.1,1.18) | 0% |  |  |
| **Sex(male%)** |  |  |  |  |  |
| <50% | 7 | 0.44(0.3,0.65) | 0% | 0.23 | 0.63 |
| ≥50% | 4 | 0.36(0.18,0.75) | 0% |  |  |
| **Mortality rate of severe/critical patients, n(%)** |  |  |  |  |  |
| **Study Design** |  |  |  |  |  |
| RCT | 3 | 0.53(0.42,0.67) | 0% | 2.29 | 0.13 |
| RCS | 8 | 0.41(0.31,0.53) | 18.10% |  |  |
| **Dosage Formulations** |  |  |  |  |  |
| decoction/capsule/granule | 1 | 0.4(0.16,0.98) | NA | 3.84 | 0.28 |
| decoction/capsule/granule/injection | 1 | 0.63(0.36,1.11) | NA |  |  |
| decoction | 5 | 0.36(0.25,0.52) | 26.30% |  |  |
| granule | 4 | 0.51(0.41,0.63) | 0% |  |  |
| **Sex(male%)** |  |  |  |  |  |
| <50% | 3 | 0.28(0.18,0.43) | 0% | 7.27 | <0.001^*^ |
| ≥50% | 8 | 0.54(0.45,0.65) | 0% |  |  |
| **Rate of fever reduction, n(%)** |  |  |  |  |  |
| **Age Group** ^a^ |  |  |  |  |  |
| ≤50 | 4 | 1.13(1.01,1.25) | 26.00% | 1.2 | 0.27 |
| >50 | 3 | 1.23(1.09,1.39) | 23.60% |  |  |
| **Study Design** |  |  |  |  |  |
| RCT | 4 | 1.13(1.01,1.26) | 27.60% | 0.75 | 0.69 |
| PCS | 1 | 1.15(1,1.31) | NA |  |  |
| RCS | 3 | 1.22(1.06,1.39) | 54.40% |  |  |
| **Dosage Formulations** |  |  |  |  |  |
| decoction/capsule/granule | 1 | 1.06(0.93,1.2) |  | 2.14 | 0.54 |
| decoction | 2 | 1.19(1.05,1.36) | 27.10% |  |  |
| granule | 4 | 1.18(1.04,1.33) | 48.00% |  |  |
| capsule | 1 | 1.17(0.95,1.43) | NA |  |  |
| **Usage** |  |  |  |  |  |
| 2 times daily | 4 | 1.17(1.06,1.29) | 49.60% | 1.35 | 0.25 |
| 3 times daily | 1 | 1.5(1-2.26) | NA |  |  |
| **Study Course** |  |  |  |  |  |
| <14d | 3 | 1.18(1.07,1.33) | 0.00% | 3.26 | 0.07 |
| ≥14d | 2 | 1.03(0.91,1.15) | 0.00% |  |  |
| **Sex(male%)** |  |  |  |  |  |
| <50% | 1 | 1.08(0.87,1.34) | NA | 0.29 | 0.59 |
| ≥50% | 5 | 1.15(1.05,1.26) | 39.30% |  |  |
| **Time to reduction of fever, mean ± SD** |  |  |  |  |  |
| **Age Group** ^a^ |  |  |  |  |  |
| ≤50 | 4 | -1.49(-2,-0.98) | 53.90% | 2.53 | 0.11 |
| >50 | 6 | -1.05(-1.25,-0.85) | 0% |  |  |
| **Study Design** |  |  |  |  |  |
| RCT | 3 | -1.27(-1.72,-0.83) | 38.90% | 0.05 | 0.82 |
| RCS | 8 | -1.21(-1.55,-0.87) | 18.90% |  |  |
| **Dosage Formulations** |  |  |  |  |  |
| decoction/capsule/granule | 1 | -0.5(-1.71,0.71) | NA | 1.72 | 0.63 |
| decoction | 5 | -1.31(-1.69,-0.93) | 61.50% |  |  |
| granule | 3 | -1.17(-1.85,-0.5) | 22.20% |  |  |
| injection | 2 | -1.39(-2.28,-0.5) | 0.00% |  |  |
| **Usage** |  |  |  |  |  |
| once daily | 1 | -1.27(-2.29,-0.25) | NA | 0.07 | 0.97 |
| 2 times daily | 5 | -1.25(-1.58,-0.92) | 62.80% |  |  |
| 3 times daily | 1 | -1.5(-3.41,0.41) | NA |  |  |
| **Sex(male%)** |  |  |  |  |  |
| <50% | 4 | -1.09(-1.41,-0.77) | 0.00% | 1.21 | 0.27 |
| ≥50% | 5 | -1.42(-1.92,-0.92) | 61.50% |  |  |
| **Improvement rate of cough, n(%)** |  |  |  |  |  |
| **Age Group** ^a^ |  |  |  |  |  |
| ≤50 | 3 | 2.23(1.56,3.19) | 0.00% | 6.52 | 0.01^*^ |
| >50 | 4 | 1.34(1.14,1.57) | 0.00% |  |  |
| **Study Design** |  |  |  |  |  |
| RCT | 3 | 1.96(1.44,2.68) | 8.00% | 6.12 | 0.047 |
| PCS | 1 | 1.22(0.98,1.51) | NA |  |  |
| RCS | 3 | 1.46(1.14,1.86) | 23.50% |  |  |
| **Dosage Formulations** |  |  |  |  |  |
| decoction | 3 | 1.4(1.14,1.72) | 44.20% | 4.63 | 0.1 |
| granule | 3 | 1.92(1.42,2.6) | 10.00% |  |  |
| liquid | 1 | 1.24(0.94,1.63) | NA |  |  |
| **Usage** |  |  |  |  |  |
| 2 times daily | 5 | 1.62(1.34,1.96) | 57.30% | 1.35 | 0.24 |
| 3 times daily | 2 | 1.34(1.06,1.73) | 0.00% |  |  |
| **Study Course** |  |  |  |  |  |
| <14d | 3 | 1.38(1.13,1.68) | 19.60% | 4,7 | 0.03 |
| ≥14d | 1 | 2.67(1.52,4.69) | NA |  |  |
| **Sex(male%)** |  |  |  |  |  |
| <50% | 1 | 1.24(0.94,1.63) | NA | 5.05 | 0.02^*^ |
| ≥50% | 4 | 1.95(1.47,2.59) | 0.00% |  |  |
| **Time to improvement of cough, mean ± SD** |  |  |  |  |  |
| **Age Group** ^a^ |  |  |  |  |  |
| ≤50 | 3 | -2.13(-2.83,-1.43) | 47.90% | 0.2 | 0.65 |
| >50 | 4 | -1.86(-2.81,-0.9) | 8.50% |  |  |
| **Study Design** |  |  |  |  |  |
| RCT | 1 | -1.44(-2.39,-0.49) | NA | 1.94 | 0.16 |
| RCS | 7 | -2.18(-2.62,-1.75) | 0.00% |  |  |
| **Dosage Formulations** |  |  |  |  |  |
| decoction | 5 | -2.07(-2.5,-1.65) | 0.00% | 0.15 | 0.93 |
| granule | 2 | -2.3(-7.56,2.97) | 68.70% |  |  |
| liquid | 1 | -1.82(-3.09,-0.55) | NA |  |  |
| **Usage** |  |  |  |  |  |
| 2 times daily | 4 | -2.21(-2.94,-1.49) | 46.60% | 0.28 | 0.6 |
| 3 times daily | 1 | -1.82(-3.1,-0.55) | NA |  |  |
| **Sex(male%)** |  |  |  |  |  |
| <50% | 4 | -2.21(-2.66,-1.57) | 13.20% | 0.06 | 0.81 |
| ≥50% | 4 | -2.(-2.79,-1.33) | 20.90% |  |  |
| **Improvement rate of fatigue, n(%)** |  |  |  |  |  |
| age |  |  |  |  |  |
| ≤50 | 2 | 1.04(0.96,1.13) | 0.00% | 0.37 | 0.54 |
| >50 | 5 | 1.08(1-1.15) | 28.70% |  |  |
| **Study Design** |  |  |  |  |  |
| RCT | 4 | 1.05(0.99,1.11) | 33.10% | 1.84 | 0.4 |
| PCS | 1 | 1.22(0.98,1.51) | NA |  |  |
| RCS | 2 | 1.03(0.92,1.15) | 0.00% |  |  |
| **Dosage Formulations** |  |  |  |  |  |
| decoction/tablet | 1 | 1.05(0.98,1.12) | NA | 2.83 | 0.24 |
| granule | 5 | 1.09(1-1.18) | 29.00% |  |  |
| liquid | 1 | 1/05(0.98,1.12) | NA |  |  |
| **Usage** |  |  |  |  |  |
| 2 times daily | 2 | 1.06(0.98,1.14) | 67.90% | 0.25 | 0.62 |
| 3 times daily | 4 | 1.09(1,1.18) | 11.30% |  |  |
| **Study Course** |  |  |  |  |  |
| <14d | 3 | 1.05(1-1.11) | 55.40% | 0.31 | 0.57 |
| ≥14d | 3 | 1.09(0.98-1.2) | 27.60% |  |  |
| **Sex(male%)** |  |  |  |  |  |
| <50% | 5 | 1.04(0.99,1.09) | 23.70% | 1.81 | 0.18 |
| ≥50% | 2 | 1.35(0.93,1.95) | 0.00% |  |  |
| **Improvement rate of breathless, n(%)** |  |  |  |  |  |
| **Age Group** ^a^ |  |  |  |  |  |
| ≤50 | 3 | 2.08(1.39,3.11) | 0.00% | 0 | 0.98 |
| >50 | 2 | 2.06(1.06,4.01) | 0.00% |  |  |
| **Study Design** |  |  |  |  |  |
| RCT | 2 | 1.96(1.2,3.22) | 0.00% | 0.09 | 0.77 |
| RCS | 3 | 2.18(1.34,3.54) | 0.00% |  |  |
| **Dosage Formulations** |  |  |  |  |  |
| decoction | 2 | 1.58(0.96,2.58) | 0.00% | 1.57 | 0.21 |
| granule | 3 | 2.44(1.52,3.9) | 0.00% |  |  |
| **Usage** |  |  |  |  |  |
| 2 times daily | 4 | 1.98(1.39,2.84) | 0.00% | 0.49 | 0.49 |
| 3 times daily | 1 | 3.21(0.87,11.9) | NA |  |  |

^*^Statistically significant subgroup effect sizes, ascertained as *P*_subgroup_ (χ2 test) <0.05.

^a^ Studies categorized by age group depending on mean.

Abbreviation: MD: difference in mean; RR: ratio risk; CI: confidence interval; SD: standard deviation
