# Supplementary material for: Effect of Chinese herbal medicine (CHM) as an adjunctive therapy in distinct stages of patients with COVID-19: A systematic review and meta-analysis
Source: PLoS One. 2025 Feb 13;20(2):e0318892. doi: 10.1371/journal.pone.0318892 (PMC11825027; doi:10.1371/journal.pone.0318892)
Supplement: S8 Table — (DOCX) [file pone.0318892.s011.docx]

**Supplementary Table S8. Quality evaluation of outcome indicators**

| **Outcome** | **Study** | **Course** | **Subjects**  **(ICW/CWM)** | **MD/OR(95%CI)** | **In favor of ICW therapy** | **Grade** |
| --- | --- | --- | --- | --- | --- | --- |
| **Improvement rate of chest CT, n(%)** | **YU Ping*, et al.* 2020** | 7d | 147/148 | 1.10(0.94,1.30) | yes | moderate^1^ |
|  | **LUO Zhihui*, et al.* 2021** | NA | 26/26 | 1.06(0.72,1.55) | yes | low^1,2^ |
|  | **ZENG Xianhong*, et al.* 2020** | NA | 104/125 | 1.26(1.10,1.43) | yes | moderate^1^ |
|  | **YANG Mingbo*, et al.* 2020** | 7d | 26/23 | 1.20(0.90,1.58) | yes | low^1,2^ |
|  | **WANG Lin*, et al.* 2020** | 14d | 40/40 | 1.16(0.97,1.38) | yes | moderate^1^ |
|  | **Jia Liu*, et al.* 2021** | NA | 94/96 | 1.28(1.04,1.58) | yes | moderate^1^ |
|  | **Ling Zhang*, et al.* 2022** | 14d | 72/72 | 1.22(1.01,1.48) | yes | moderate^1^ |
|  | **ZHOU Tong*, et al.* 2023** | 10d | 55/56 | 1.07(0.83,1.38) | yes | moderate^1^ |
|  | **Fei-Fei Lin*, et al.*2020** | 14d | 41/41 | 1.14(1.00,1.31) | yes | moderate^1^ |
|  | **XIA Wenguang*, et al.*2022** | 14d | 80/40 | 0.40(0.11,1.41) | yes | moderate^3^ |
|  | **QIN Lingxi*, et al.*2021** | NA | 523/40 | 1.54(0.95,2.50) | yes | moderate^1^ |
|  | **HUANG Donghui*, et al.*2021** | NA | 25/25 | 1.28(0.98,1.67) | yes | low^1,2^ |
|  | **YANG Qian20*, et al.*20** | NA | 51/52 | 1.53(1.01,2.31) | yes | moderate^1^ |
|  | **Chao Qun Huang*, et al.*2023** | NA | 294/16 | 1.10(0.82,1.46) | yes | moderate^1^ |
|  | **Fei He*, et al.*2022** | NA | 16/15 | 1.31(0.88,1.96) | yes | low^1,2^ |
|  | **Fei He*, et al.*2022** | NA | 16/15 | 1.31(0.88,1.96) | yes | low^1,2^ |
|  | **Yuanyuan Wang*, et al.*2021** | 28d | 43/43 | 2.79(0.59,13.16) | yes | moderate^2^ |
| **Severe conversion rate, n(%)** | **YE Ling*, et al.*2021** | 6d | 50/50 | 0.30(0.09,1.03) | yes | moderate^1^ |
|  | **LUO Zhihui*, et al.*2021** | NA | 26/26 | 0.33(0.01,7.82) | yes | low^1,2^ |
|  | **YU Ping*, et al.*2020** | 7d | 147/148 | 0.60(0.37,0.99) | yes | moderate^1^ |
|  | **WANG Lin*, et al, et al.*2020** | 14d | 40/40 | 0.14(0.01,2.68) | yes | moderate^1^ |
|  | **JI Dan*, et al.*2020** | NA | 28/22 | 0.26(0.03,2.35) | yes | low^1,2^ |
|  | **Jia Liu*, et al.*2021** | 14d | 94/96 | NA | yes | moderate^1^ |
|  | **Xin-Yi Zhang*, et al.*2021** | NA | 75/75 | 0.08(0.00,1.34) | yes | moderate^1^ |
|  | **XueDong An*, et al.*2021** | 14d | 92/31 | 0.67(0.13,3.50) | yes | high |
|  | **Congcong Zeng*, et al.*2021** | 14d | 30/29 | NA | yes | low^1,2^ |
|  | **Xiangru Xu*, et al.*2023** | 7d | 1411/1407 | NA | yes | moderate^1^ |
|  | **Chen Zhao*, et al.*2021** | 7d | 204/204 | 0.31(0.12,0.84) | yes | high |
|  | **Can Duan*, et al.*2020** | 5d | 82/41 | 0.45(0.20,1.02) | yes | high |
|  | **Fei-Fei Lin*, et al.*2020** | 14d | 41/41 | 0.20(0.01,4.04) | yes | high |
|  | **YAN Xiangyong*, et al.*2021** | 10d | 90/88 | 0.46(0.22,0.97) | yes | moderate^1^ |
|  | **ZHOU Tong*, et al.*2023** | 10d | 55/56 | 0.51(0.10,2.67) | yes | moderate^1^ |
| **Mortality rate, n(%)** | **QIN Lingxi*, et al.*2021** | NA | 523/40 | 0.40(0.16,0.98) | yes | moderate^1^ |
|  | **WANG Yuan*, et al.*2022** | 28d | 80/40 | 0.63(0.36,1.11) | yes | moderate^1^ |
|  | **YANG Qian*, et al.*2020** | NA | 51/52 | 0.70(0.36,1.36) | yes | moderate^1^ |
|  | **Jun Feng*, et al.*2021** | 14d | 57/54 | 0.51(0.43,0.73) | yes | moderate^1^ |
|  | **Guohua Chen*, et al.*2020** | NA | 156/156 | 0.27(0.16,0.45) | yes | moderate^1^ |
|  | **Chao Qun Huang*, et al.*2023** | NA | 523/40 | 0.40(0.16-,0.98) | yes | moderate^1^ |
|  | **Fei He*, et al.*2022** | NA | 16/15 | 0.19(0.01-,3.63) | yes | low^1,2^ |
|  | **Fei He*, et al.*2022** | NA | 16/15 | 0.19(0.01,3.63) | yes | low^1,2^ |
|  | **Yuanyuan Wang*, et al.*2021** | 28d | 43/43 | 0.22(0.05,0.97) | yes | high |
|  | **Shuang Zhou*, et al.*2021** | 14d | 57/54 | 0.51(0.35,0.73) | yes | moderate^1^ |
| **Negative time of nucleic acid, mean ± SD** | **ZENG Xianhong*, et al.*2020** | NA | 104/125 | -5.27(-5.79,4.75) | yes | moderate^1^ |
|  | **CHEN Fei*, et al.*2022** | NA | 30/45 | -6.50(-7.93,5.07) | yes | low^1,2^ |
|  | **Xin-Yi Zhang*, et al.*2021** | NA | 75/75 | -4.44(-6.04,2.84) | yes | moderate^1^ |
|  | **HUANG Donghui*, et al.*2021** | NA | 25/25 | -0.88(-1.39,0.37) | yes | moderate^1^ |
|  | **WANG Yuan*, et al.*2022** | 28d | 80/40 | -1.50(-1.40,0.39) | yes | moderate^1^ |
|  | **Chao Qun Huang*, et al.*2023** | NA | 246/6 | 2.00(-3.87,7.87) | no | low^1,3^ |
|  | **Hai-Bo Hu*, et al.*2021** | NA | 47/34 | -2.96(-5.60,-0.32) | yes | moderate^1^ |
|  | **Congcong Zeng*, et al.*2021** | 14d | 30/29 | 4.00(0.58,7.42) | no | low^1,2,3^ |
|  | **Xiangru Xu*, et al.*2023** | 7d | 1411/1407 | -1.00(-1.25,-0.75) | yes | moderate^1^ |
|  | **Fei-Fei Lin*, et al.*2020** | 14d | 41/41 | -3.85(-6.72,-0.44) | yes | moderate^1^ |
|  | **Chao Qun Huang*, et al.*2023** | NA | 547/26 | 5.00(3.73,6.27) | yes | moderate^1^ |
|  | **ZHOU Tong*, et al.*2023** | 10d | 55/56 | -1.53(-2.72,-0.34) | yes | moderate^1^ |
|  | **Jianping Zhang*, et al.*2023** | 7d | 91/90 | -0.53(-0.90,-0.16) | yes | high |
| **Rate of fever reduction, n(%)** | **QIN Lingxi*, et al.*2021** | NA | 231/23 | 1.06(0.93,1.20) | yes | moderate^1^ |
|  | **JI Dan*, et al.*2020** | NA | 26/21 | 1.38(1.00,1.91) | yes | moderate^1^ |
|  | **YAO Kaitao*, et al.*2020** | NA | 21/21 | 1.50(1.00,2.26) | yes | moderate^1^ |
|  | **Wuzhong Xiong*, et al.*202** | NA | 20/18 | 1.47(0.99,2.19) | yes | high |
|  | **XueDong An*, et al.*2021** | 14d | 32/10 | 1.08(0.87,1.34) | yes | high |
|  | **Yue Zhang*, et al.*2023** | 14d | 37/35 | 1.01(0.87,1.16) | yes | moderate^1^ |
|  | **YAN Xiangyong*, et al.*2021** | 10d | 90/88 | 1.15(1.01,1.31) | yes | moderate^1^ |
|  | **Jing Zhang*, et al.*2022** | 7d | 18/20 | 1.17(0.95,1.43) | yes | moderate^1^ |
| **Time to reduction of fever, mean ± SD** | **WANG Lin*, et al.*2020** | 14d | 40/40 | -1.49(-1.95,-1.03) | yes | moderate^1^ |
|  | **YAO Kaitao*, et al.*2020** | NA | 21/21 | -1.50(-3.41,0.41) | yes | low^1,2^ |
|  | **Jia Liu*, et al.*2021** | 14d | 94/96 | -1.00(-1.47-0.53) | yes | moderate^1^ |
|  | **Xin Yi Zhang*, et al.*2021** | NA | 75/75 | -1.27(-2.29,-0.25) | yes | moderate^1^ |
|  | **Zhijian Luo*, et al.*2021** | NA | 29/28 | -1.80(-3.64-0.04) | yes | moderate^2^ |
|  | **QIN Lingxi*, et al.*2021** | NA | 231/23 | -0.15(-1.36,1.06) | yes | low^1,3^ |
|  | **Hai-Bo Hu*, et al.*2021** | NA | 47/34 | -3.43(-6.49,-0.37) | yes | low^1,2^ |
|  | **LUO Zhihui*, et al.*2021** | NA | 26/26 | -2.53(-3.55,-1.51) | yes | moderate^1^ |
|  | **Jia Ke*, et al.*2020** | NA | 81/22 | -1.13(-2.06,-0.20) | yes | moderate^1^ |
|  | **QIAN Yu-jun*, et al.*2020** | NA | 170/130 | -1.02(-1.25,-0.79) | yes | moderate^1^ |
|  | **Hong-Ling Li*, et al.*2020** | NA | 169/22 | -1.08(-1.58,-0.58) | yes | moderate^1^ |
| **Improvement rate of cough, n(%)** | **LUO Zhihui*, et al.*2021** | NA | 15/10 | 2.17(0.99,4.75) | yes | moderate^1^ |
|  | **JI Dan*, et al.*2020** | NA | 24/19 | 1.78(1.00,3.17) | yes | moderate^1^ |
|  | **Wu-zhong Xiong*, et al.*2020** | NA | 17/18 | 1.97(1.04,3.72) | yes | high |
|  | **Ya-Hui Li*, et al.*2021** | 15d | 48/45 | 1.24(0.94,1.63) | yes | moderate^1^ |
|  | **Yue Zhang*, et al.*2023** | 14d | 35/29 | 2.67(1.52,4.69) | yes | moderate^1^ |
|  | **YAN Xiangyong*, et al.*2021** | 10d | 64/61 | 1.22(0.98,1.51) | yes | moderate^1^ |
|  | **Can Duan*, et al.*2020** | 5d | 62/28 | 1.54(0.97,1.51) | yes | moderate^1^ |
| **Time to improvement of cough, mean ± SD** | **LUO Zhihui*, et al.*2021** | NA | 26/26 | -2.95(-4.15,-1.75) | yes | moderate^1^ |
|  | **Hai-Bo Hu*, et al.*2021** | NA | 47/34 | 0.37(-3.74,4.48) | no | low^1,2,3^ |
|  | **Jia Ke*, et al.*2020** | NA | 67/17 | -1.79(-3.46,-0.12) | yes | moderate^1^ |
|  | **Jie Zhao*, et al.*2020** | NA | 15/:24 | -1.99(-3.05,-0.93) | yes | high |
|  | **Yuanyuan Wang*, et al.* 2021** | 28d | 38/38 | -5.00(-9.21,-0.79) | yes | moderate^2^ |
|  | **Hong-Ling Li*, et al.*2020** | NA | 169/22 | -2.18(-2.80,-1.56) | yes | moderate^1^ |
| **Improvement rate of breathless, n(%)** | **LUO Zhihui*, et al.*2021** | NA | 11/15 | 1.56(0.81,2.99) | yes | moderate^1^ |
|  | **JI Dan*, et al.*2020** | NA | 12/15 | 2.60(1.41,5.93) | yes | moderate^1^ |
|  | **YAO Kaitao*, et al.*2020** | NA | 7/9 | 3.21(0.87,1.90) | yes | moderate^1^ |
|  | **Wu-zhong Xiong*, et al.*2020** | NA | 10/8 | 1.60(0.75,3.42) | yes | high |
|  | **Yue Zhang*, et al.*2023** | 14d | 25/20 | 2.17(1.15,4.10) | yes | moderate^1^ |
| **Improvement rate of fatigue, n(%)** | **YAN Xiangyong*, et al.*2021** | 10d | 64/61 | 1.22(0.98,1.51) | yes | moderate^1^ |
|  | **YAO Kaitao*, et al.*2020** | NA | 21/21 | 1.35(0.47-3.89) | yes | low^1,2^ |
|  | **Chen Zhao*, et al.*2021** | 7d | 112/98 | 1.35(0.47-3.89) | yes | high |
|  | **Ya-Hui Li*, et al.*2021** | 15d | 49/45 | 1.00(0.94,1.06) | yes | moderate^1^ |
|  | **Can Duan*, et al.*2020** | 5d | 58/26 | 1.34(0.91,1.99) | yes | moderate^1^ |
|  | **Jianping Zhang*, et al.*2023** | 7d | 54/61 | 1.05(0.98,1.12) | yes | high |
|  | **XueDong An*, et al.*2021** | 14d | 65/19 | 1.02(0.82,1.27) | no | high |
| **WBC, mean ± SD** | **LUO Zhihui*, et al.*2021** | NA | 26/26 | 0.80(0.03,1.57) | yes | low^1,2^ |
|  | **YE Ling*, et al.*2021** | 6d | 50/50 | 0.03(-0.65,0.71) | no | moderate^1^ |
|  | **WANG Lin*, et al.*2020** | 14d | 40/49 | 0.61(-0.07,1.29) | yes | moderate^1^ |
|  | **Nannan Shi*, et al.*2021** | NA | 30/30 | -0.20(-1.23,0.83) | no | low^1,2^ |
|  | **WANG Yuan*, et al.*2022** | 28d | 80/40 | 1.09(-0.63,2.81) | yes | moderate^3^ |
|  | **Wen Long*, et al.*2020** | 7d | 20/20 | -0.21(-0.53,0.11) | no | moderate^2^ |
|  | **Jie Zhao*, et al.*2020** | NA | 15/34 | -0.39(-0.99,0.21) | no | high |
|  | **Jia Ke*, et al.*2020** | NA | 81/22 | -0.01(-0.84,0.82) | yes | moderate^1^ |
|  | **Yue Zhang*, et al.*2023** | 14d | 50/47 | -0.21(-0.76,0.34) | yes | moderate^1^ |
|  | **QIAN Yu-jun*, et al.*2020** | NA | 170/130 | 0.16(-0.18,0.50) | yes | moderate^1^ |
|  | **Hong-Ling Li*, et al.*2020** | NA | 169/22 | 0.39(-0.17,0.95) | yes | moderate^1^ |
|  | **ZHOU Tong*, et al.*2023** | 10d | 55/56 | 0.00(-0.97,0.97) | no | moderate^1^ |
|  | **Hai-Bo Hu*, et al.*2021** | NA | 47/34 | -1.44(-2.75,-0.13) | yes | low^1,3^ |
| **LYM, mean ± SD** | **LUO Zhihui*, et al.*2021** | NA | 26/26 | -0.17(-0.52,0.18) | yes | moderate^1^ |
|  | **YU Ping*, et al.*2020** | 7d | 147/148 | -0.11(-0.14,-0.08) | yes | moderate^1^ |
|  | **Jia Ke*, et al.*2020** | NA | 81/22 | -0.16(-0.35,0.03) | yes | moderate^1^ |
|  | **Yue Zhang*, et al.*2023** | 14d | 50/47 | -0.17(-0.43,0.09) | yes | moderate^1^ |
|  | **Hong-Ling Li*, et al.*2020** | NA | 169/22 | -0.19(-0.50,0.12) | yes | moderate^1^ |
|  | **ZHOU Tong*, et al.*2023** | 10d | 55/56 | -0.34(-0.62,-0.06) | yes | moderate^1^ |
|  | **WANG Yuan*, et al.*2022** | 28d | 80/40 | -0.21(-0.42,0.00) | yes | moderate^1^ |
|  | **YANG Qian*, et al.*2020** | NA | 51/52 | -0.19(-0.22,-0.16) | yes | moderate^1^ |
|  | **Wen Long*, et al.*2020** | 7d | 20/20 | -0.06(-0.26,0.14) | yes | moderate^1^ |
|  | **Wen Long*, et al.*2020** | 7d | 20/20 | -0.13(-0.29,0.03) | yes | moderate^1^ |
| **NEU, mean ± SD** | **HUANG Donghui*, et al.*2021** | NA | 25/25 | 2.20(1.46,2.94) | yes | low^1,3^ |
|  | **Yu Wang*, et al.*2021** | NA | 23/32 | 0.72(-0.28,1.72) | yes | low^1,3^ |
|  | **Hai-Bo Hu*, et al.*2021** | NA | 47/34 | -0.36(-1.56,0.84) | no | low^1,3^ |
|  | **Nannan Shi*, et al.*2021** | NA | 20/20 | -0.40(-1.64,0.84) | no | low^1,3^ |
|  | **ZHOU Tong*, et al.*2023** | 10d | 55/56 | 0.01(-0.58,0.60) | yes | moderate^3^ |
|  | **Jianping Zhang*, et al.*2023** | 7d | 91/90 | -0.84(-1.04,-0.64) | no | moderate^3^ |
| **CRP, mean ± SD** | **LUO Zhihui*, et al.*2021** | NA | 26/26 | 1.65(-12.06,15.36) | yes | low^1,2^ |
|  | **YU Ping*, et al.*2020** | 7d | 147/148 | 1.88(0.70,3.06) | yes | moderate^1^ |
|  | **YANG Mingbo*, et al.*2020** | 7d | 26/23 | -5.42(-17.07,6.23) | no | low^1,2^ |
|  | **CHEN Fei*, et al.*2022** | NA | 30/45 | 2.72(0.80,4.64) | yes | moderate^1^ |
|  | **Nannan Shi*, et al.*2021** | NA | 20/20 | -6.70(-25.94,12.54) | no | low^1,2^ |
|  | **Hong-Ling Li*, et al.*2020** | NA | 169/22 | 4.33(2.27,6.39) | yes | moderate^1^ |
|  | **ZHOU Tong*, et al.*2023** | 10d | 55/56 | 1.11(-6.00,8.22) | yes | moderate^1^ |
|  | **WANG Yuan*, et al.*2022** | 28d | 80/40 | -2.77(-19.50,13.96) | no | low^2,3^ |
|  | **Wen Long*, et al.*2020** | 7d | 20/20 | 8.00(4.69,11.31) | yes | moderate^3^ |
|  | **Yu Wang*, et al.*2021** | NA | 23/32 | -9.50(-22.99,3.99) | no | low^1,2,3^ |
|  | **Hai-Bo Hu*, et al.*2021** | NA | 47/34 | -0.09(-2.79,2.61) | yes | low^1,3^ |
| **Length of hospital stays, mean ± SD** | **HUANG Donghui*, et al.*2021** | NA | 25/25 | -1.40(-2.44,-0.36) | yes | low^1,3^ |
|  | **YANG Qian*, et al.*2020** | NA | 51/52 | 2.80(2.45,3.15) | yes | low^1,3^ |
|  | **Jie Zhao*, et al.*2020** | NA | 15/24 | -11.00(-31.72,9.72) | yes | moderate^3^ |
|  | **Jie Zhao*, et al.*2020** | NA | 29/28 | -2.30(-3.88,-0.72) | yes | moderate^3^ |
|  | **Zhi-Dan Lu*, et al.*2022** | NA | 15/14 | -2.94(-9.08,3.20) | yes | low^1,3^ |
|  | **Jun Feng*, et al.*2021** | 14d | 33/85 | -44.00(-57.33,-30.67) | yes | moderate^3^ |
|  | **LUO Zhihui*, et al.*2021** | NA | 26/26 | -2.48(-4.06,-0.90) | yes | low^1,3^ |
|  | **ZENG Xianhong*, et al.*2020** | NA | 104/125 | -4.72(-5.34,-4.10) | yes | moderate^3^ |
|  | **Nannan Shi*, et al.*2021** | NA | 20/20 | -1.70(-5.79,2.39) | yes | low^1,3^ |
|  | **Congcong Zeng*, et al.*2021** | 14d | 30/29 | 3.00(-0.43,6.43) | no | low^1,3^ |
|  | **Xiangru Xu*, et al.*2023** | 7d | 1411/1407 | -1.00(-1.28,-0.72) | yes | low^1,3^ |
|  | **Chen Zhao*, et al.*2021** | 7d | 204/204 | 0.50(-0.39,1.39) | yes | moderate^3^ |
|  | **Fei-Fei Lin*, et al.*2020** | 14d | 41/41 | -3.21(-6.08,-0.34) | yes | moderate^3^ |
|  | **QIAN Yu-jun*, et al.*2020** | NA | 170/130 | -5.42(-5.85,-4.99) | yes | moderate^1^ |
|  | **Hong-Ling Li*, et al.*2020** | NA | 169/22 | -4.92(-5.83,-4.01) | yes | moderate^1^ |
|  | **Zhi-Dan Lu*, et al.*2022** | NA | 15/16 | 5.22(-0.65,11.09) | no | moderate^1^ |

^1.^risk of bias; ^2.^ imprecision; ^3.^ inconsistency;^4.^ indirectness;^5.^ publication bias

### Definition of abbreviations: MD: mean difference; OR: odds ratio; ICW: integrated Chinese herbal medicine and western medicine therapy; CWM: conventional western medicine; WBC: white blood cell; LYM: lymphocyte; NEU: neutrophil; CRP: C-reactive protein
